# Supplementary figures and images for: Flexible Machine Learning Algorithms for Clinical Gait Assessment Tools
Source: Sensors (Basel). 2022 Jun 30;22(13):4957. doi: 10.3390/s22134957 (PMC9269679; doi:10.3390/s22134957)

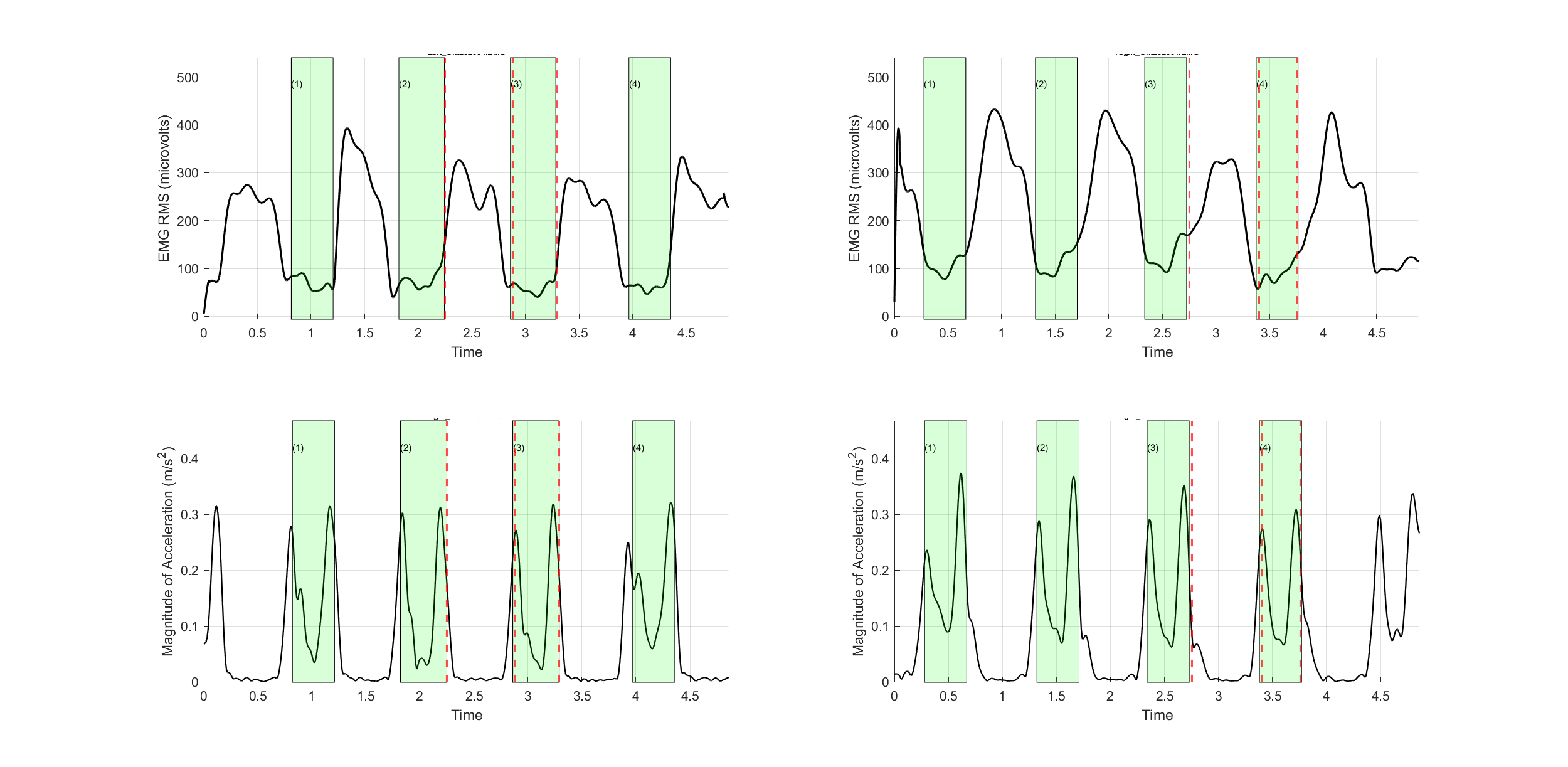

Supplement: Supplementary file 1 [file sensors-22-04957-s001.zip › Part 1 - 3D CGA historic patient data partitions/Figure_10202004.png]

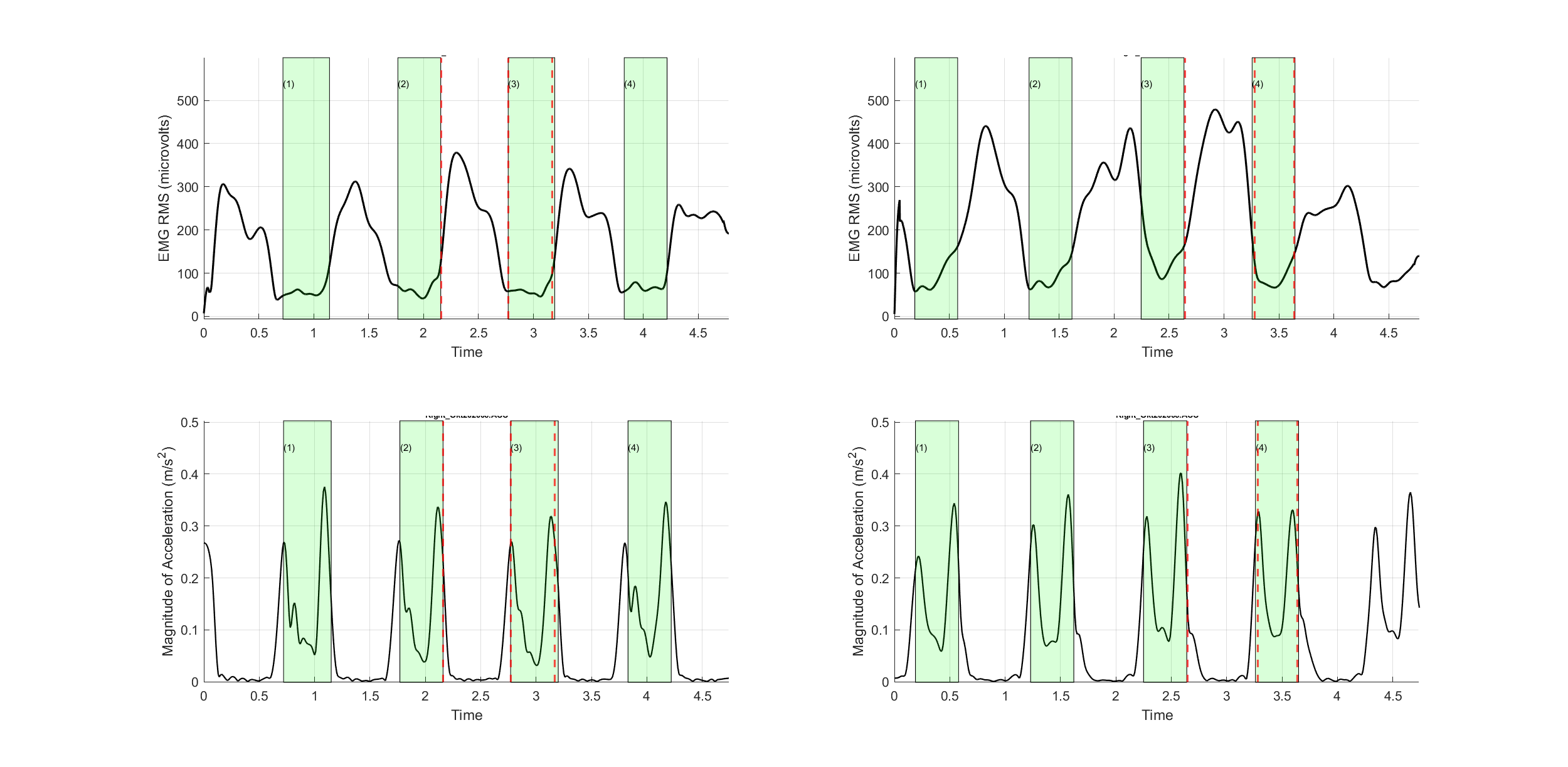

Supplement: Supplementary file 1 [file sensors-22-04957-s001.zip › Part 1 - 3D CGA historic patient data partitions/Figure_10202006.png]

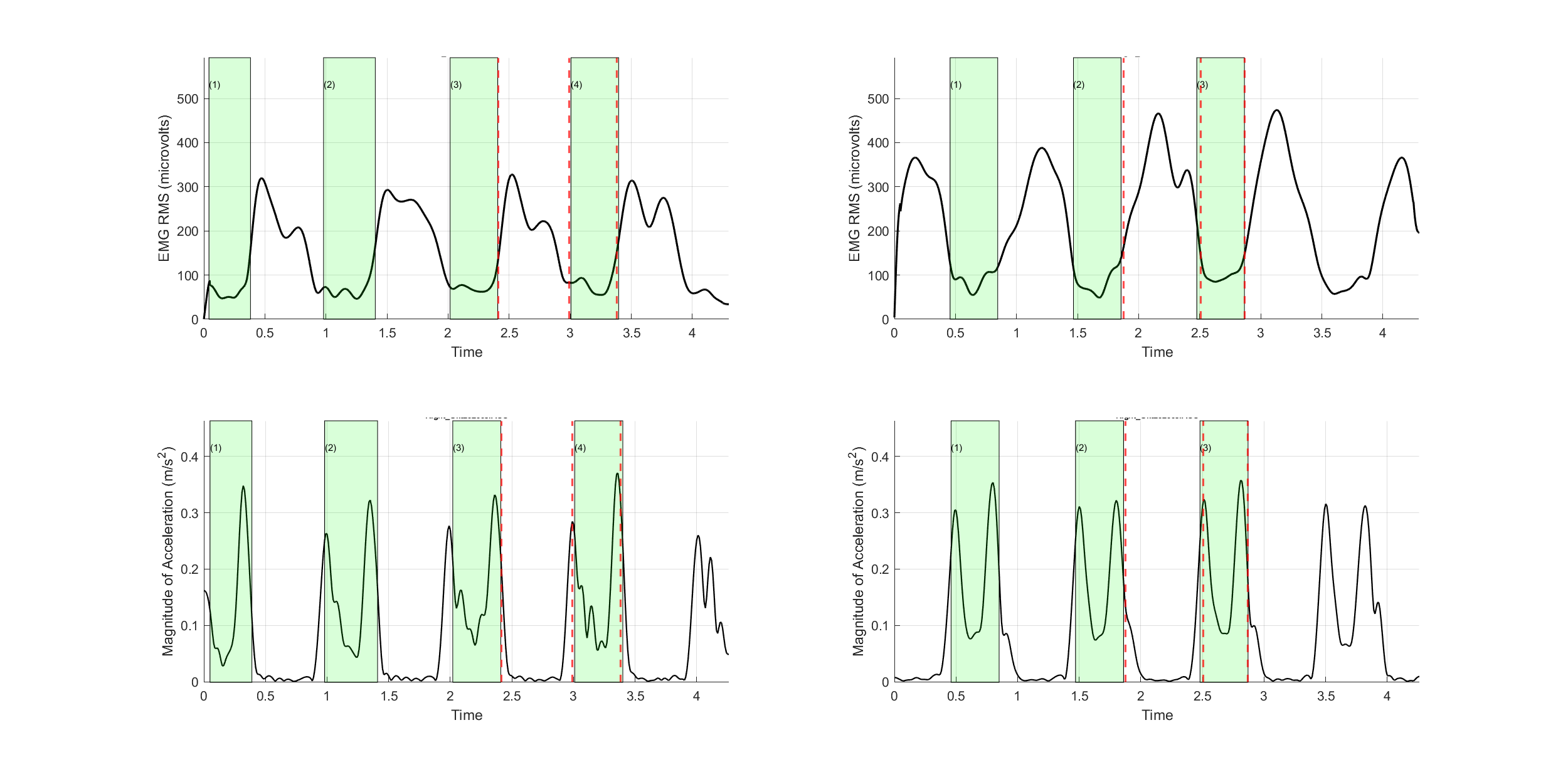

Supplement: Supplementary file 1 [file sensors-22-04957-s001.zip › Part 1 - 3D CGA historic patient data partitions/Figure_10202008.png]

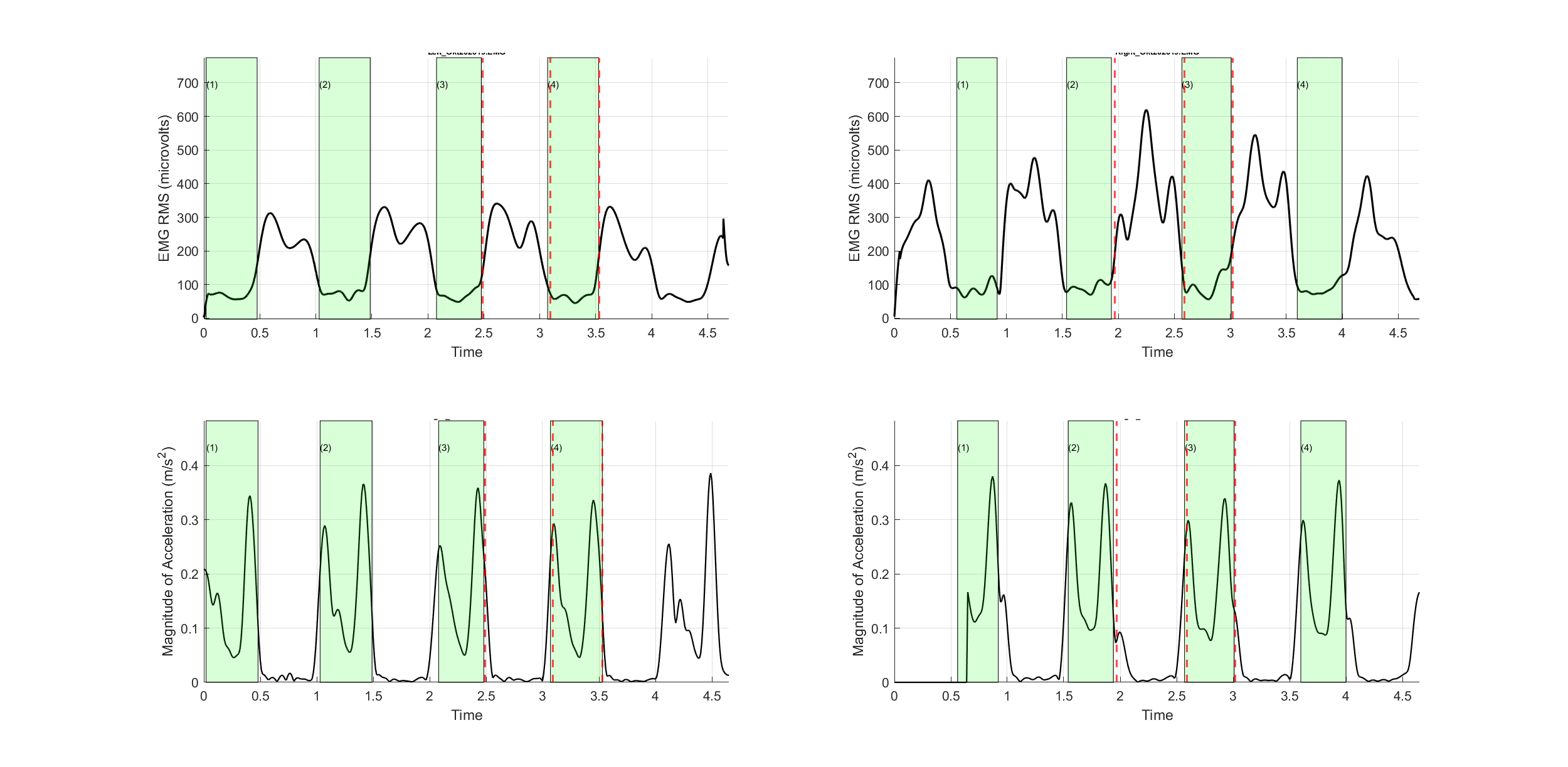

Supplement: Supplementary file 1 [file sensors-22-04957-s001.zip › Part 1 - 3D CGA historic patient data partitions/Figure_10202019.png]

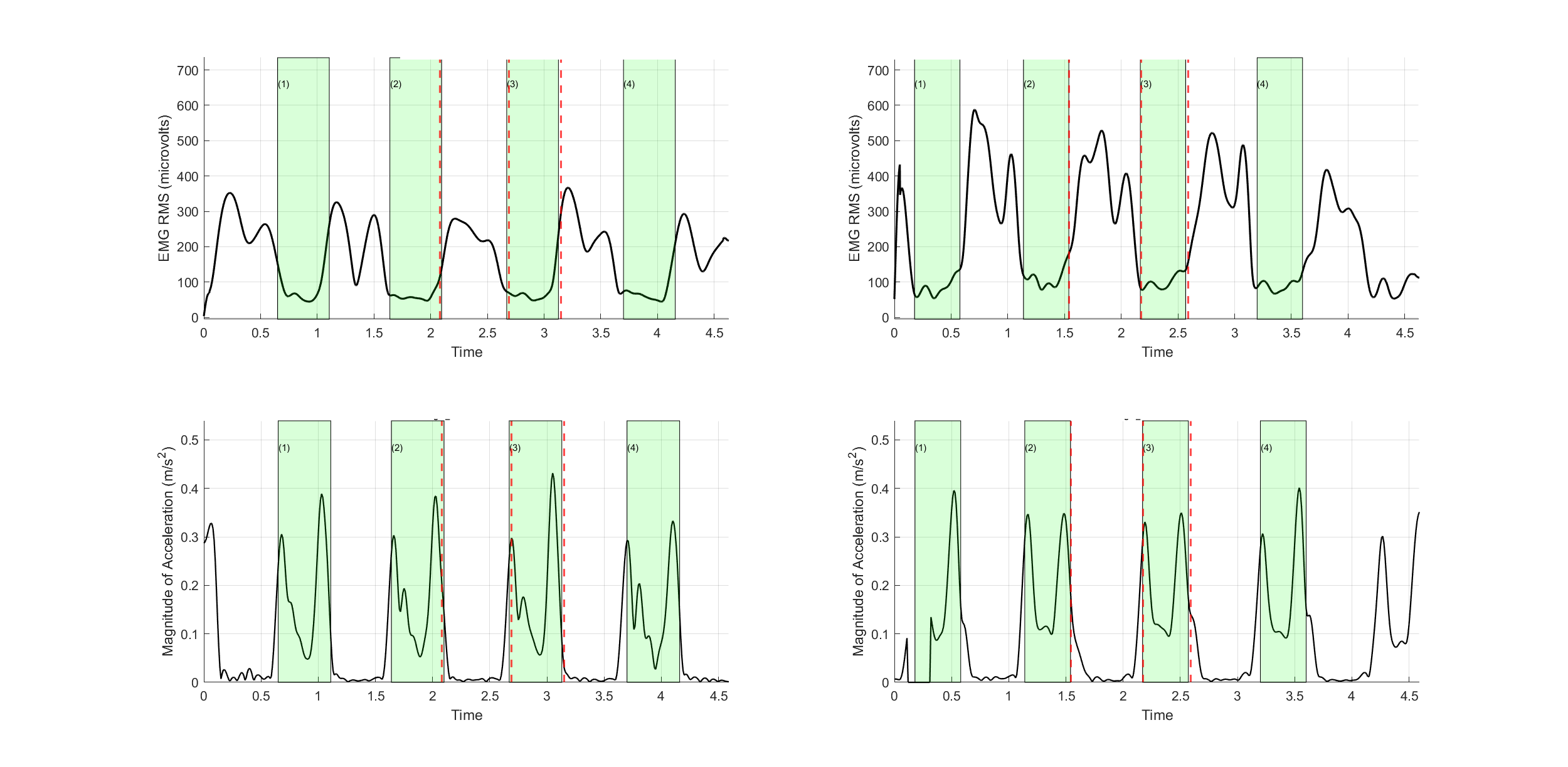

Supplement: Supplementary file 1 [file sensors-22-04957-s001.zip › Part 1 - 3D CGA historic patient data partitions/Figure_10202024.png]

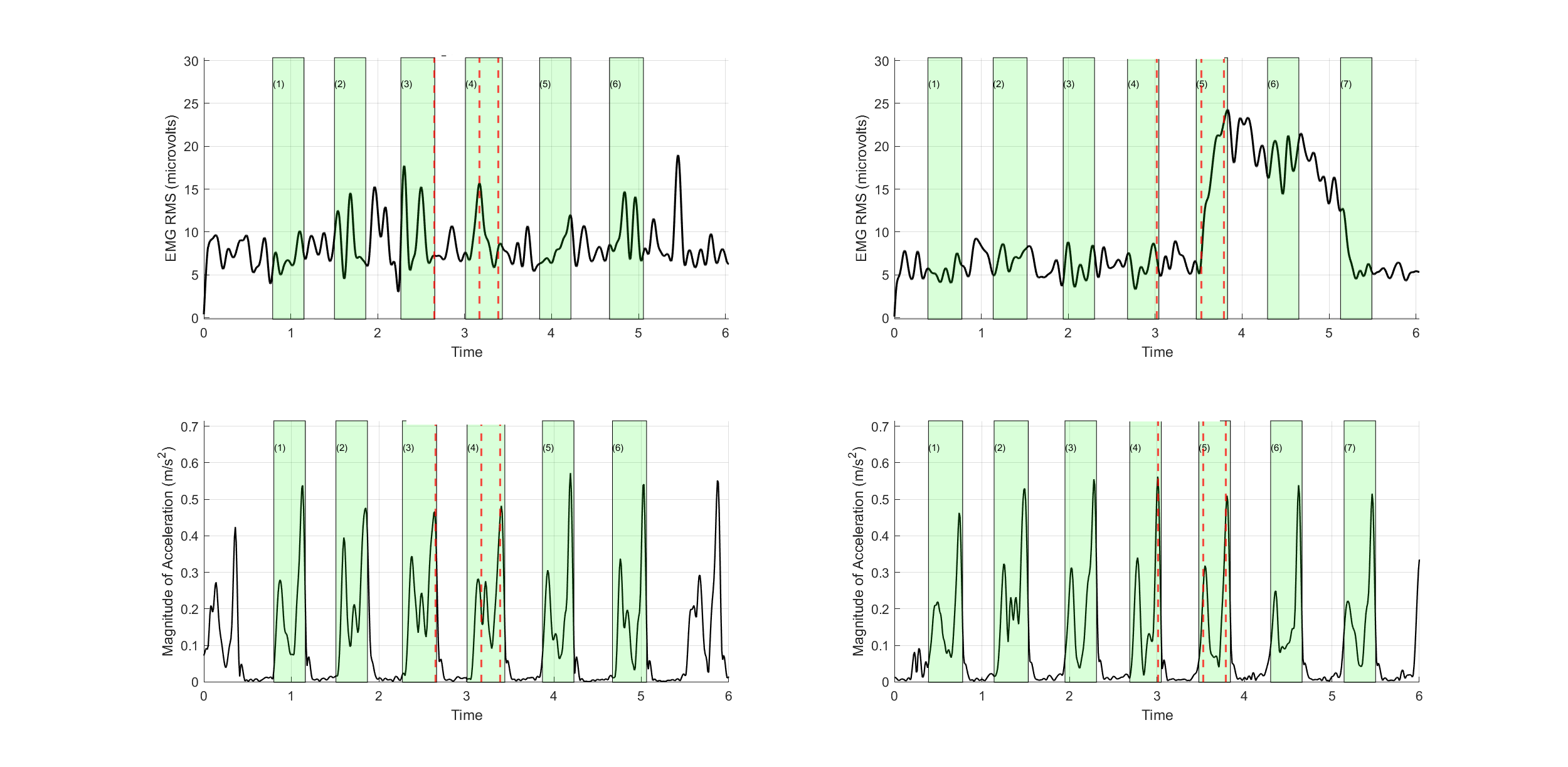

Supplement: Supplementary file 1 [file sensors-22-04957-s001.zip › Part 1 - 3D CGA historic patient data partitions/Figure_1202104.png]

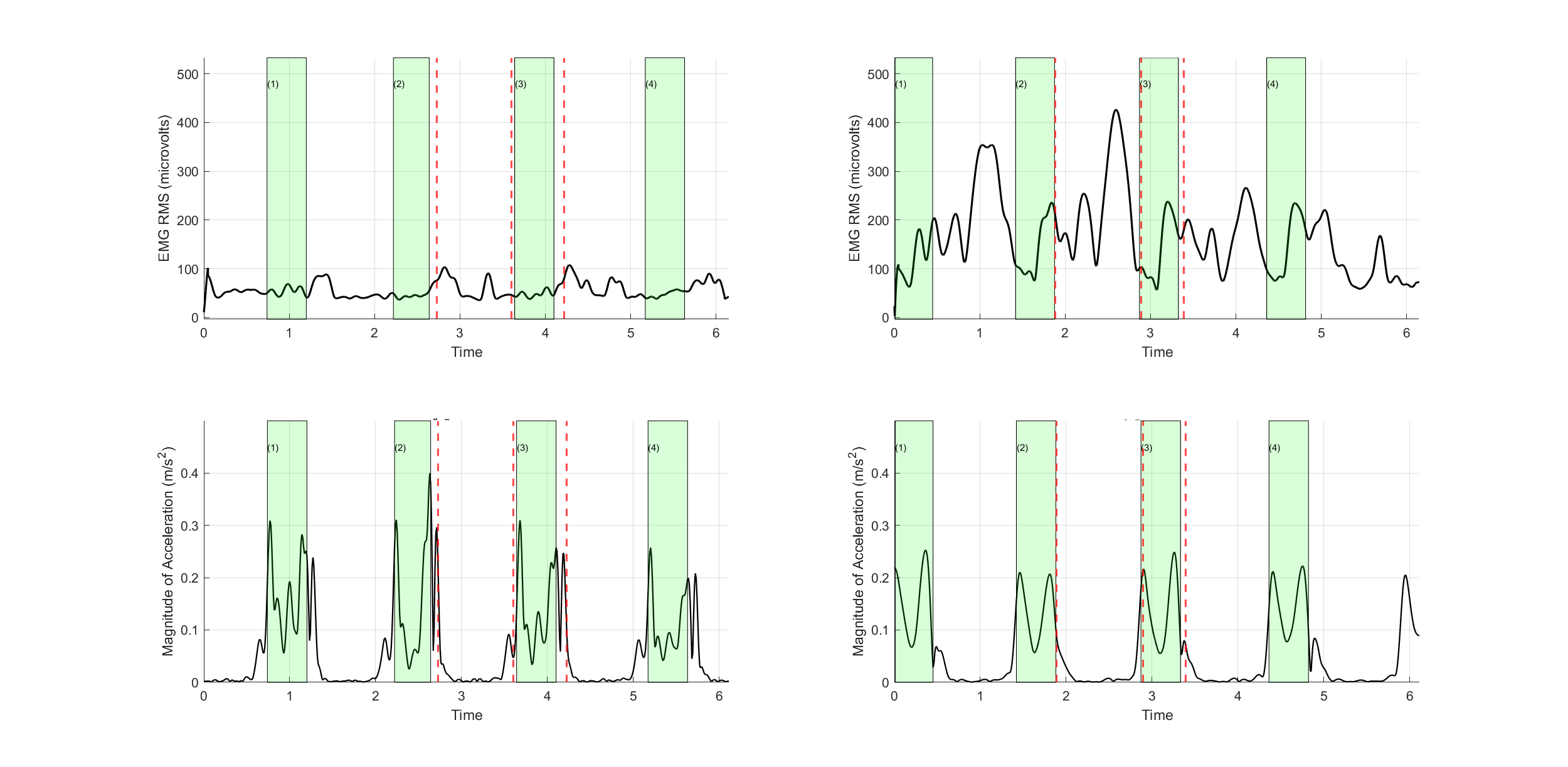

Supplement: Supplementary file 1 [file sensors-22-04957-s001.zip › Part 1 - 3D CGA historic patient data partitions/Figure_1202105.png]

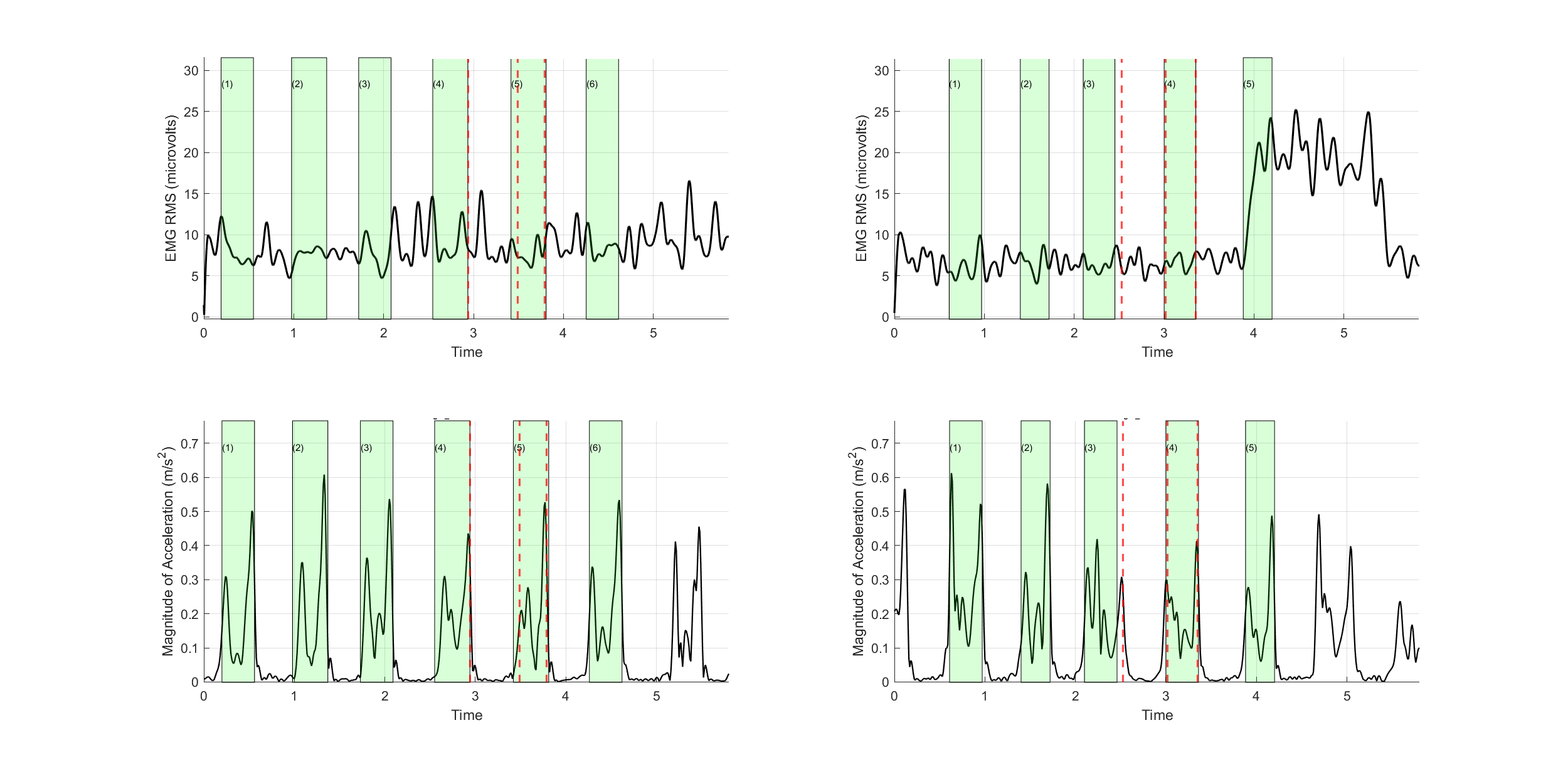

Supplement: Supplementary file 1 [file sensors-22-04957-s001.zip › Part 1 - 3D CGA historic patient data partitions/Figure_1202106.png]

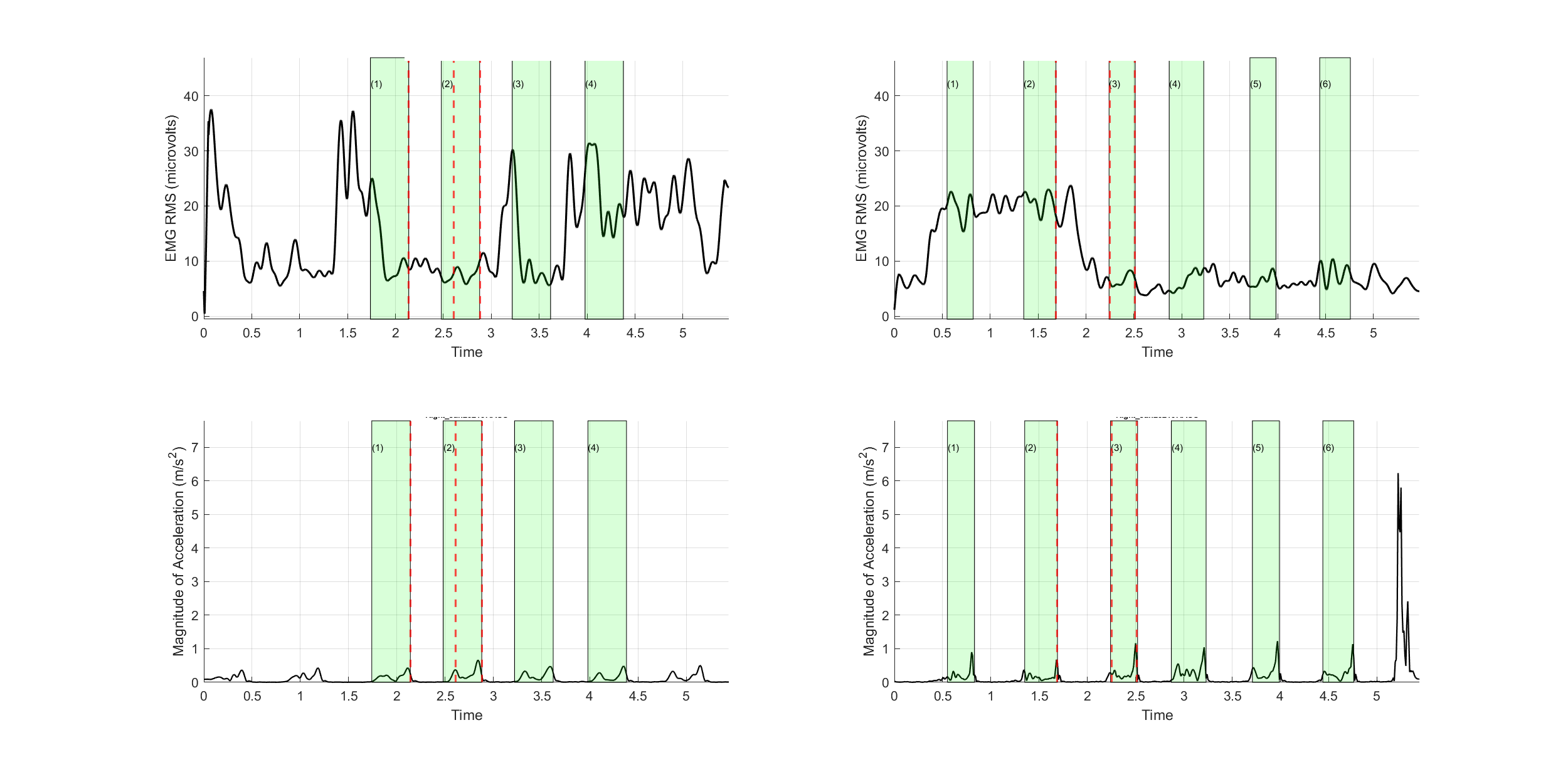

Supplement: Supplementary file 1 [file sensors-22-04957-s001.zip › Part 1 - 3D CGA historic patient data partitions/Figure_1202107.png]

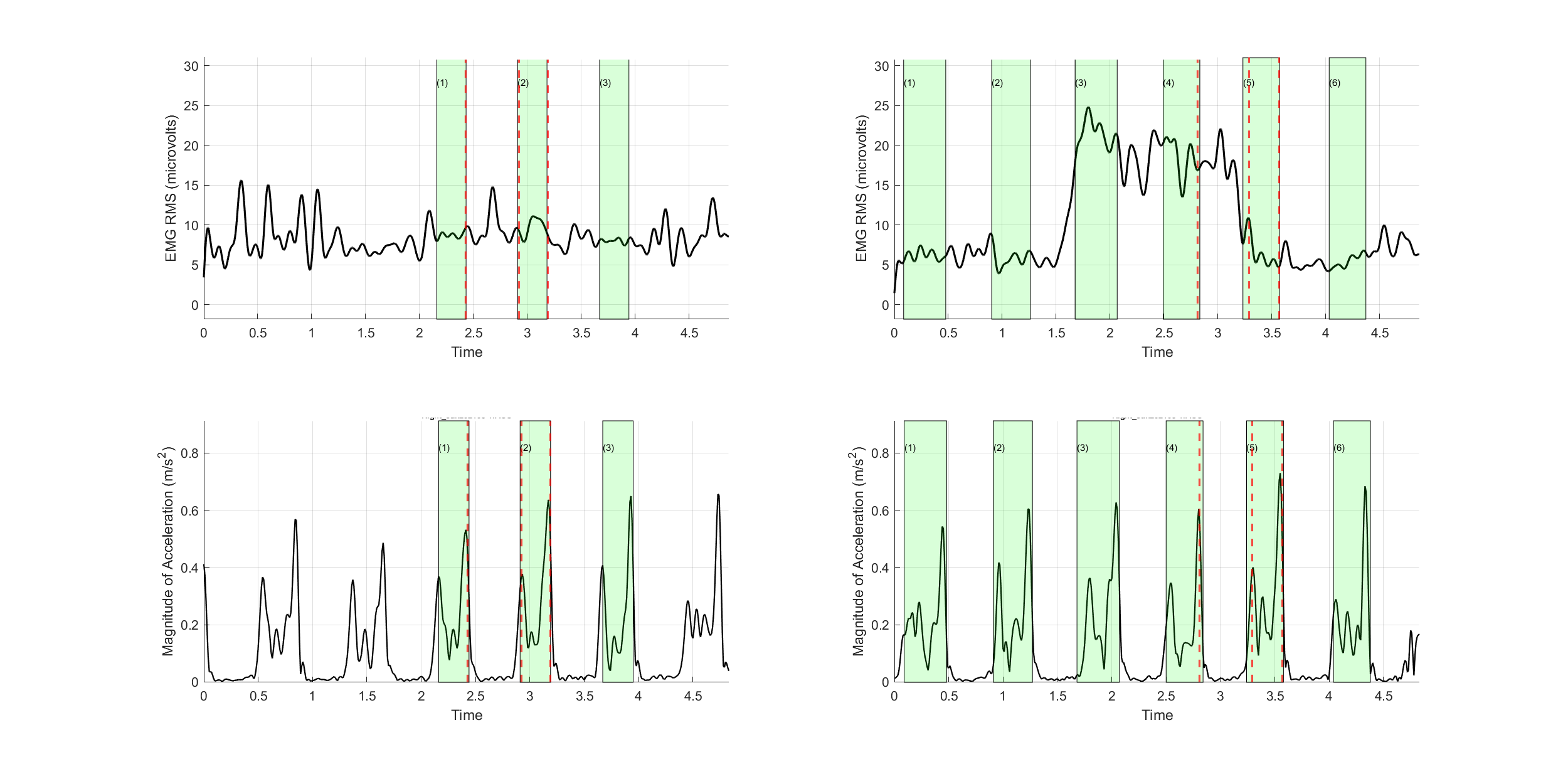

Supplement: Supplementary file 1 [file sensors-22-04957-s001.zip › Part 1 - 3D CGA historic patient data partitions/Figure_1202108-1.png]

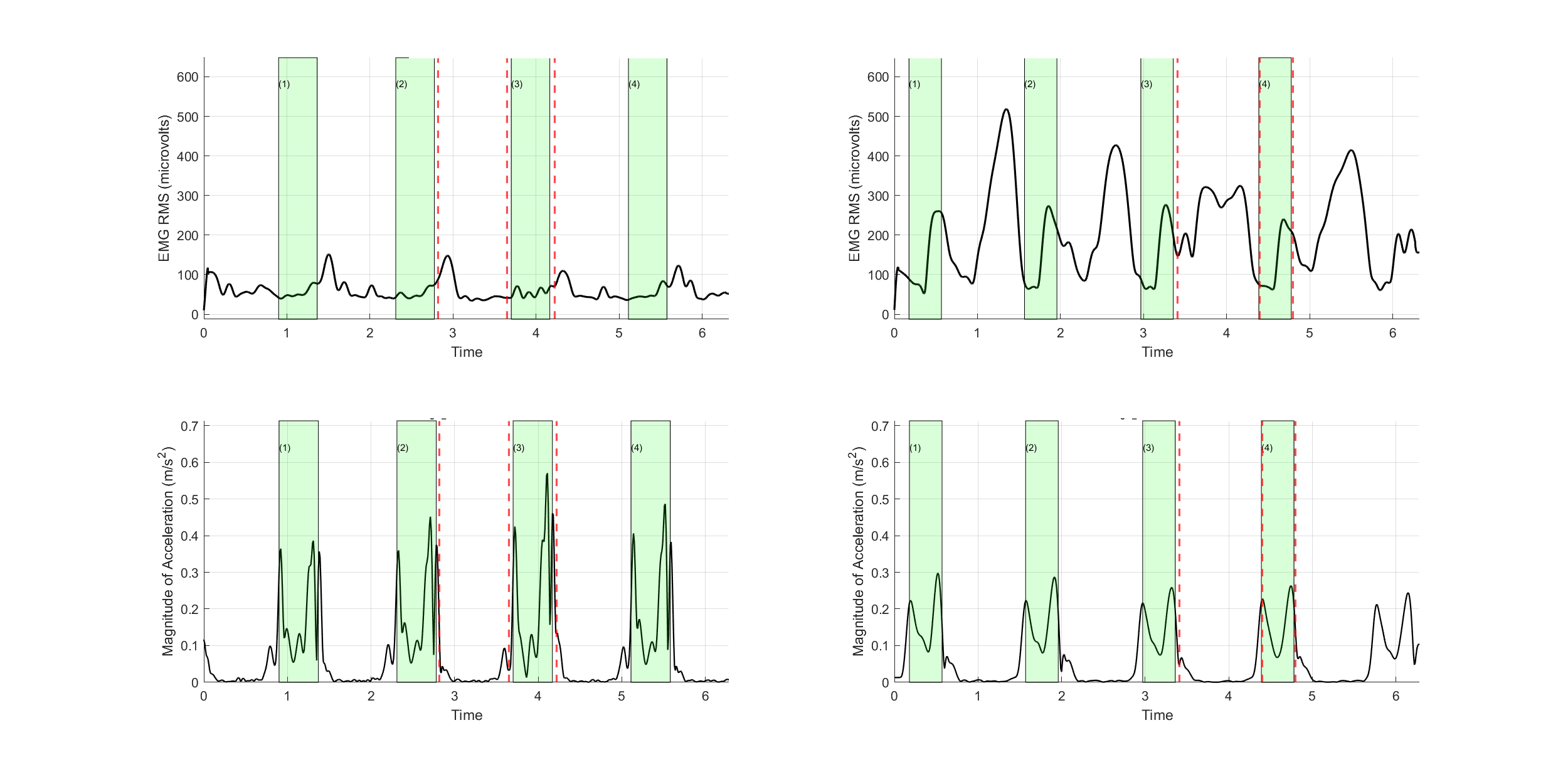

Supplement: Supplementary file 1 [file sensors-22-04957-s001.zip › Part 1 - 3D CGA historic patient data partitions/Figure_1202108-2.png]

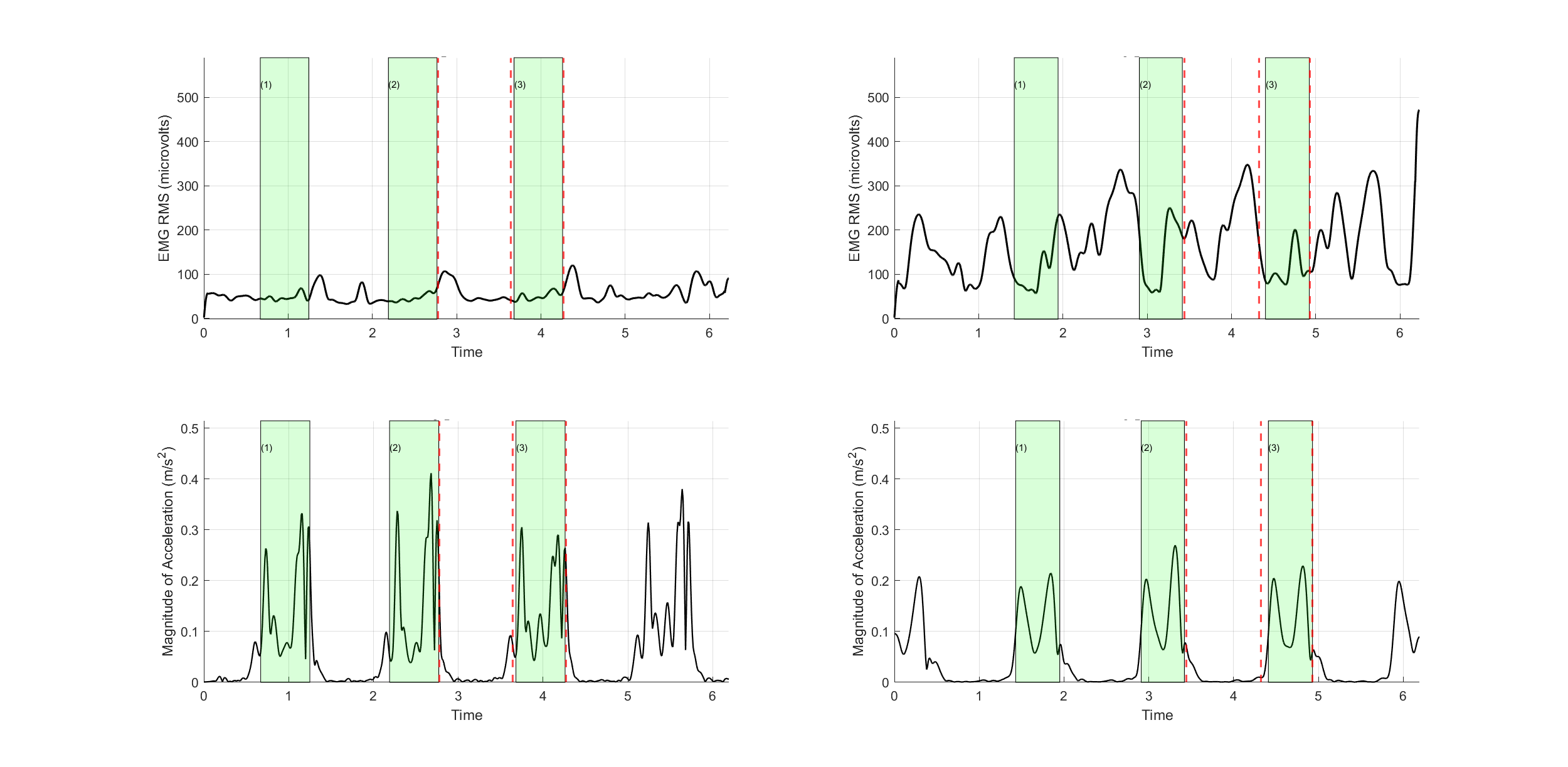

Supplement: Supplementary file 1 [file sensors-22-04957-s001.zip › Part 1 - 3D CGA historic patient data partitions/Figure_1202115.png]

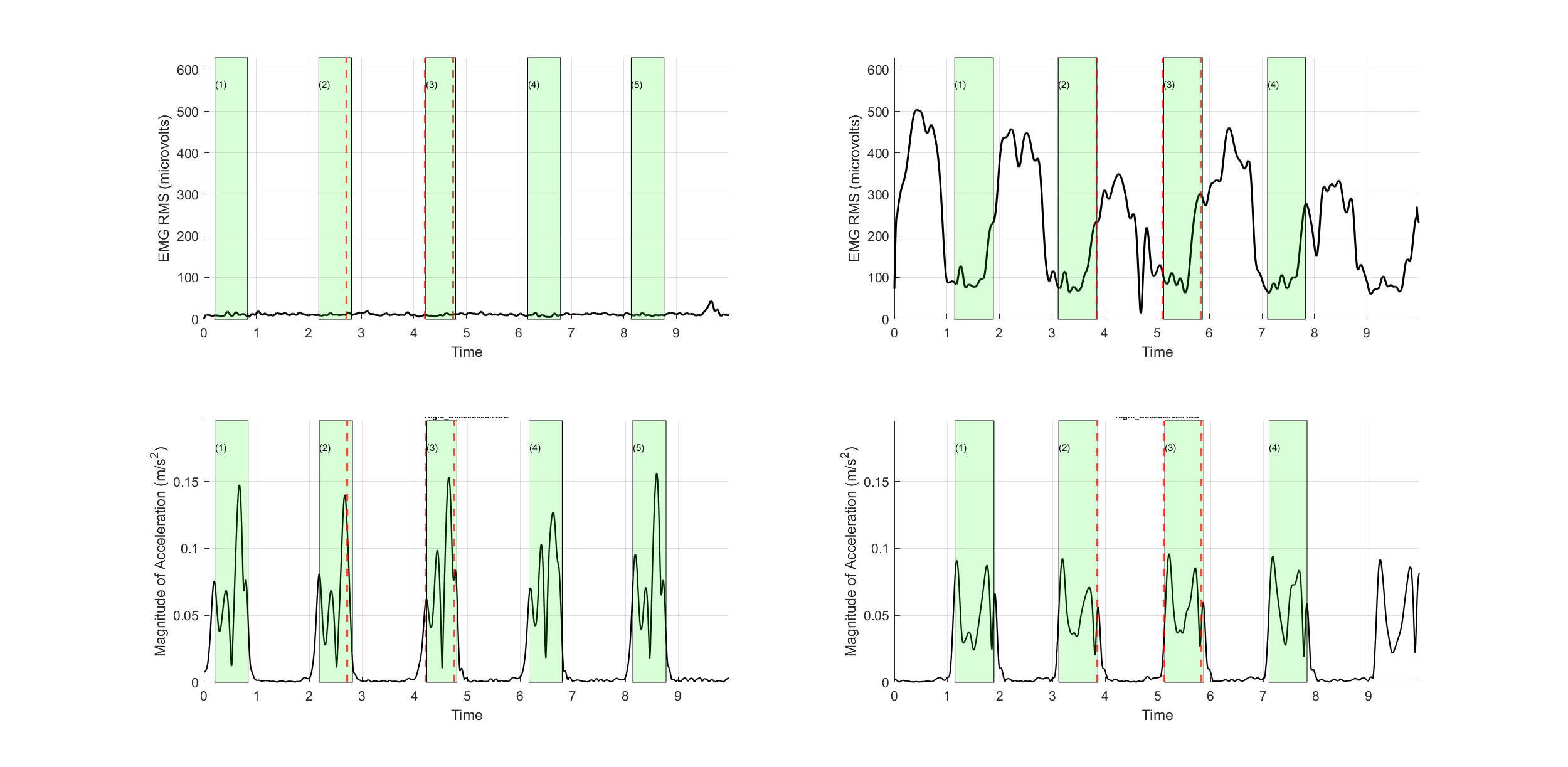

Supplement: Supplementary file 1 [file sensors-22-04957-s001.zip › Part 1 - 3D CGA historic patient data partitions/Figure_12202005.png]

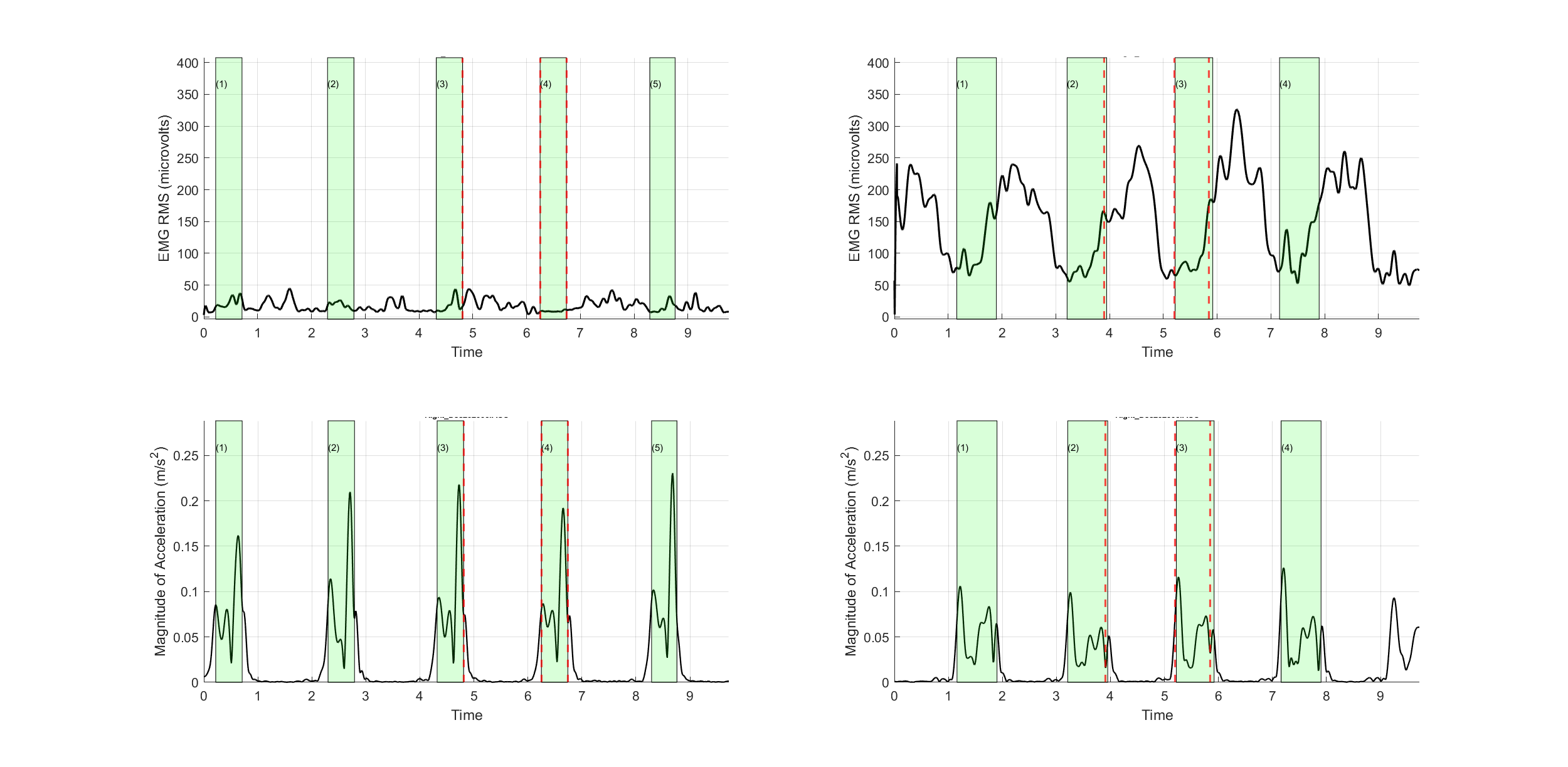

Supplement: Supplementary file 1 [file sensors-22-04957-s001.zip › Part 1 - 3D CGA historic patient data partitions/Figure_12202006.png]

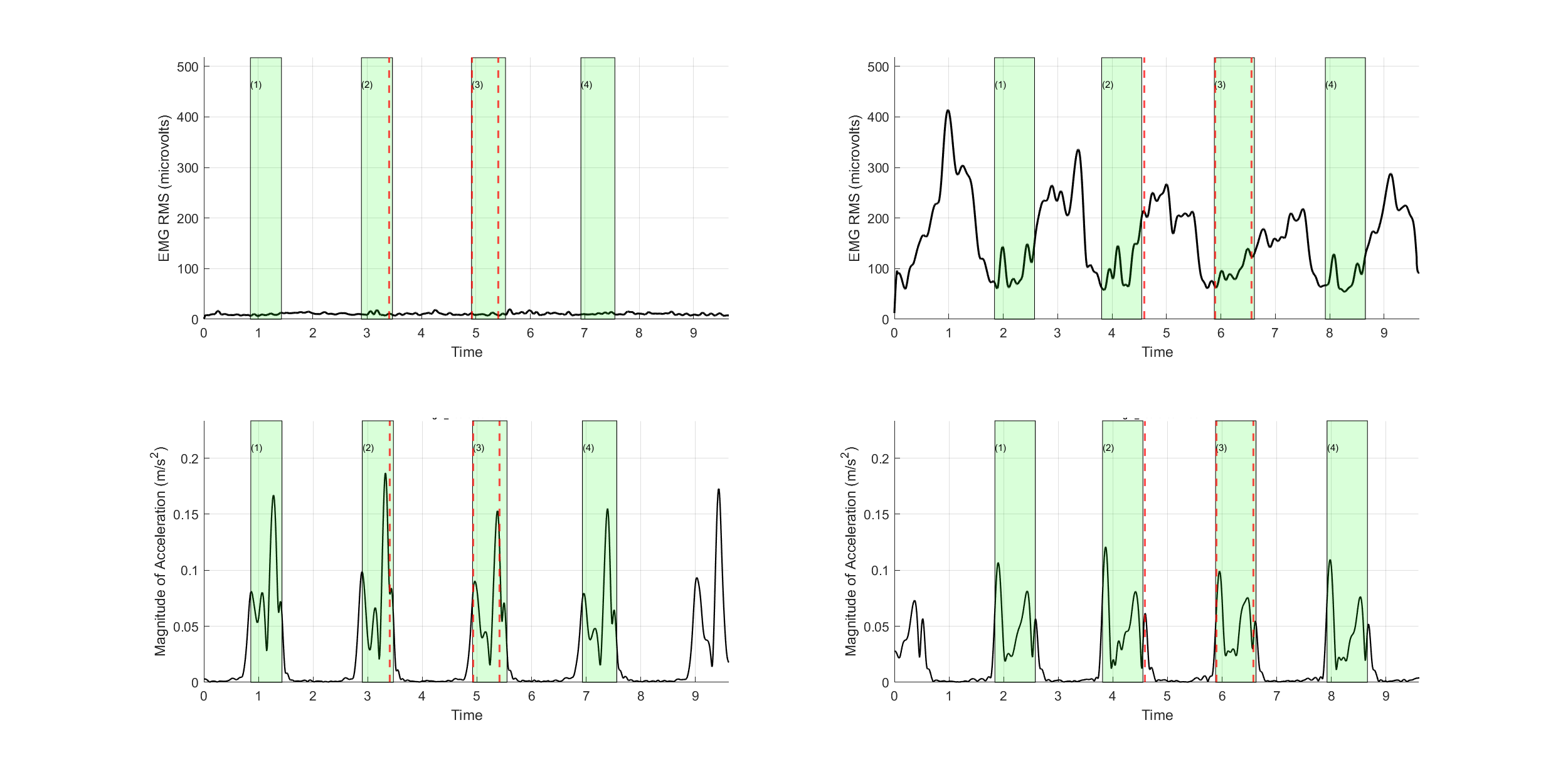

Supplement: Supplementary file 1 [file sensors-22-04957-s001.zip › Part 1 - 3D CGA historic patient data partitions/Figure_12202007.png]

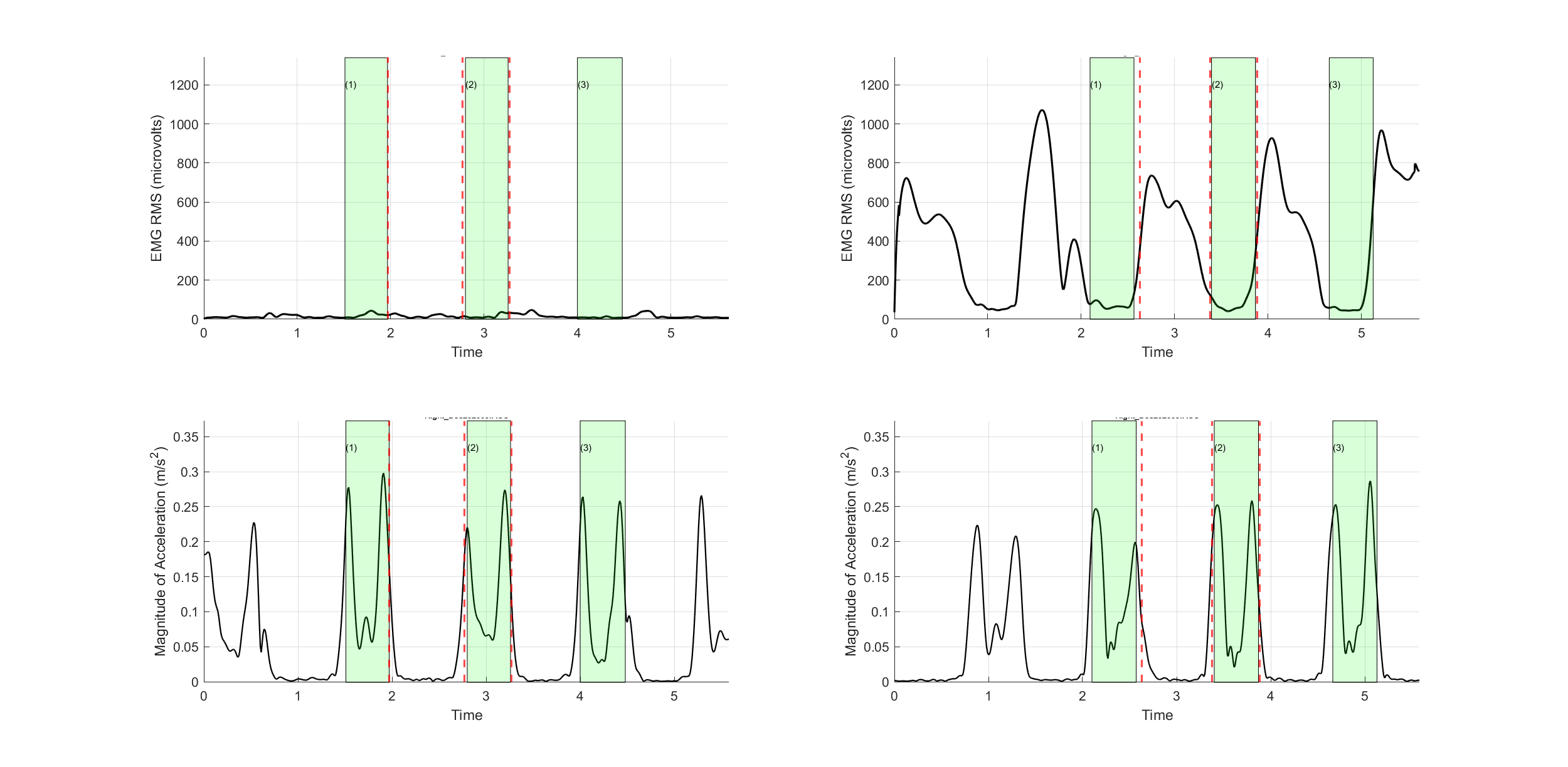

Supplement: Supplementary file 1 [file sensors-22-04957-s001.zip › Part 1 - 3D CGA historic patient data partitions/Figure_12202009.png]

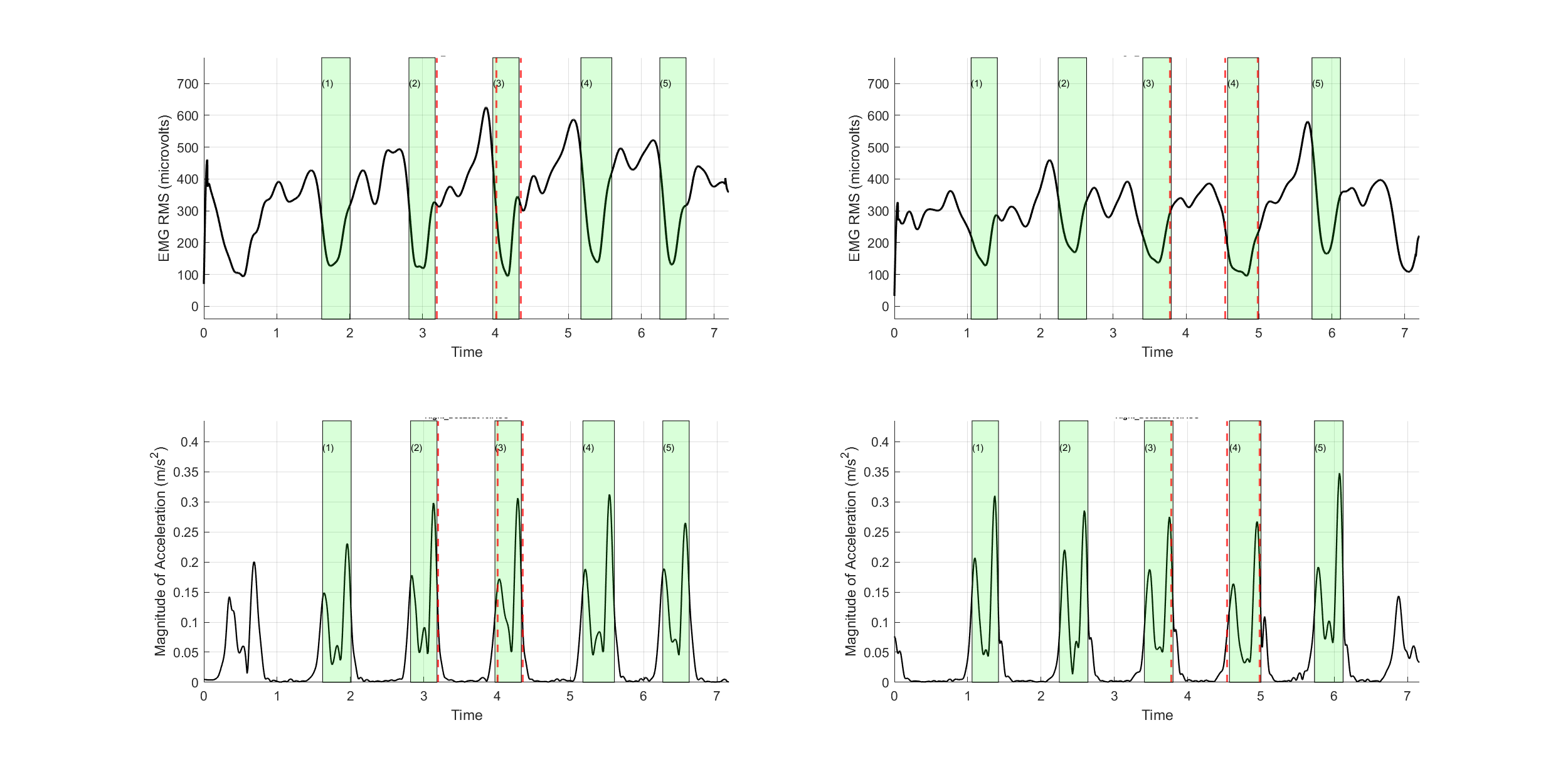

Supplement: Supplementary file 1 [file sensors-22-04957-s001.zip › Part 1 - 3D CGA historic patient data partitions/Figure_12202010.png]

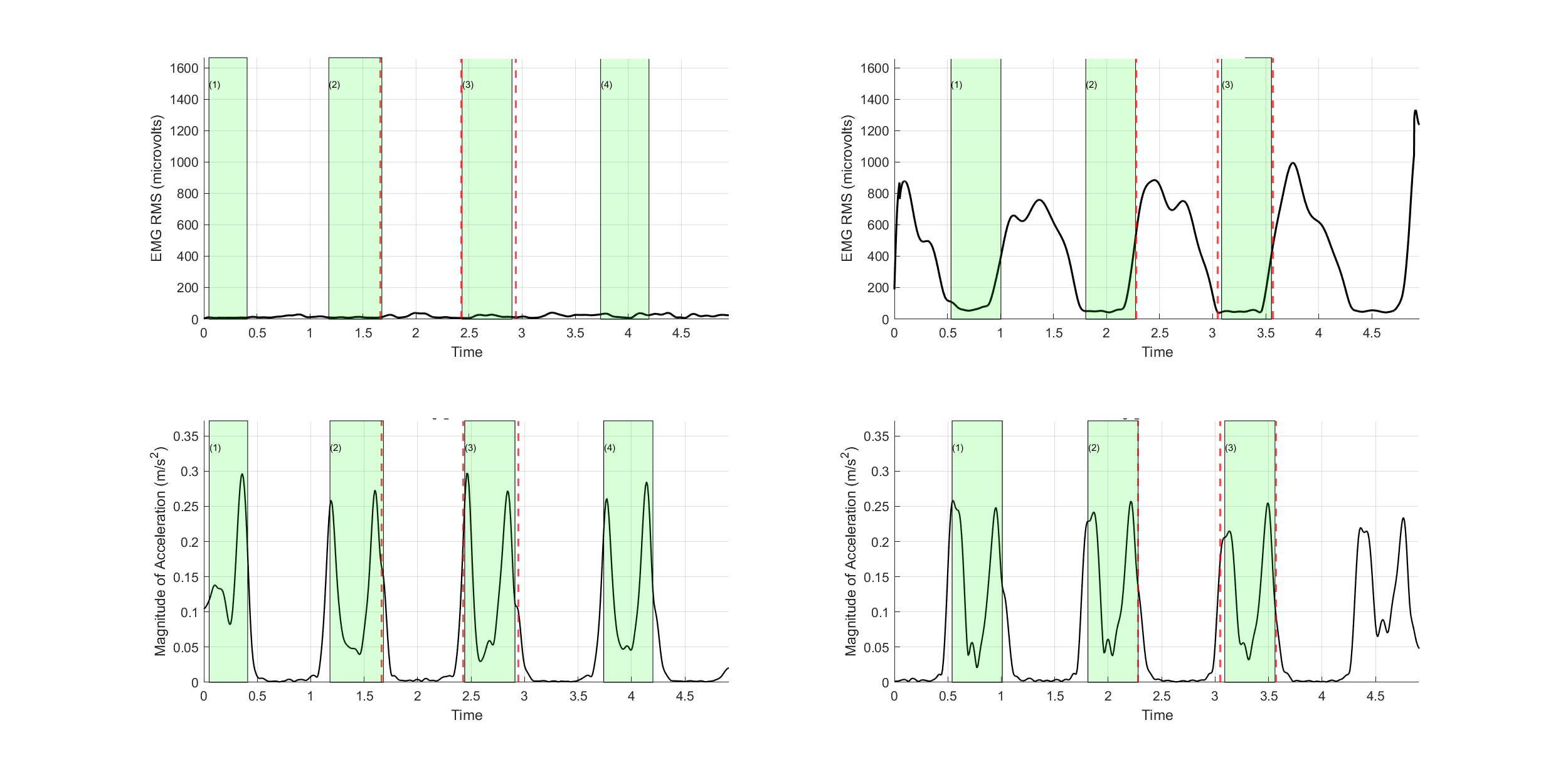

Supplement: Supplementary file 1 [file sensors-22-04957-s001.zip › Part 1 - 3D CGA historic patient data partitions/Figure_12202011.png]

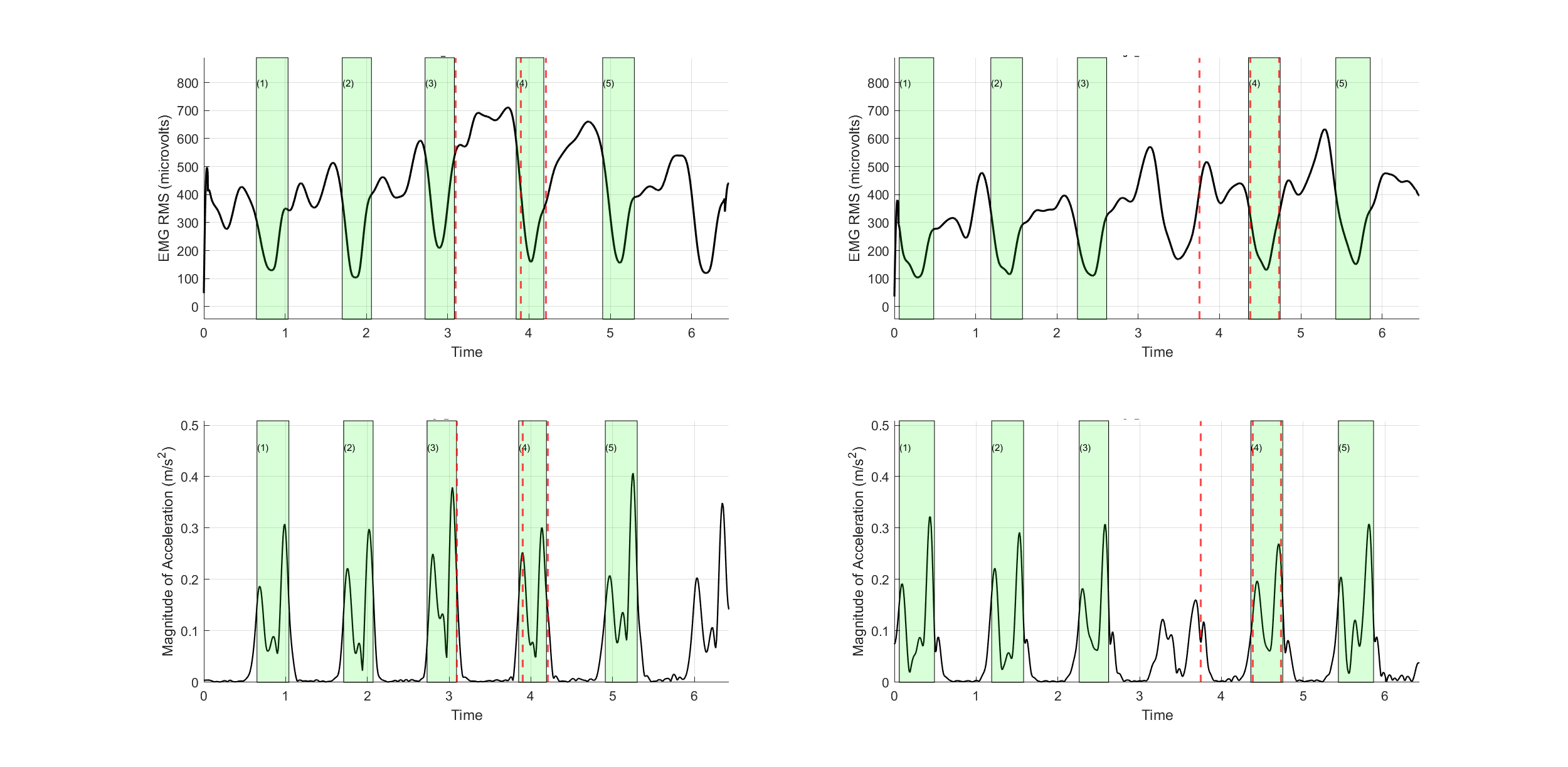

Supplement: Supplementary file 1 [file sensors-22-04957-s001.zip › Part 1 - 3D CGA historic patient data partitions/Figure_12202012.png]

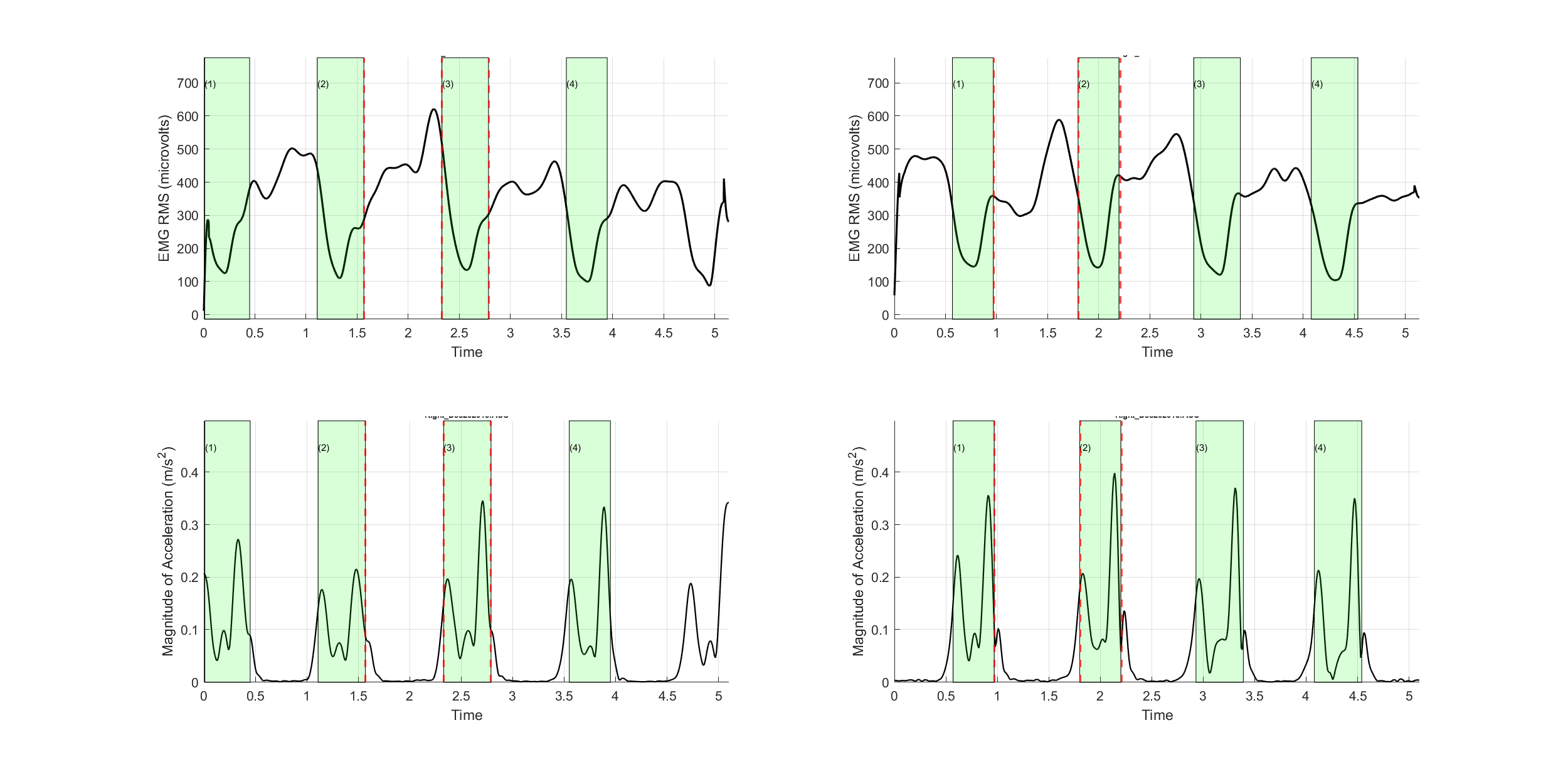

Supplement: Supplementary file 1 [file sensors-22-04957-s001.zip › Part 1 - 3D CGA historic patient data partitions/Figure_12202013.png]

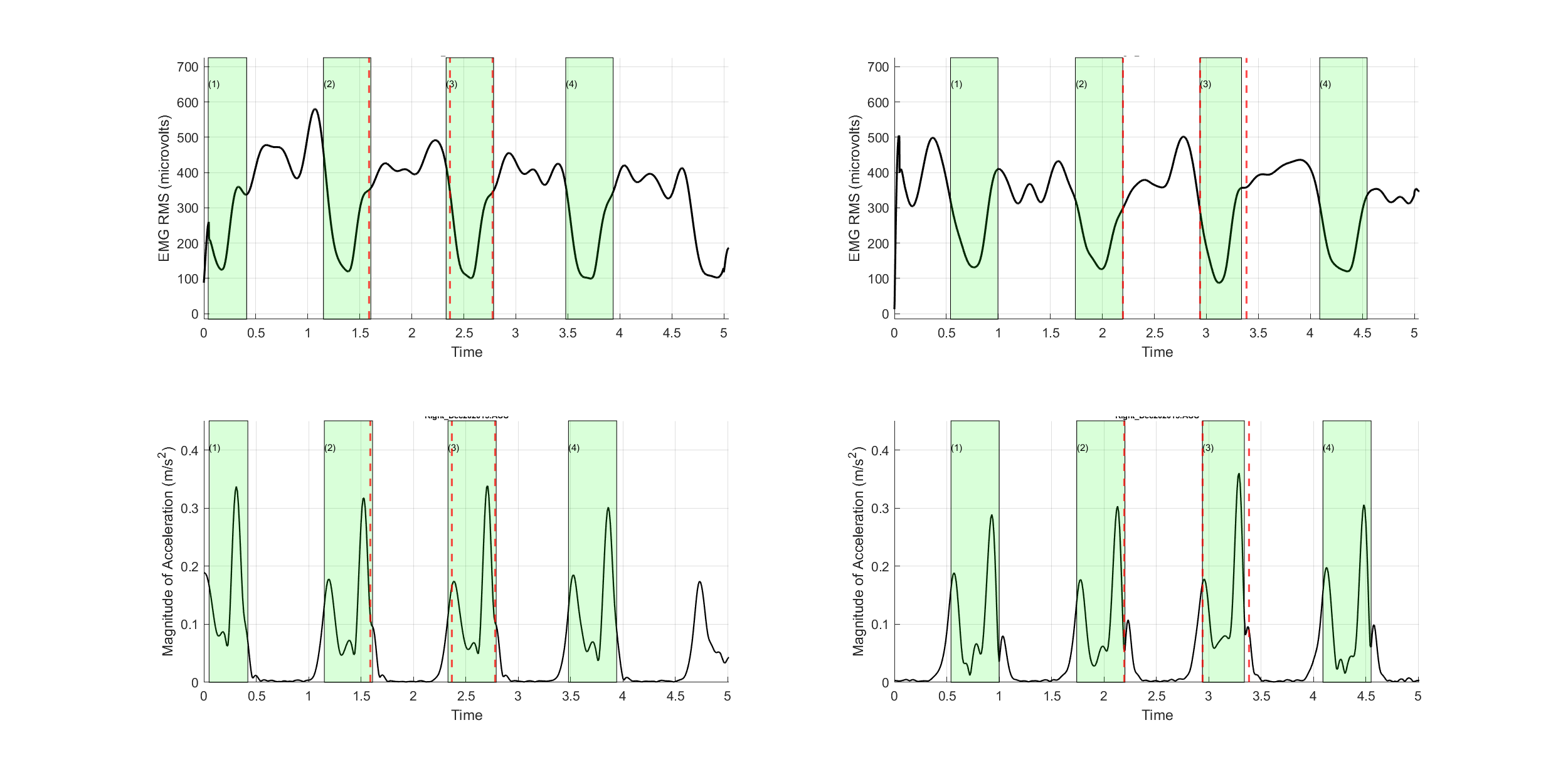

Supplement: Supplementary file 1 [file sensors-22-04957-s001.zip › Part 1 - 3D CGA historic patient data partitions/Figure_12202015.png]

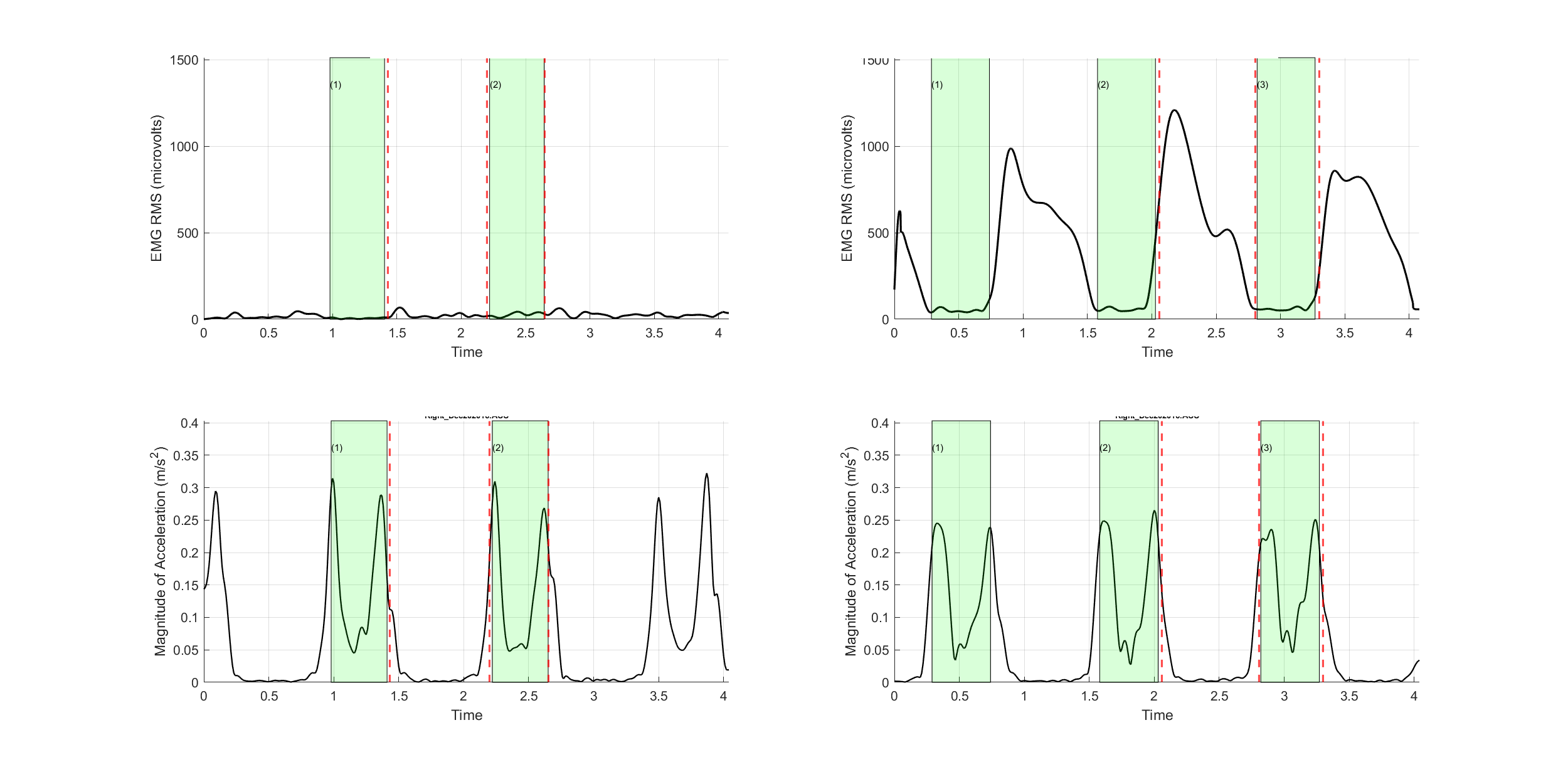

Supplement: Supplementary file 1 [file sensors-22-04957-s001.zip › Part 1 - 3D CGA historic patient data partitions/Figure_12202016.png]

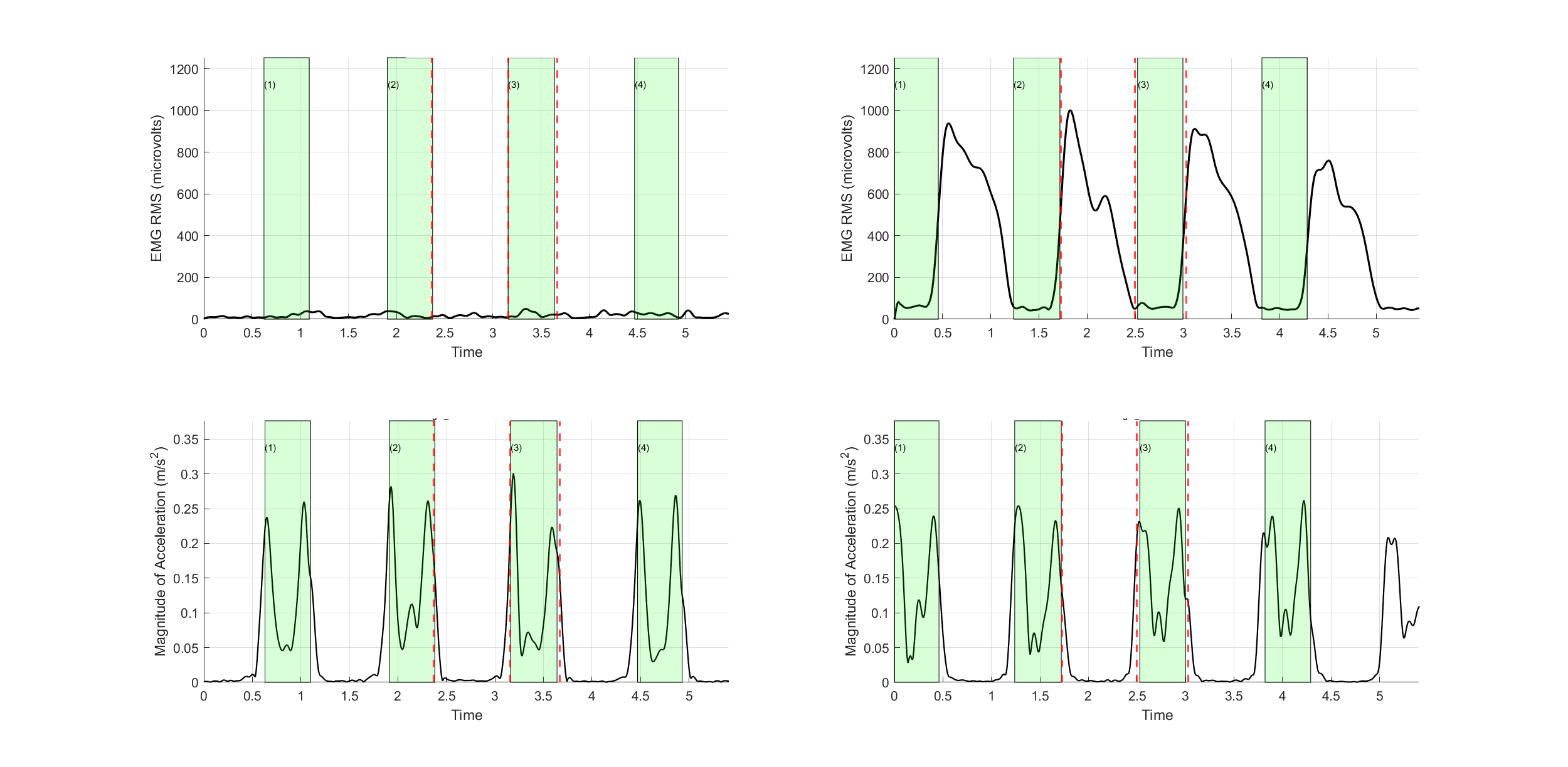

Supplement: Supplementary file 1 [file sensors-22-04957-s001.zip › Part 1 - 3D CGA historic patient data partitions/Figure_12202017.png]

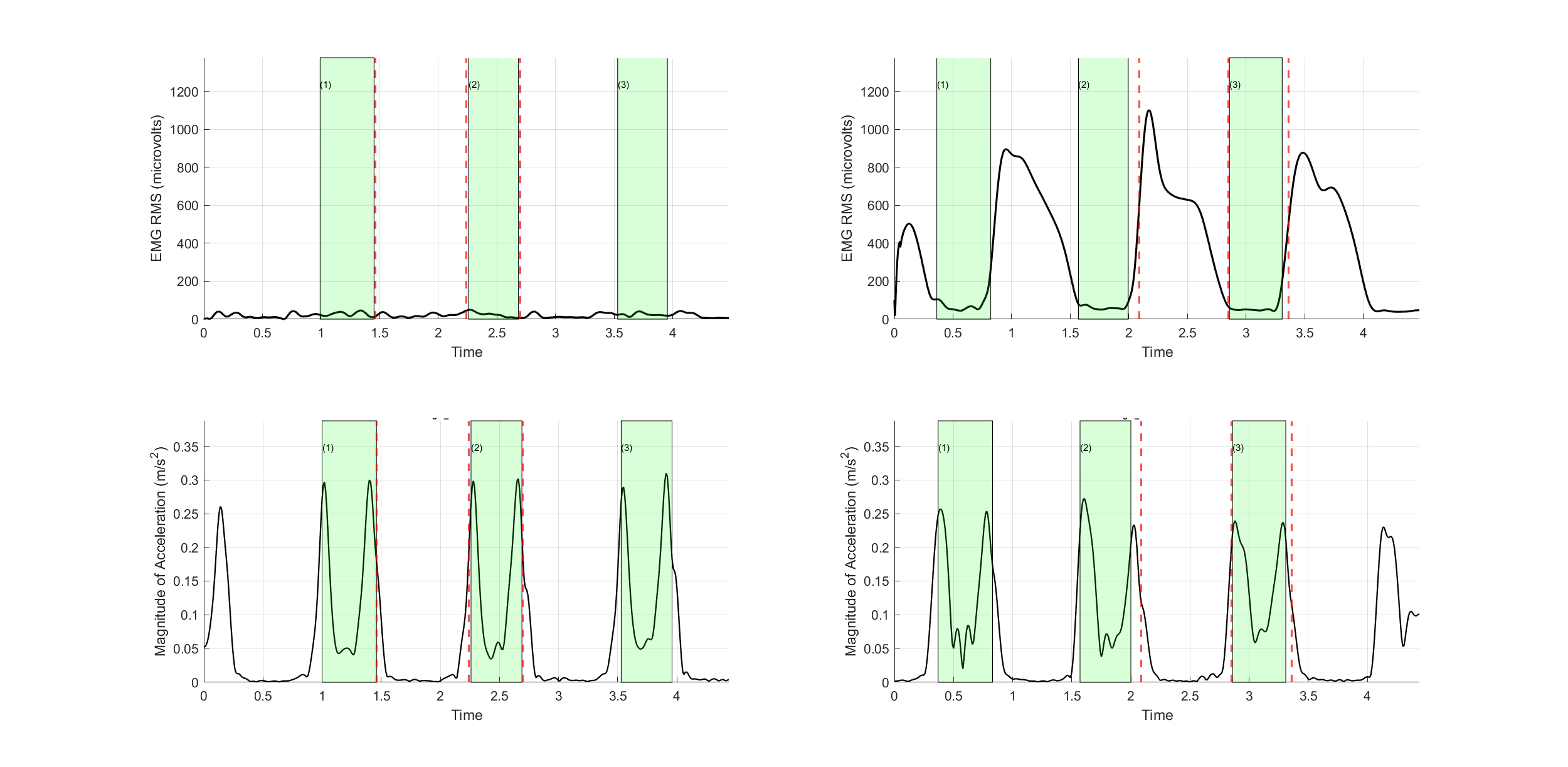

Supplement: Supplementary file 1 [file sensors-22-04957-s001.zip › Part 1 - 3D CGA historic patient data partitions/Figure_12202018.png]

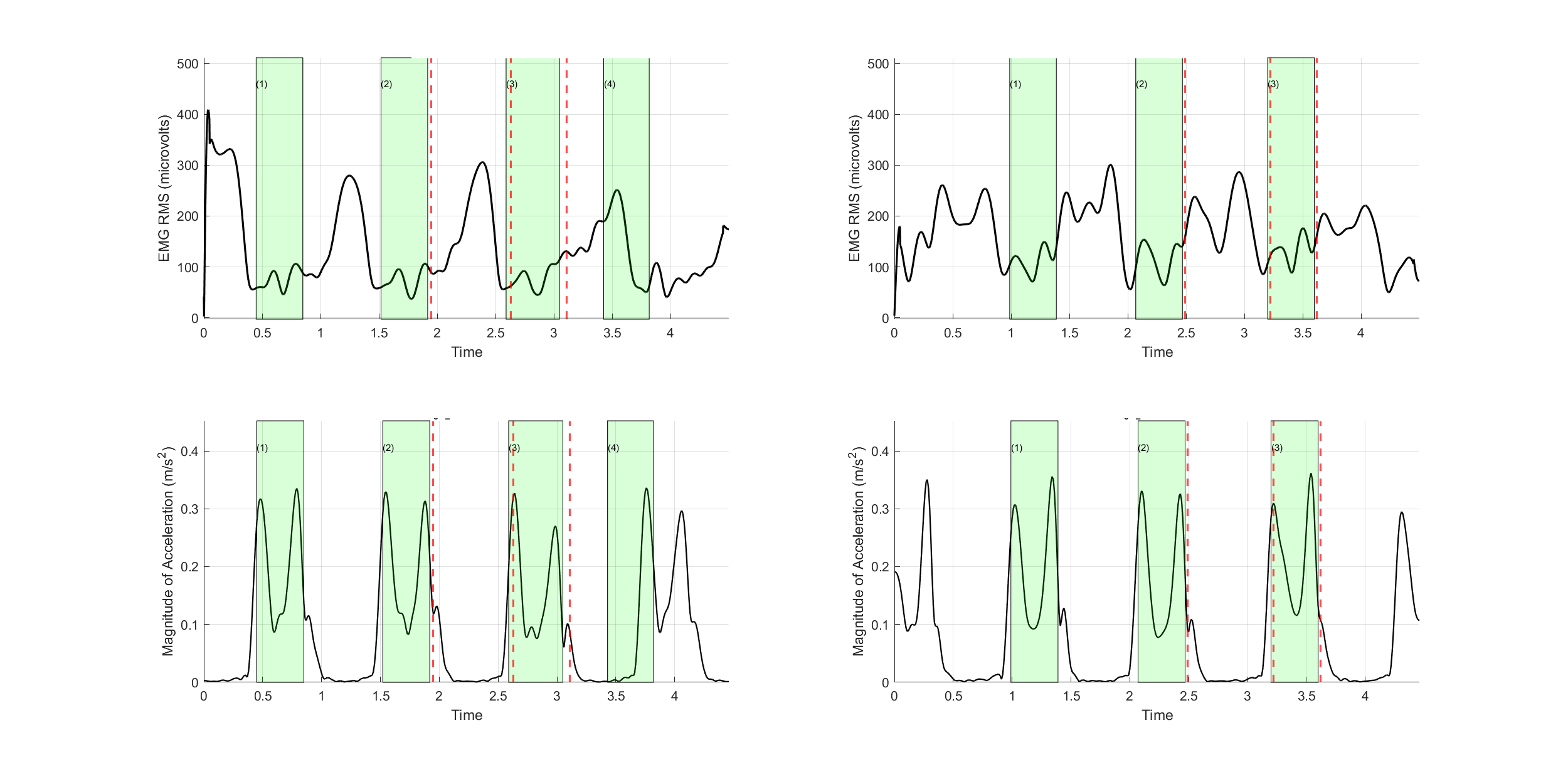

Supplement: Supplementary file 1 [file sensors-22-04957-s001.zip › Part 1 - 3D CGA historic patient data partitions/Figure_3202106.png]

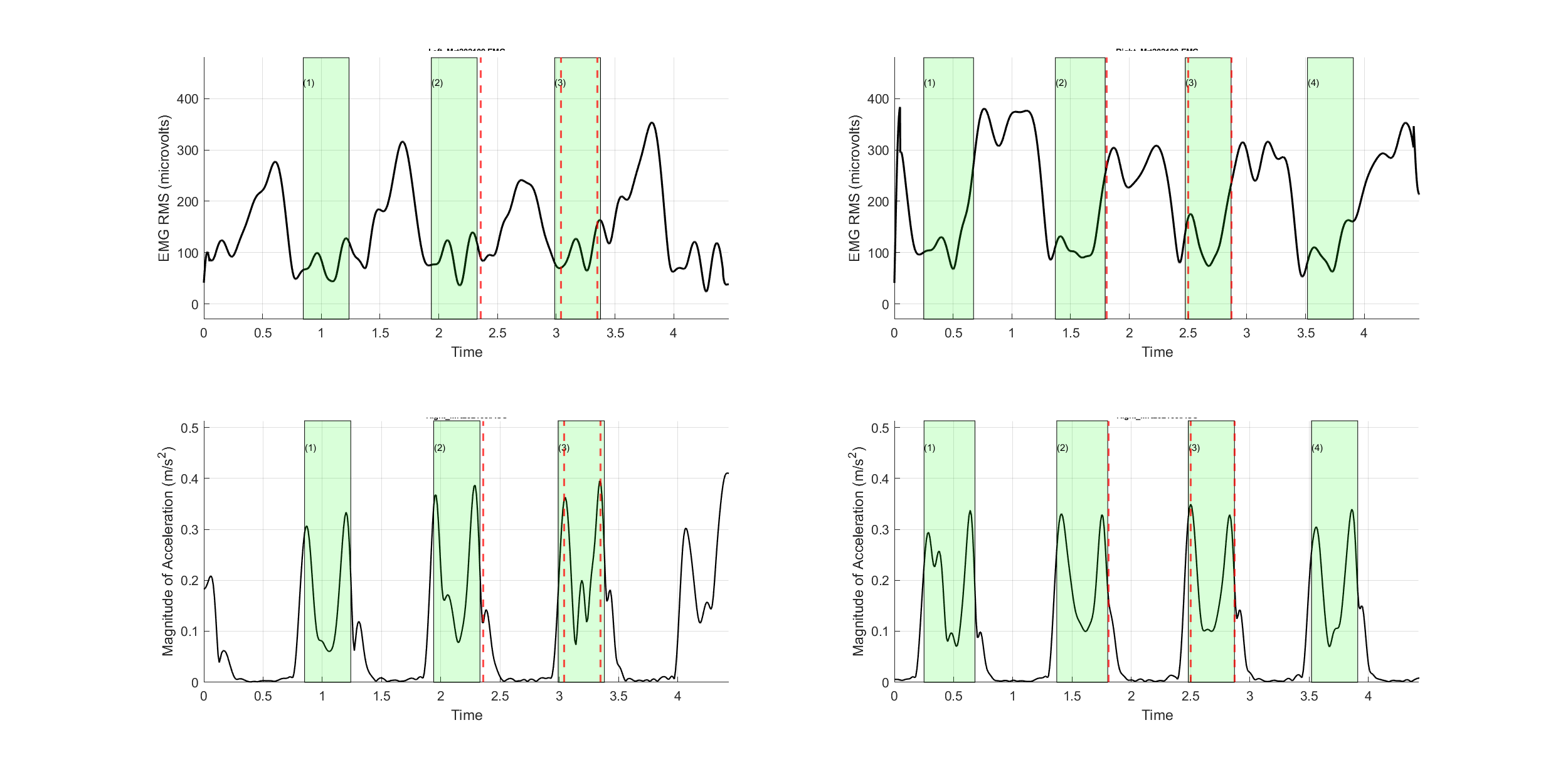

Supplement: Supplementary file 1 [file sensors-22-04957-s001.zip › Part 1 - 3D CGA historic patient data partitions/Figure_3202109.png]

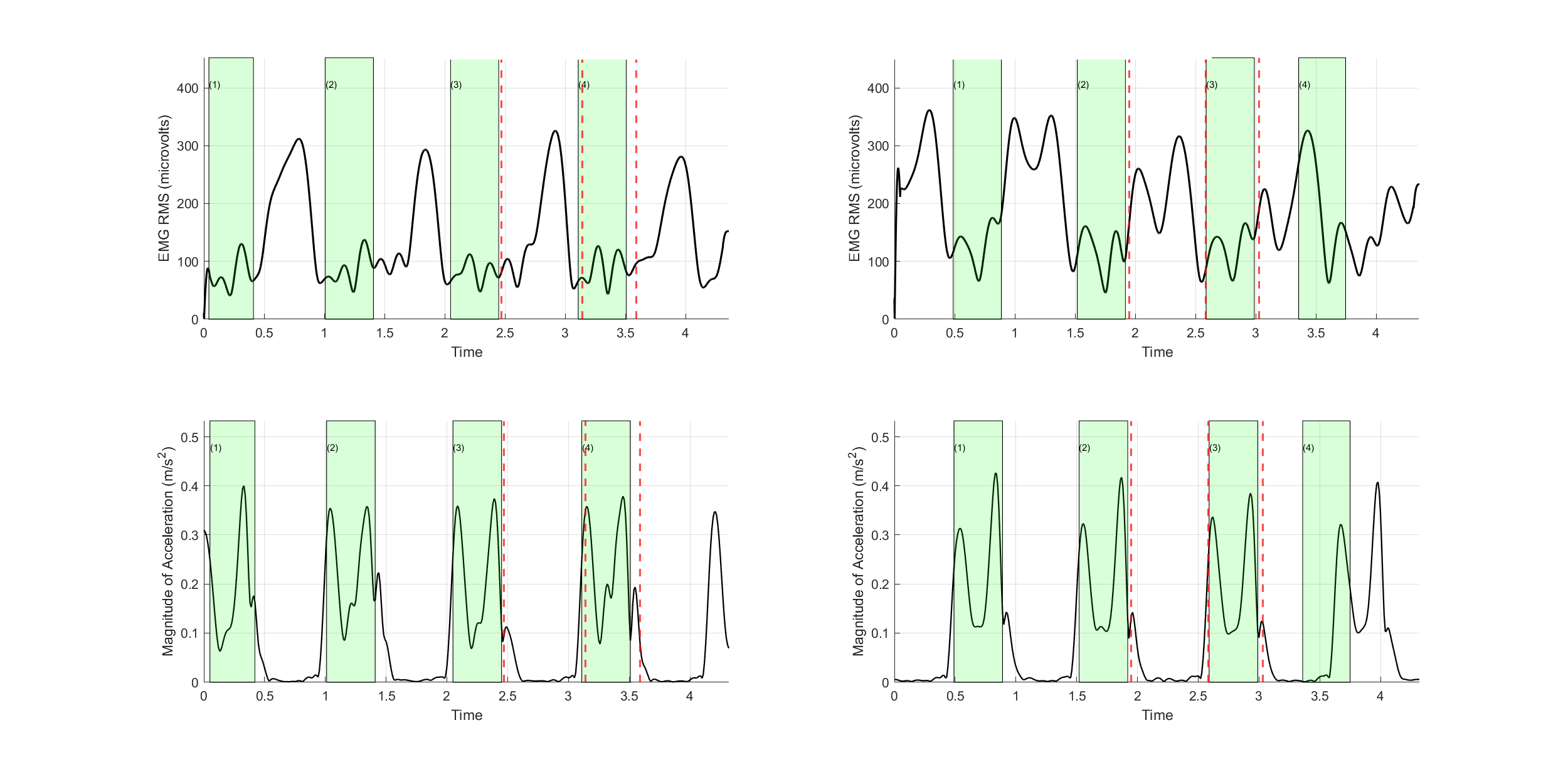

Supplement: Supplementary file 1 [file sensors-22-04957-s001.zip › Part 1 - 3D CGA historic patient data partitions/Figure_3202112.png]

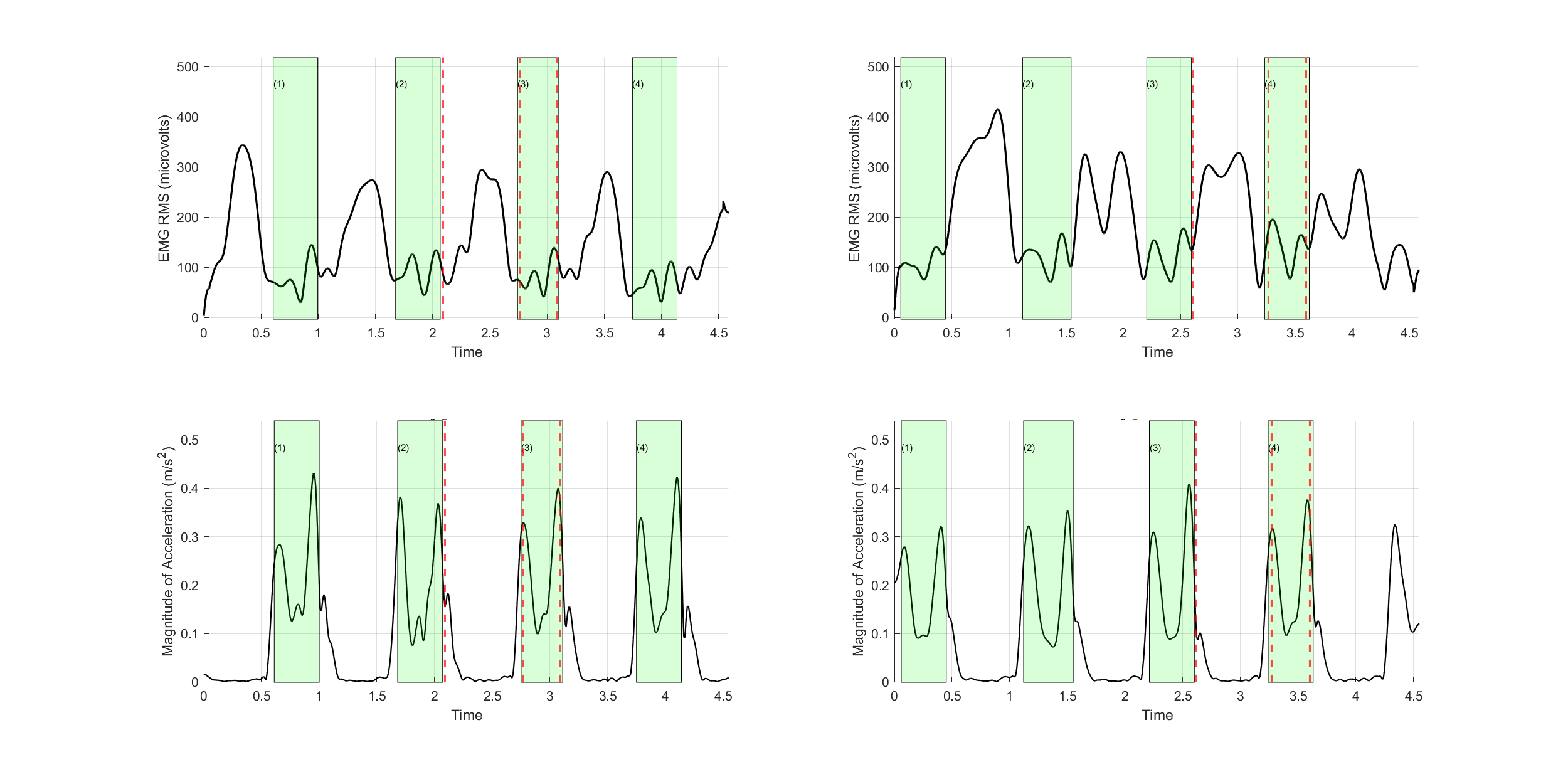

Supplement: Supplementary file 1 [file sensors-22-04957-s001.zip › Part 1 - 3D CGA historic patient data partitions/Figure_3202113-1.png]

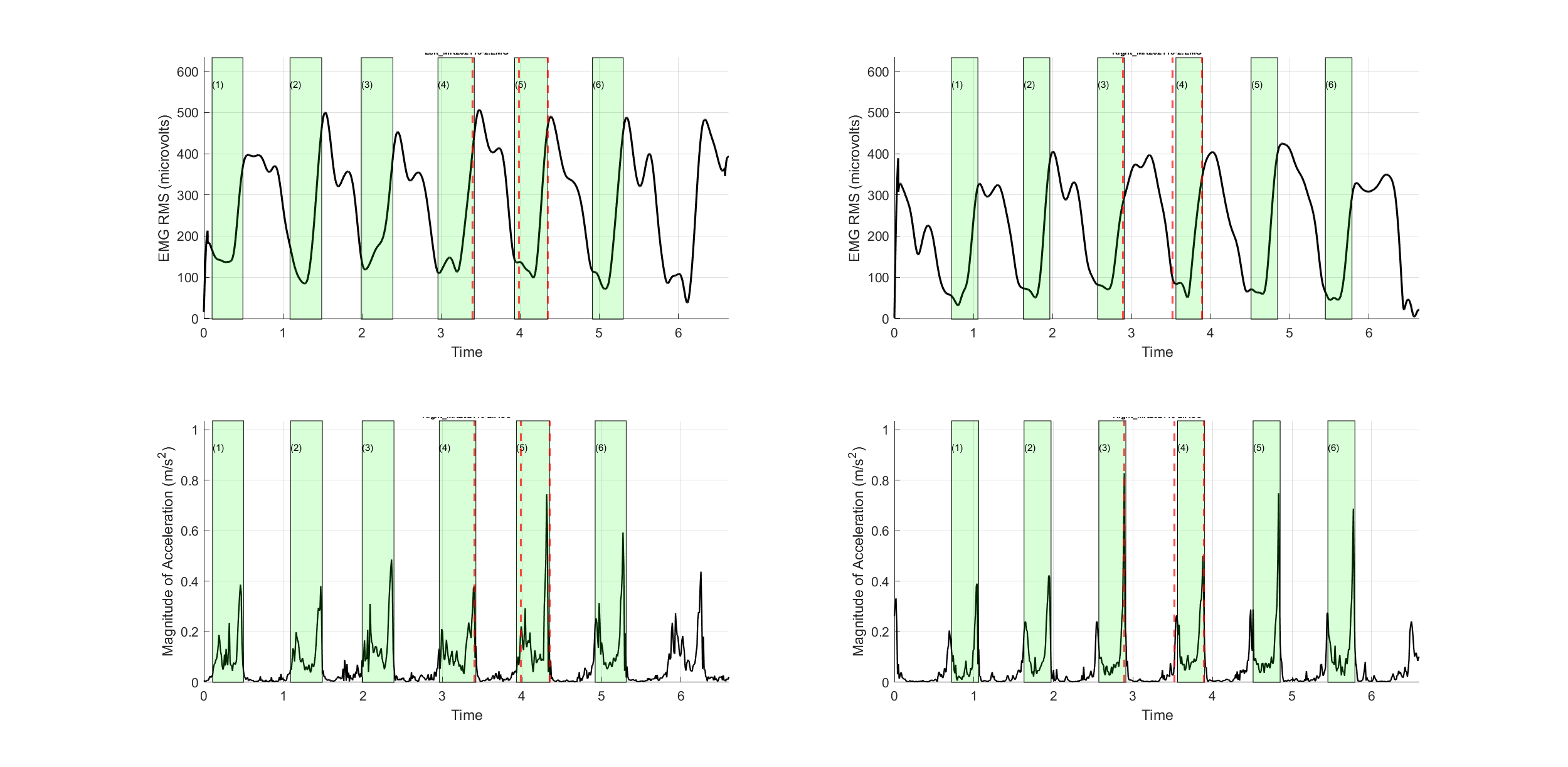

Supplement: Supplementary file 1 [file sensors-22-04957-s001.zip › Part 1 - 3D CGA historic patient data partitions/Figure_3202113-2.png]

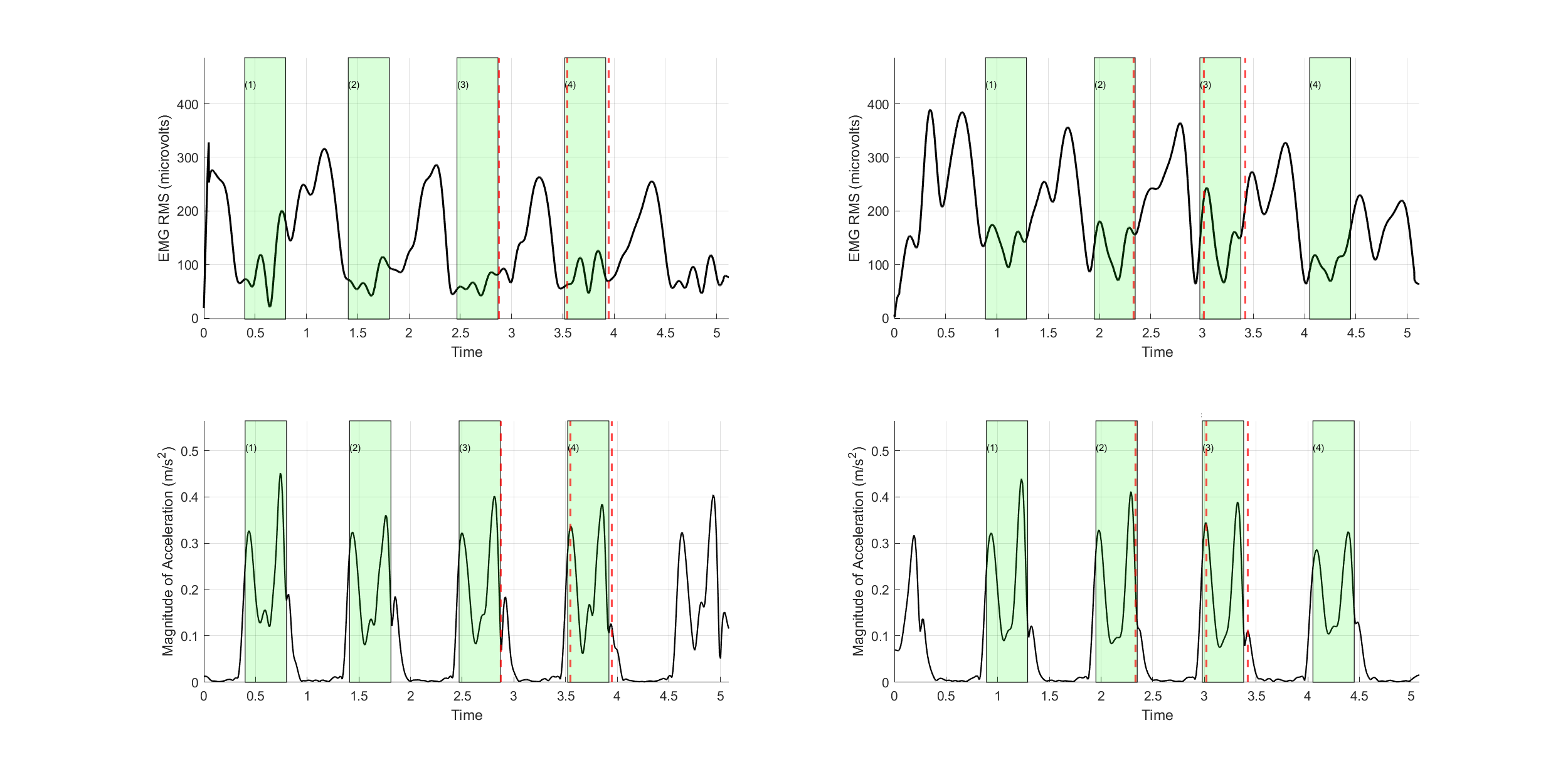

Supplement: Supplementary file 1 [file sensors-22-04957-s001.zip › Part 1 - 3D CGA historic patient data partitions/Figure_3202114-1.png]

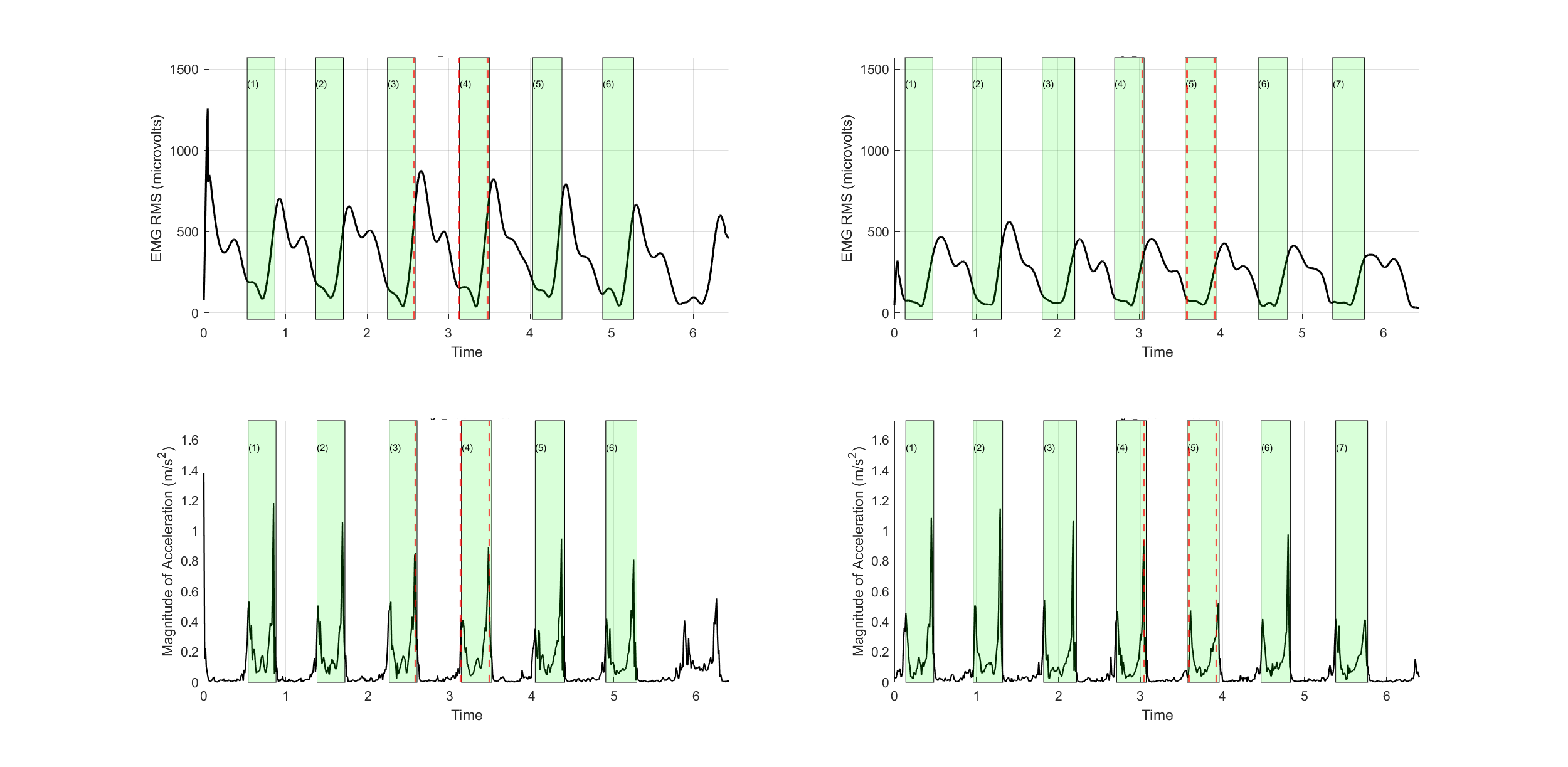

Supplement: Supplementary file 1 [file sensors-22-04957-s001.zip › Part 1 - 3D CGA historic patient data partitions/Figure_3202114-2.png]

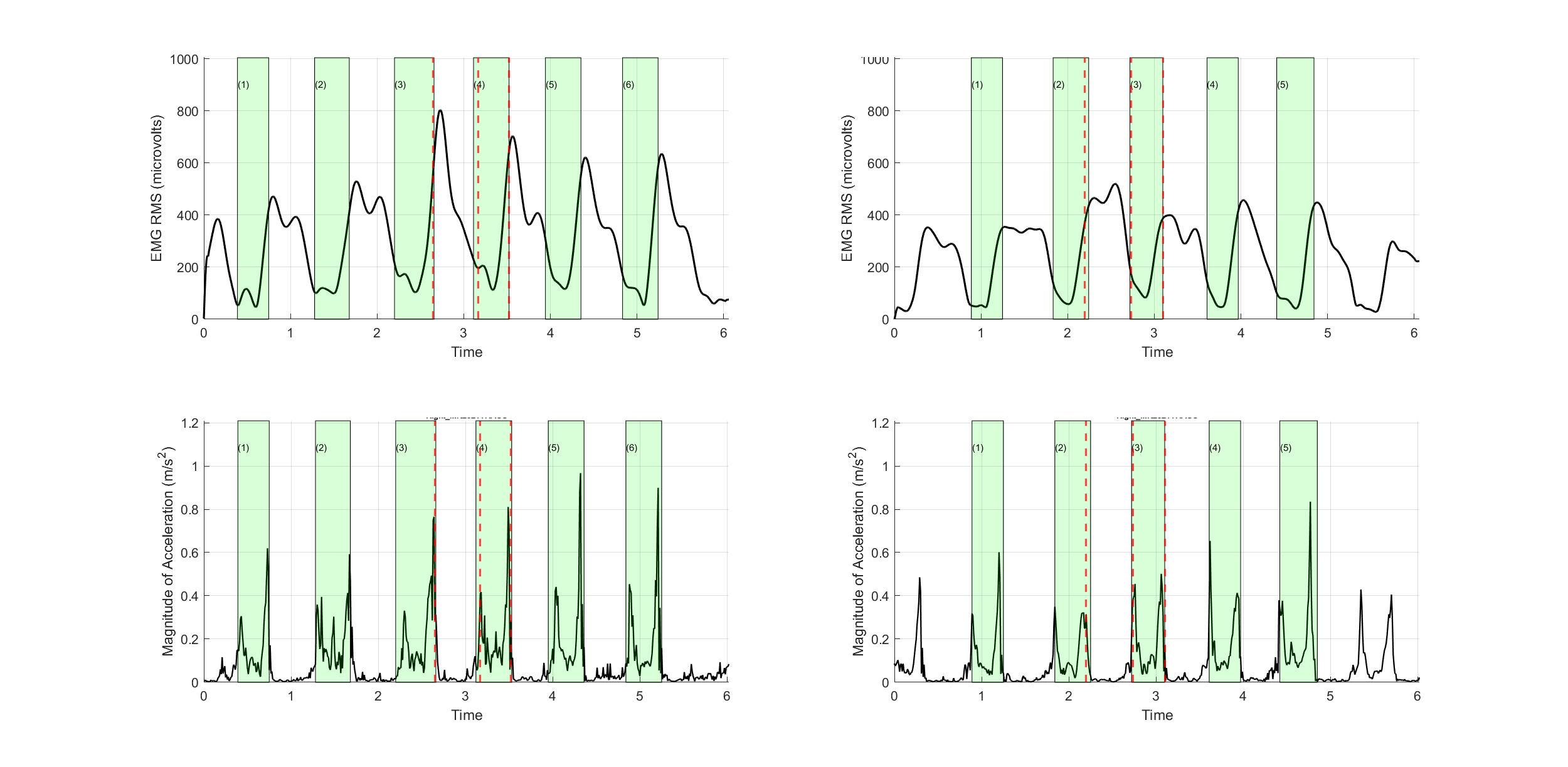

Supplement: Supplementary file 1 [file sensors-22-04957-s001.zip › Part 1 - 3D CGA historic patient data partitions/Figure_3202117.png]

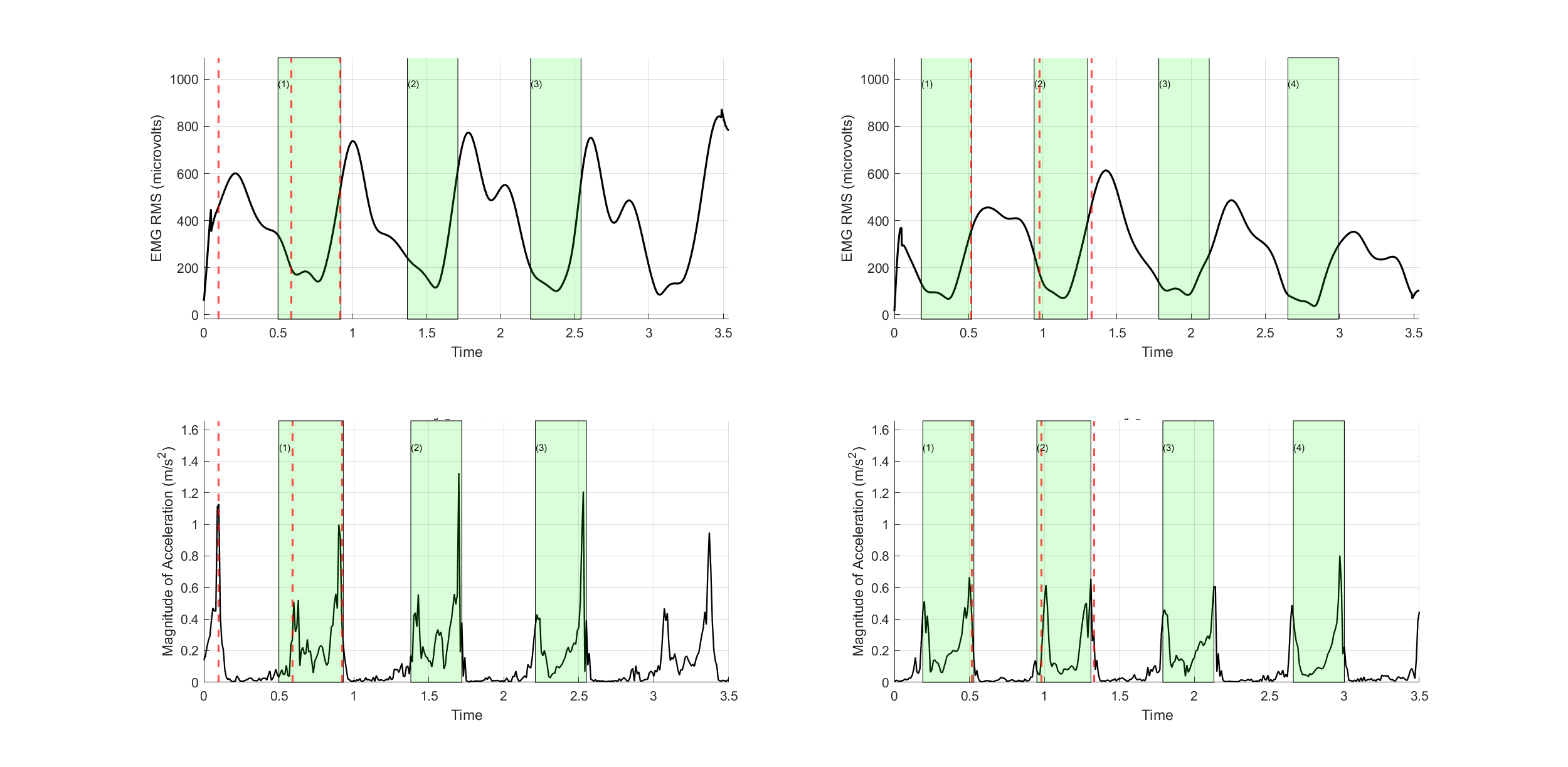

Supplement: Supplementary file 1 [file sensors-22-04957-s001.zip › Part 1 - 3D CGA historic patient data partitions/Figure_3202118.png]

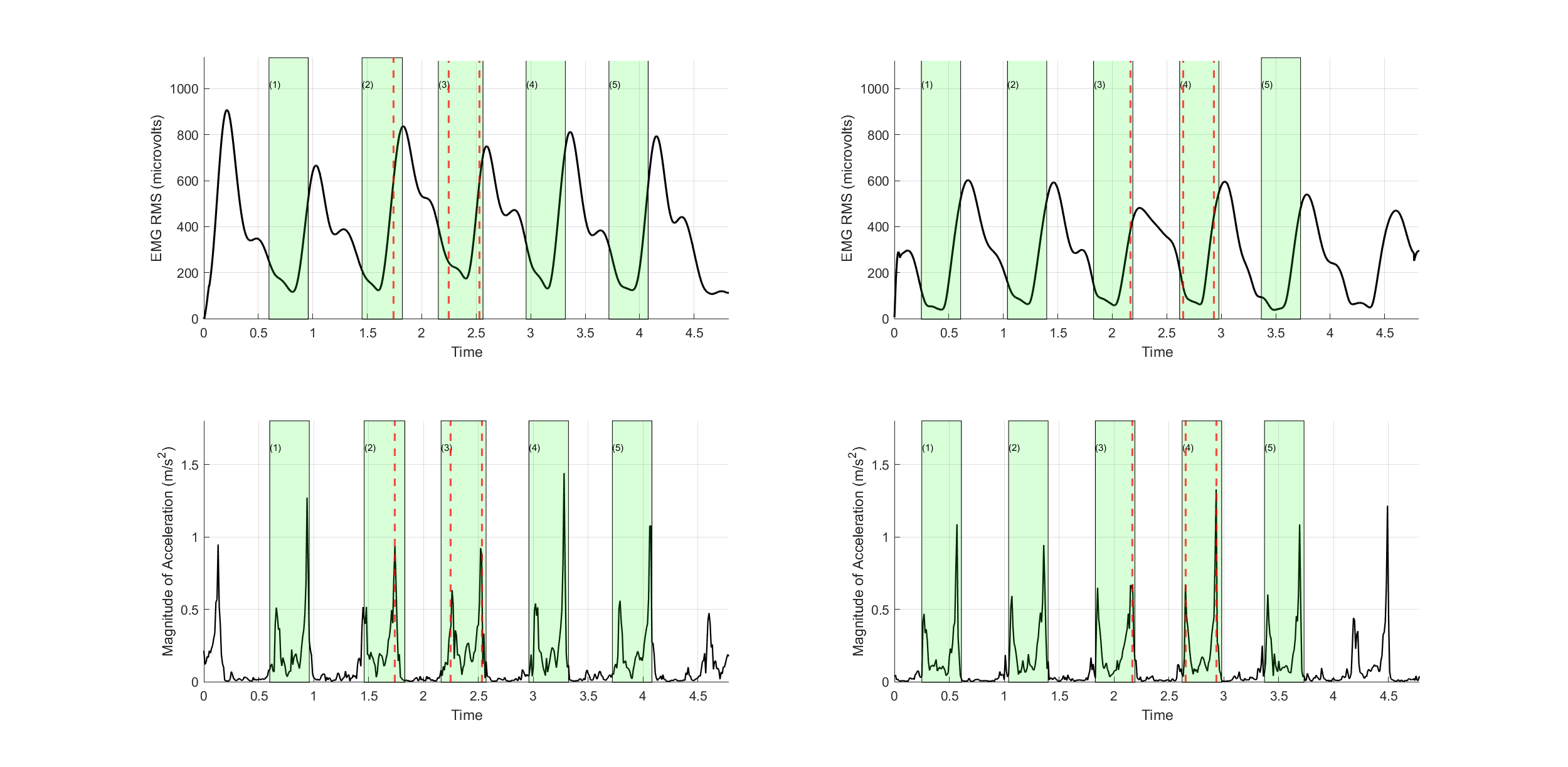

Supplement: Supplementary file 1 [file sensors-22-04957-s001.zip › Part 1 - 3D CGA historic patient data partitions/Figure_3202119.png]

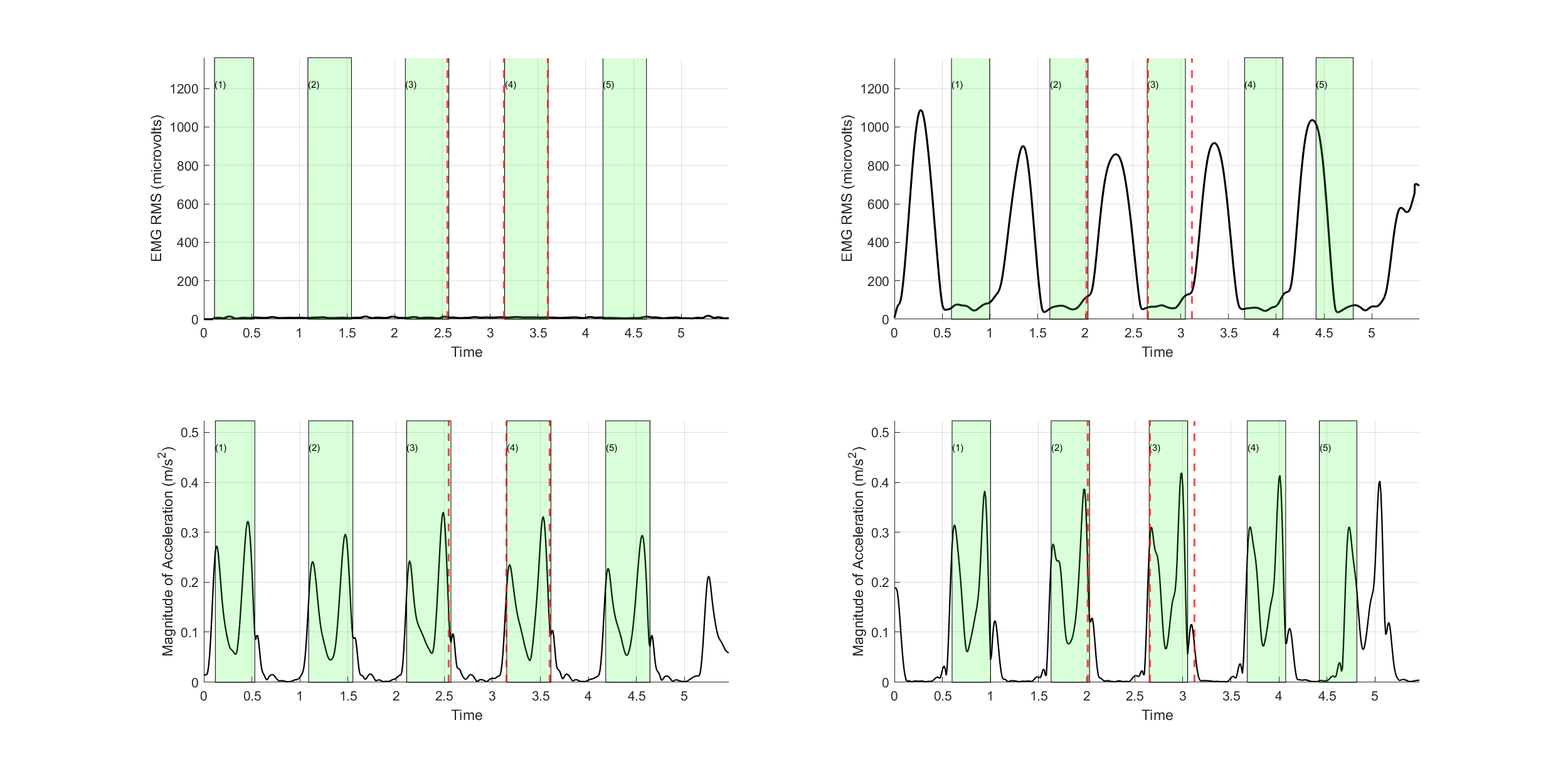

Supplement: Supplementary file 1 [file sensors-22-04957-s001.zip › Part 1 - 3D CGA historic patient data partitions/Figure_4202107.png]

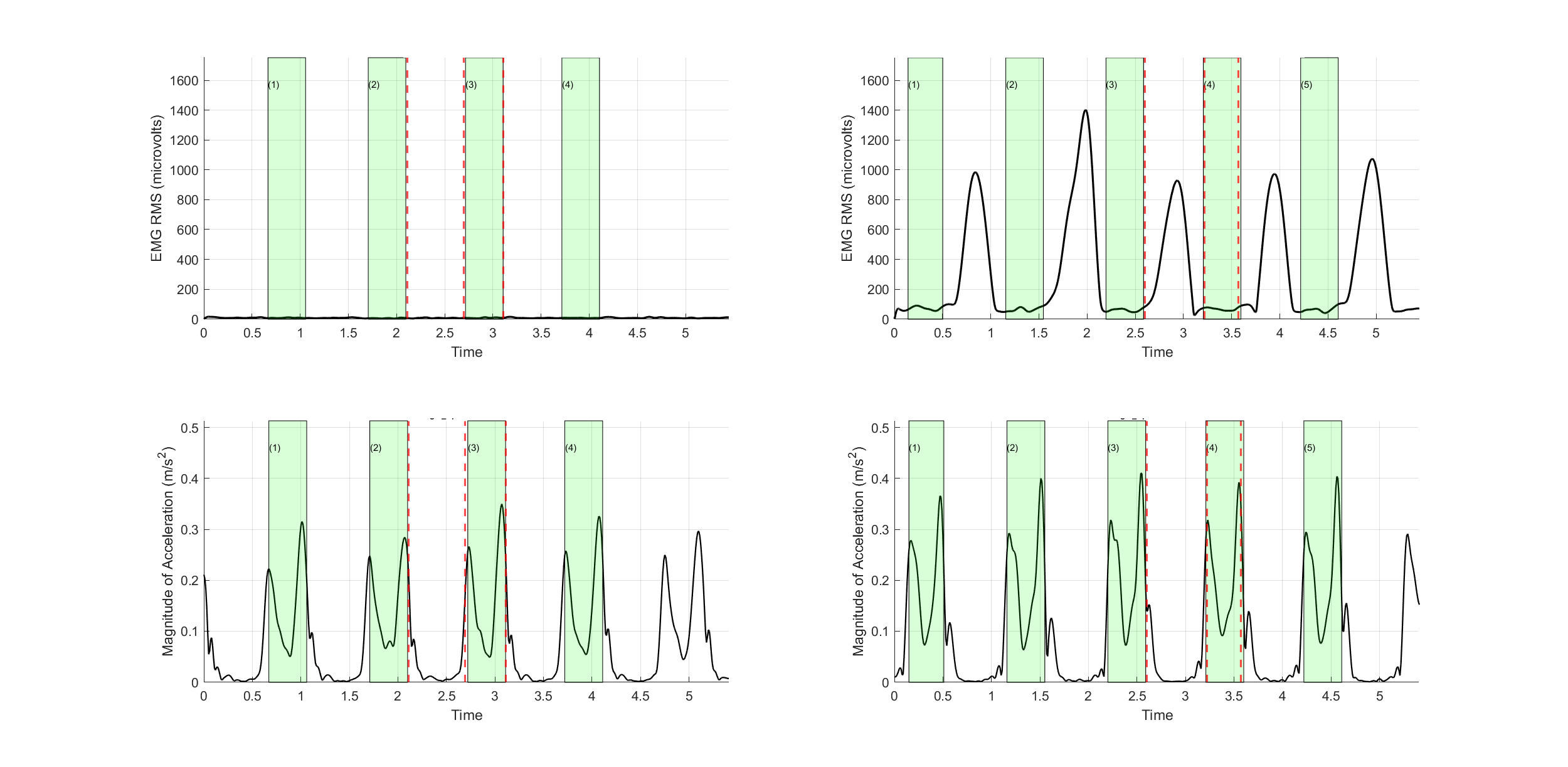

Supplement: Supplementary file 1 [file sensors-22-04957-s001.zip › Part 1 - 3D CGA historic patient data partitions/Figure_4202108-1.png]

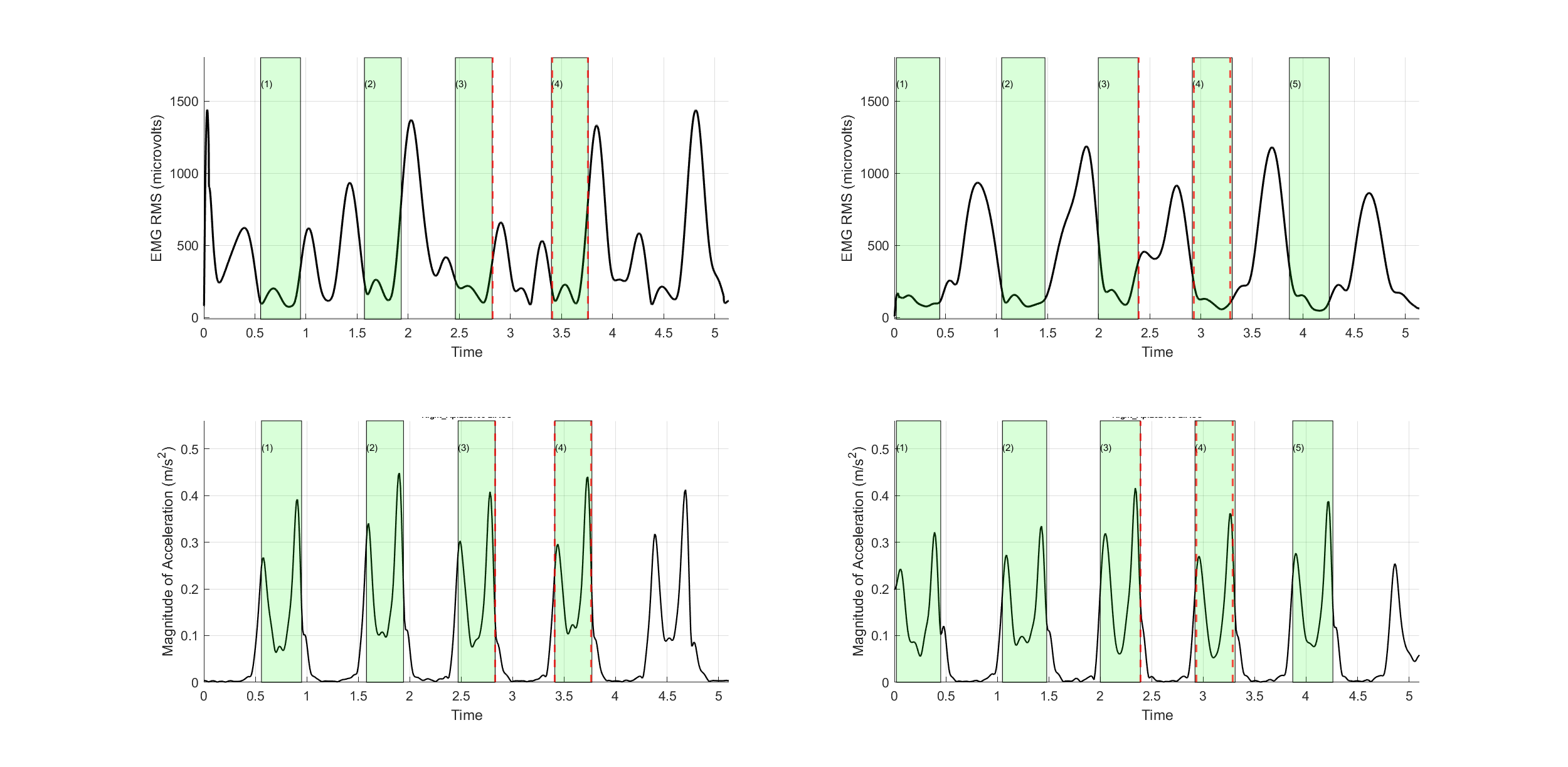

Supplement: Supplementary file 1 [file sensors-22-04957-s001.zip › Part 1 - 3D CGA historic patient data partitions/Figure_4202108-2.png]

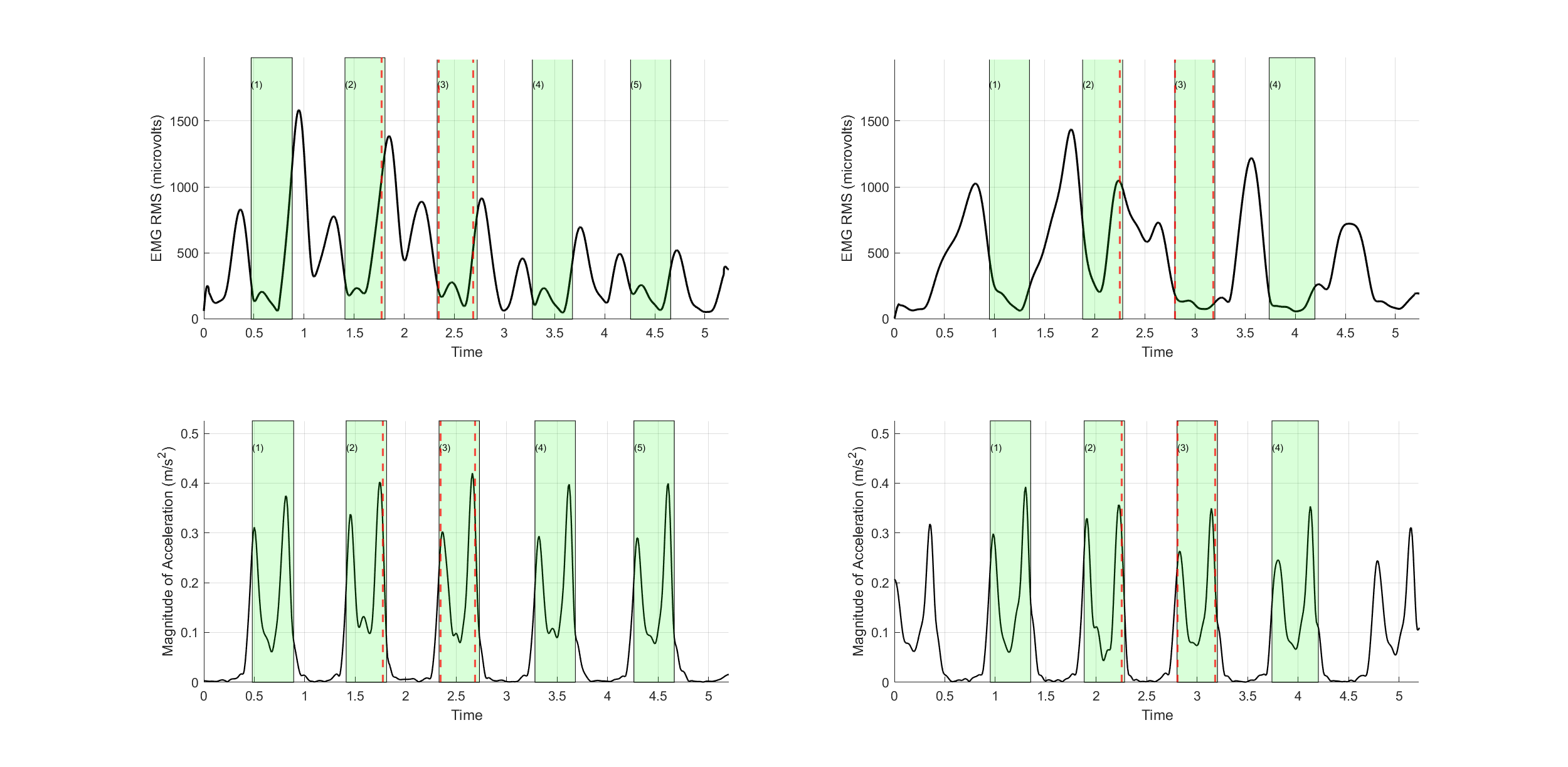

Supplement: Supplementary file 1 [file sensors-22-04957-s001.zip › Part 1 - 3D CGA historic patient data partitions/Figure_4202109.png]

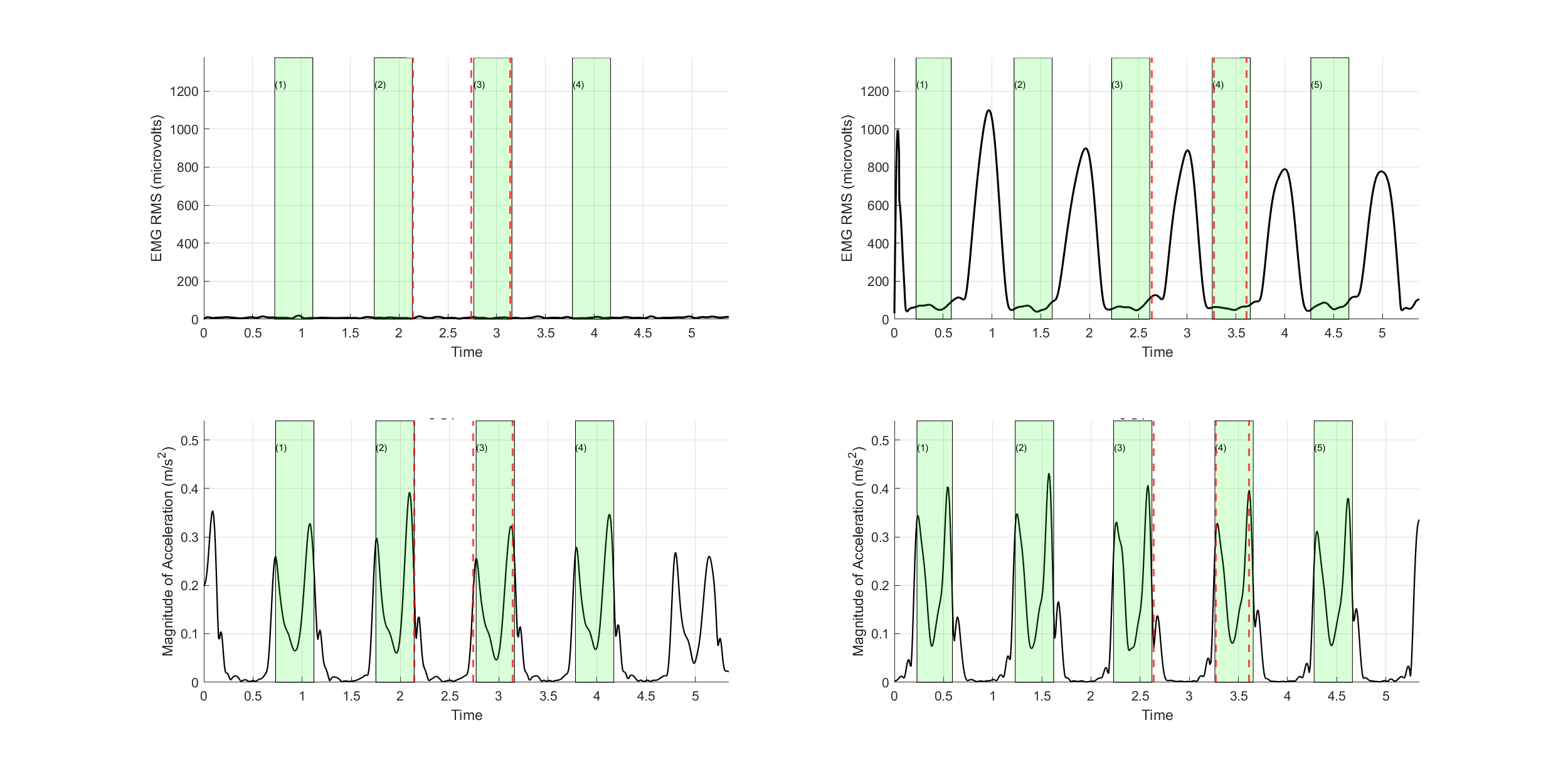

Supplement: Supplementary file 1 [file sensors-22-04957-s001.zip › Part 1 - 3D CGA historic patient data partitions/Figure_4202110-1.png]

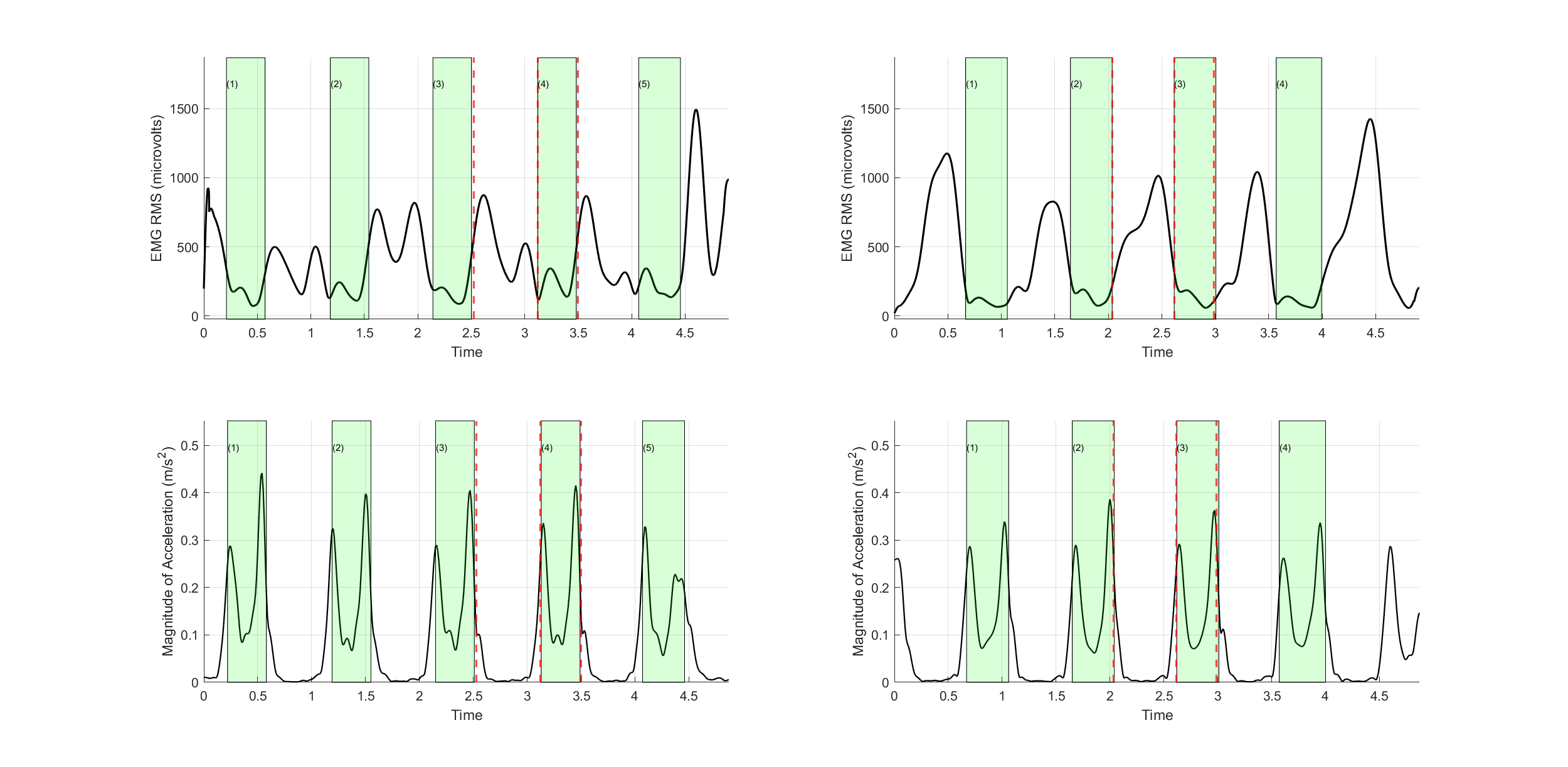

Supplement: Supplementary file 1 [file sensors-22-04957-s001.zip › Part 1 - 3D CGA historic patient data partitions/Figure_4202110-2.png]

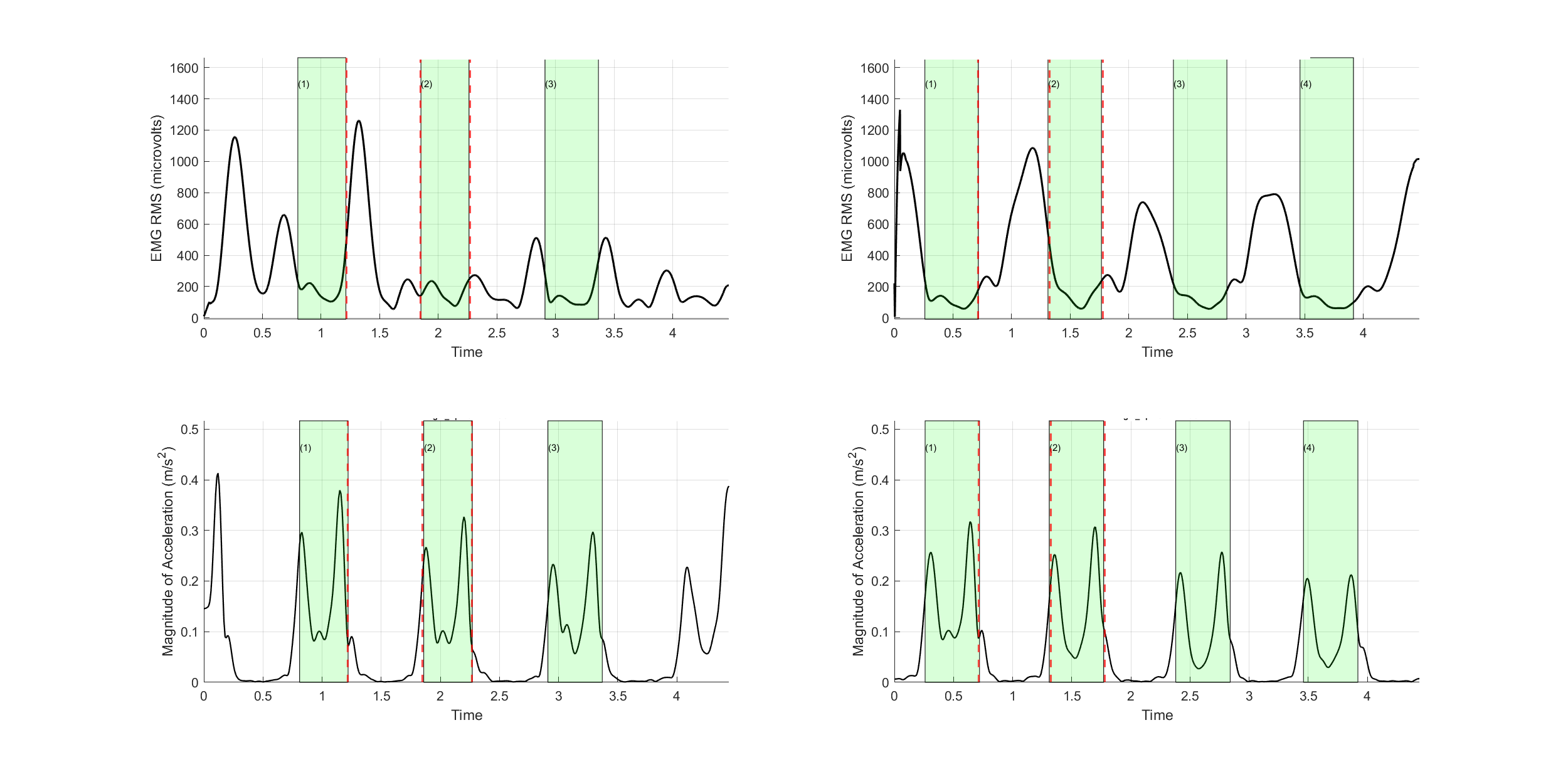

Supplement: Supplementary file 1 [file sensors-22-04957-s001.zip › Part 1 - 3D CGA historic patient data partitions/Figure_4202111.png]

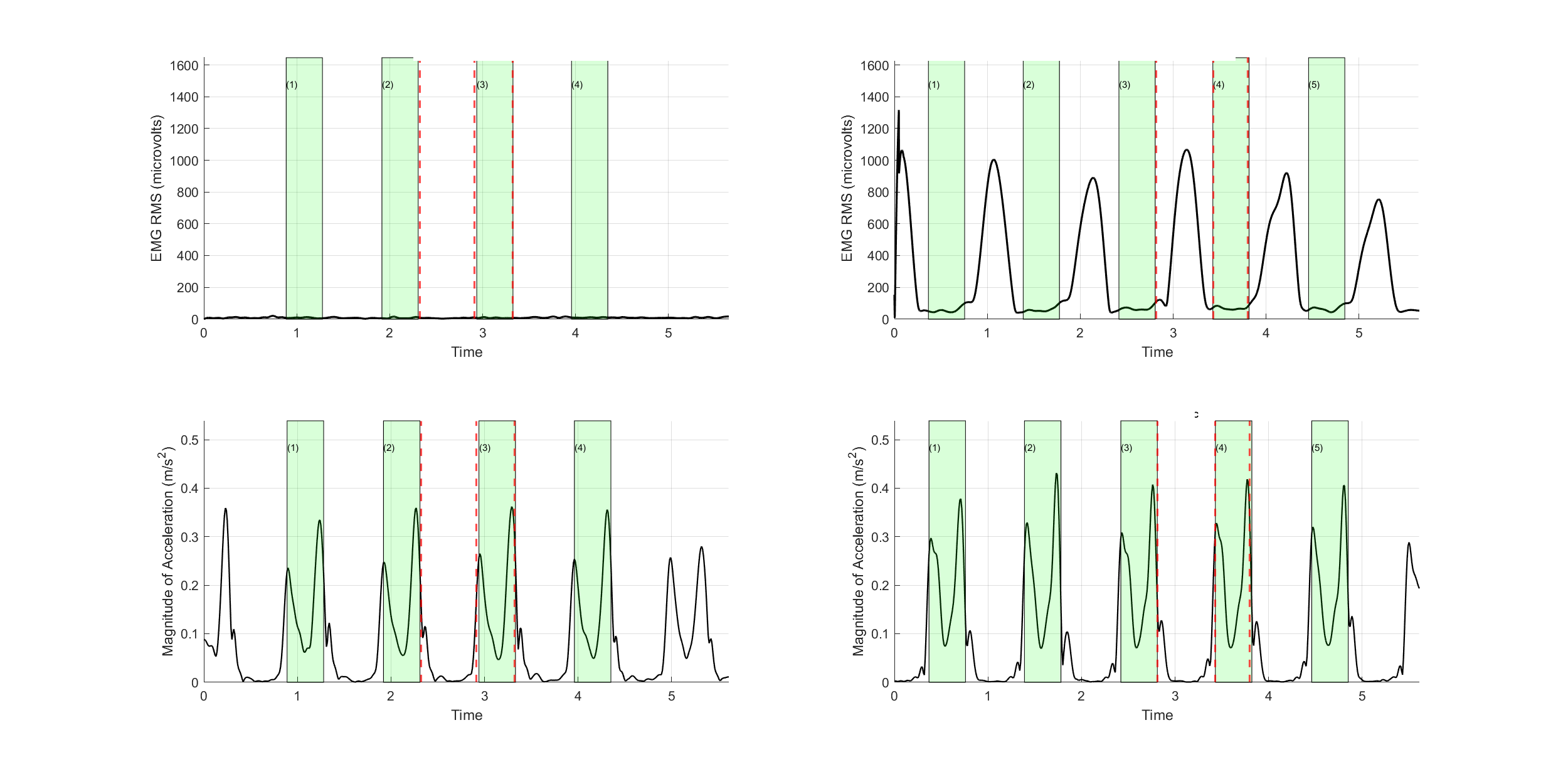

Supplement: Supplementary file 1 [file sensors-22-04957-s001.zip › Part 1 - 3D CGA historic patient data partitions/Figure_4202112.png]

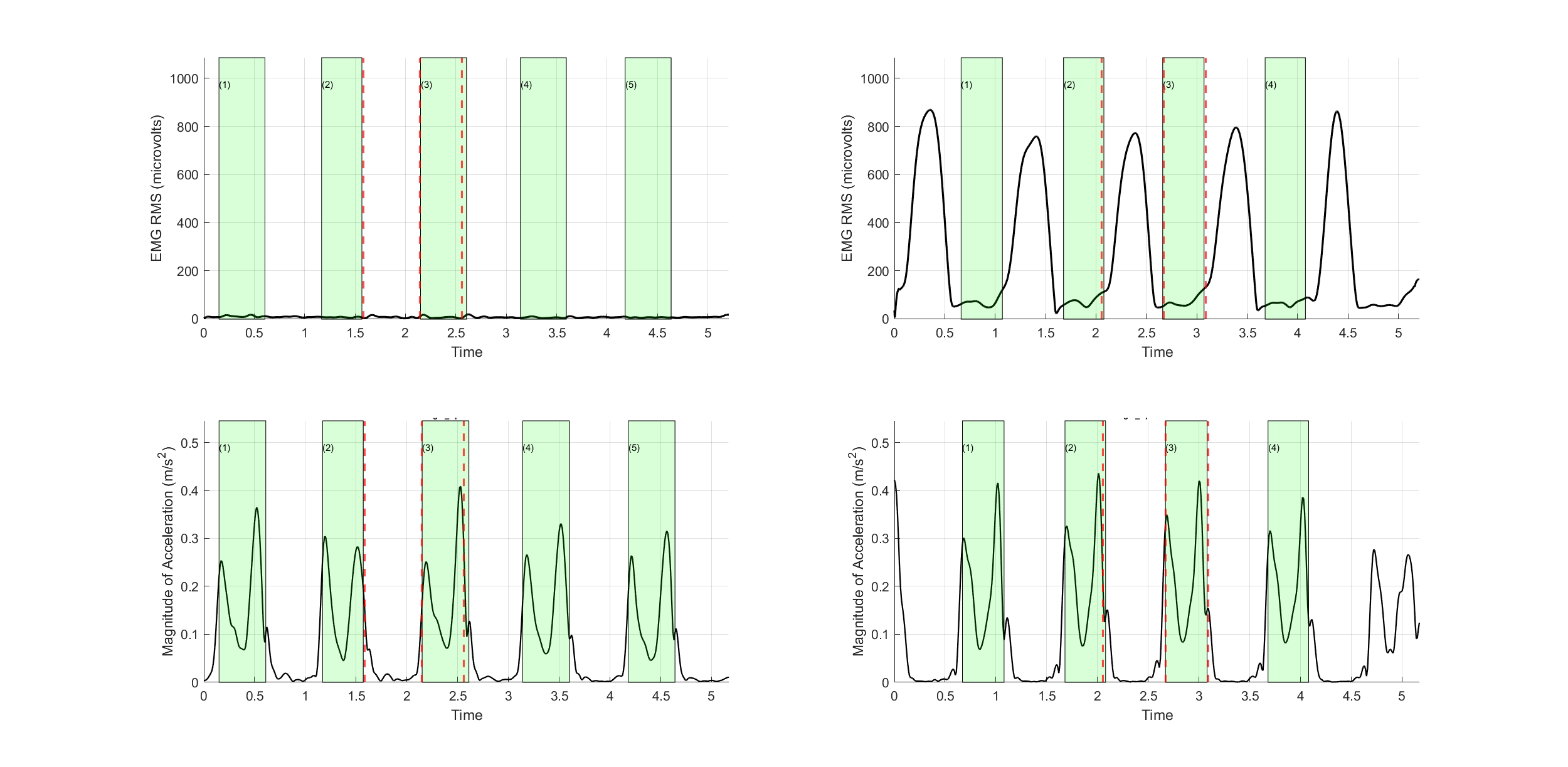

Supplement: Supplementary file 1 [file sensors-22-04957-s001.zip › Part 1 - 3D CGA historic patient data partitions/Figure_4202113.png]

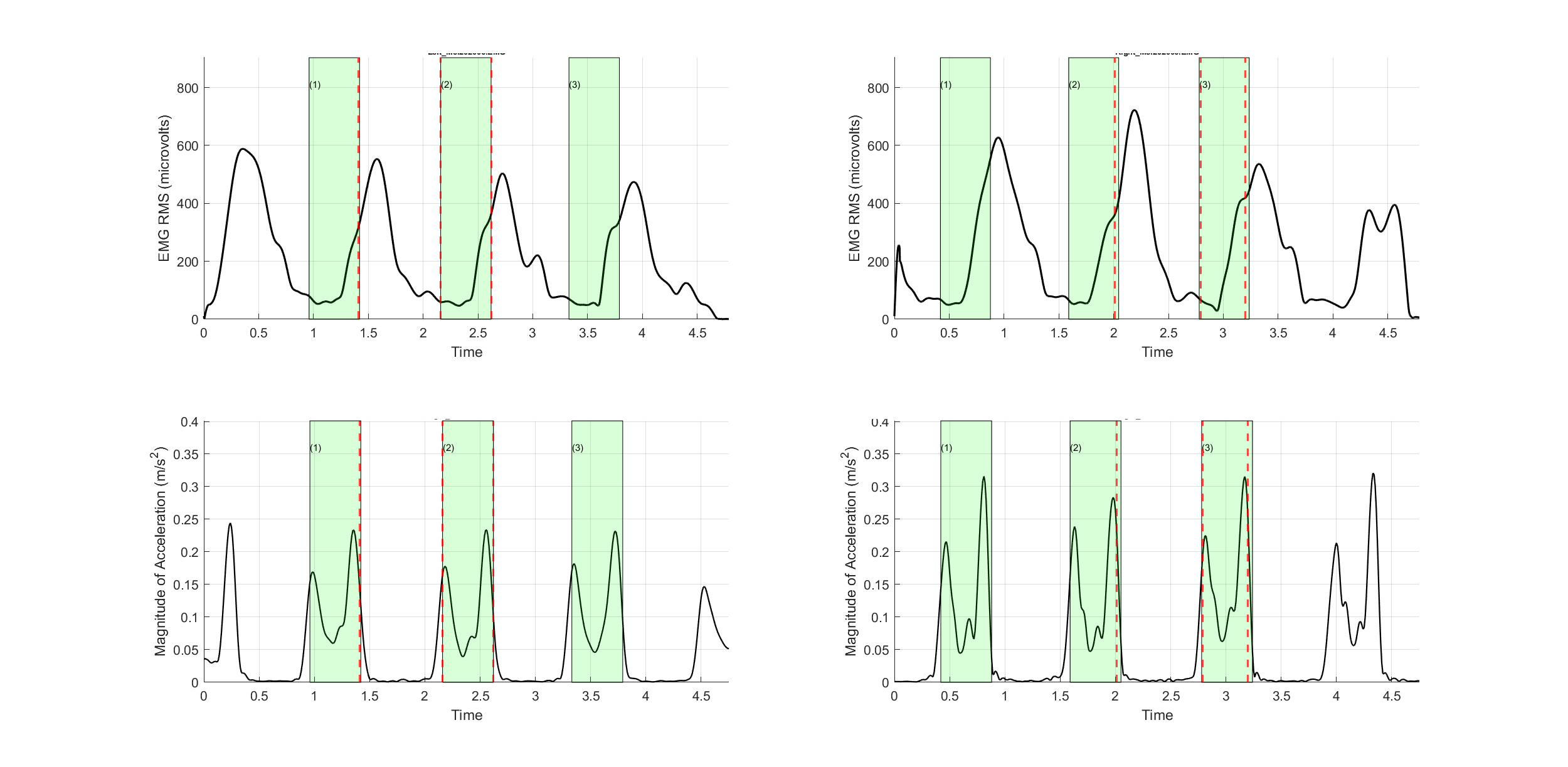

Supplement: Supplementary file 1 [file sensors-22-04957-s001.zip › Part 1 - 3D CGA historic patient data partitions/Figure_5202006.png]

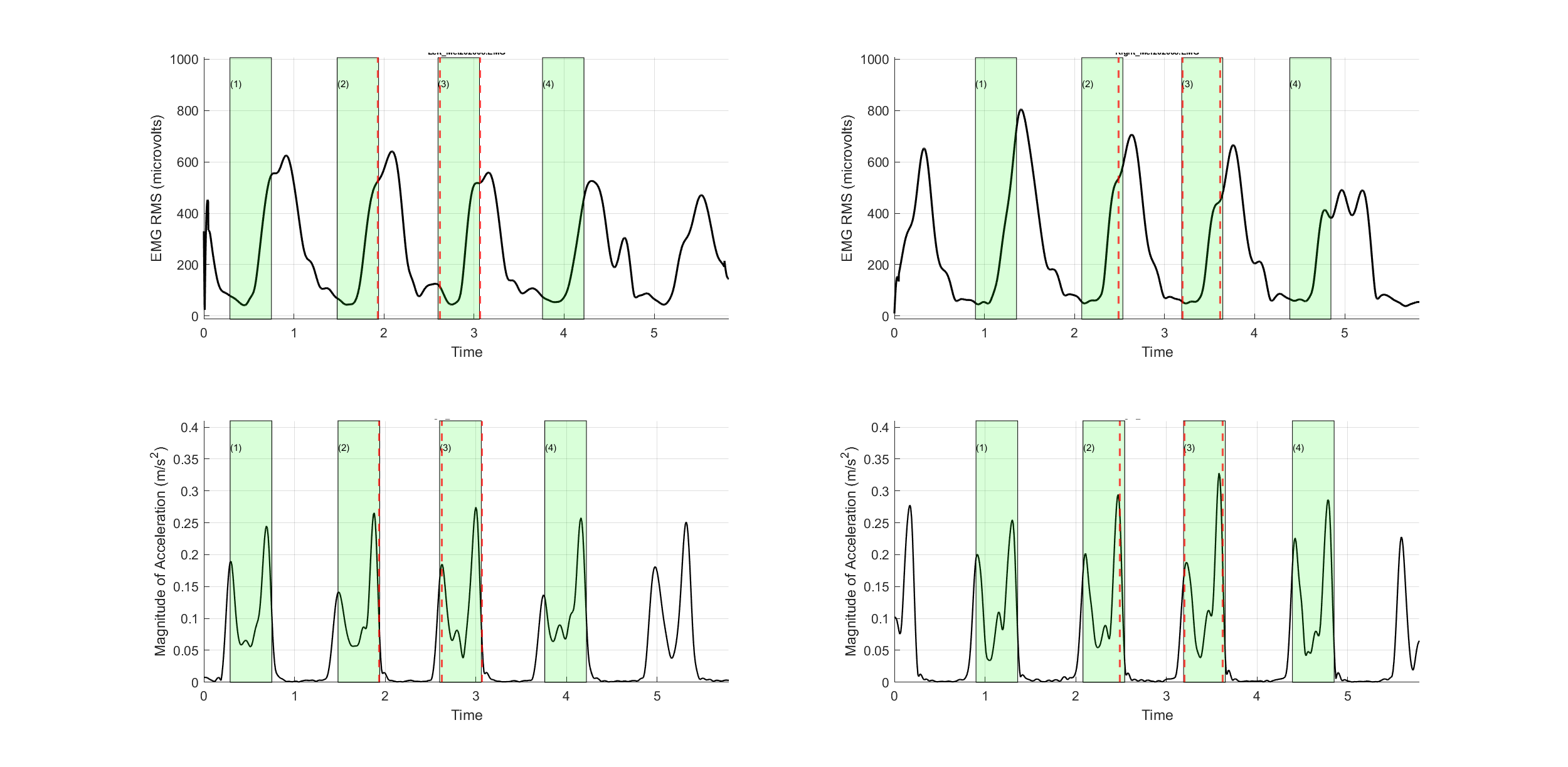

Supplement: Supplementary file 1 [file sensors-22-04957-s001.zip › Part 1 - 3D CGA historic patient data partitions/Figure_5202008.png]

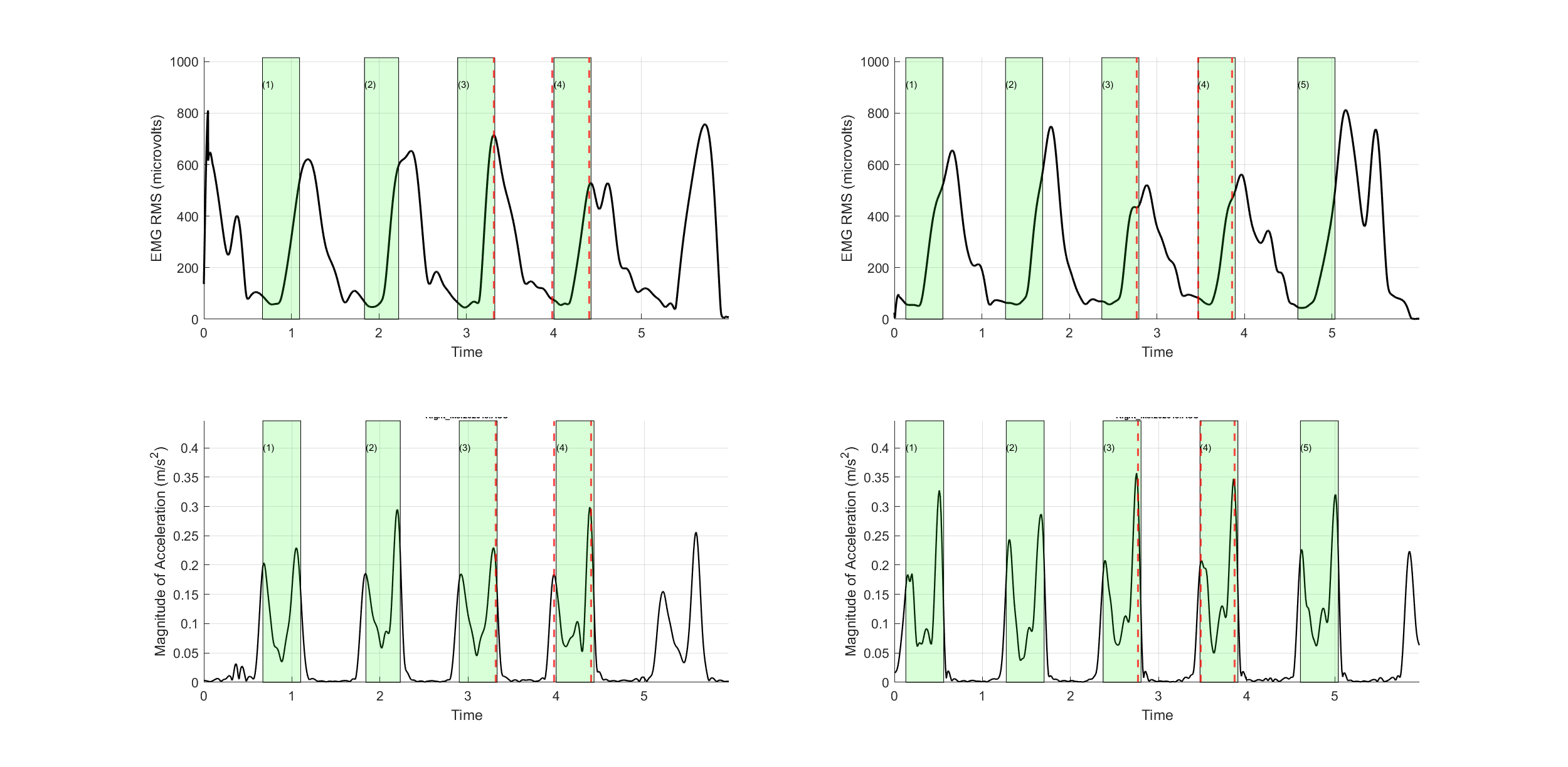

Supplement: Supplementary file 1 [file sensors-22-04957-s001.zip › Part 1 - 3D CGA historic patient data partitions/Figure_5202013.png]

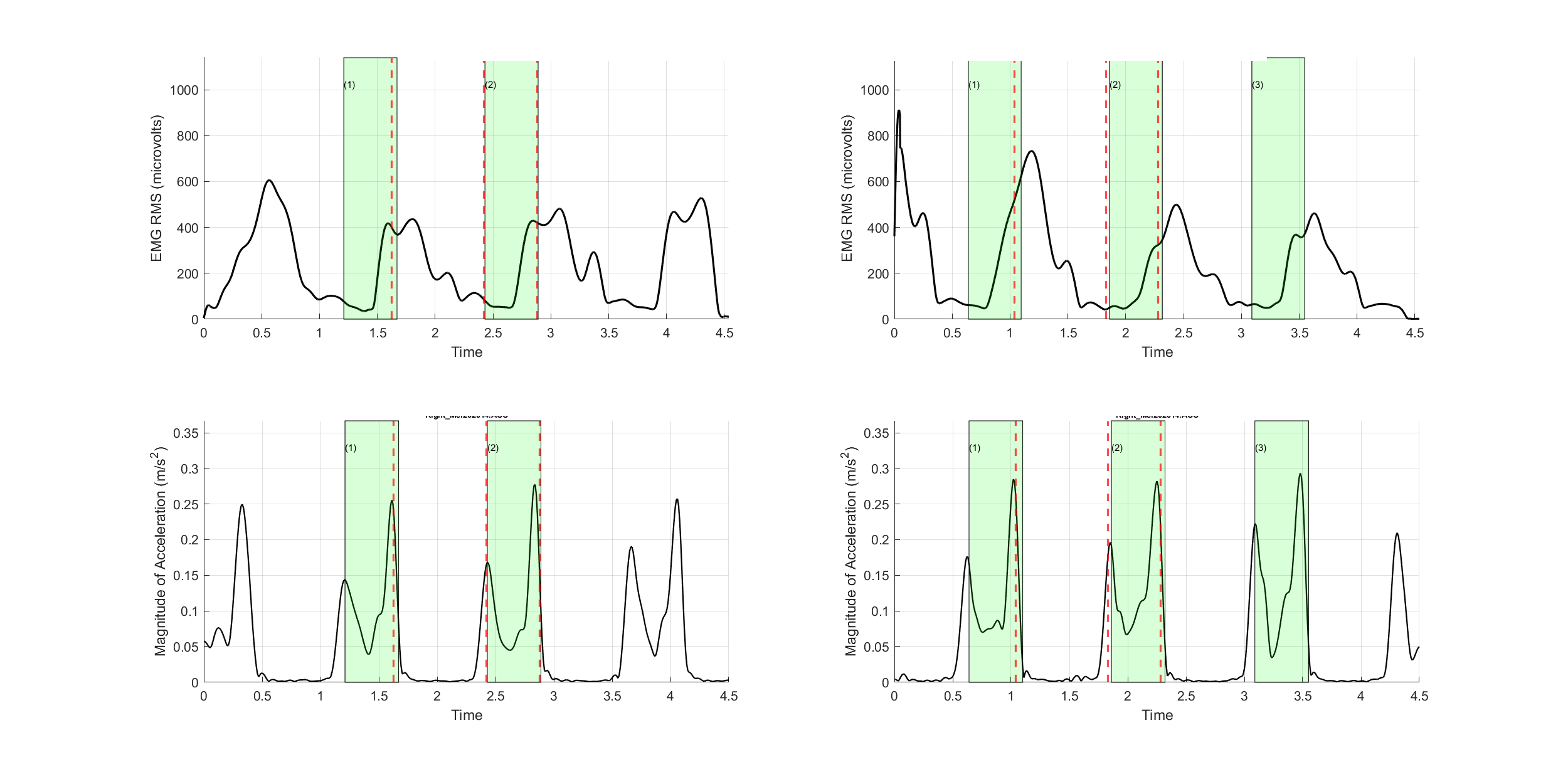

Supplement: Supplementary file 1 [file sensors-22-04957-s001.zip › Part 1 - 3D CGA historic patient data partitions/Figure_5202014.png]

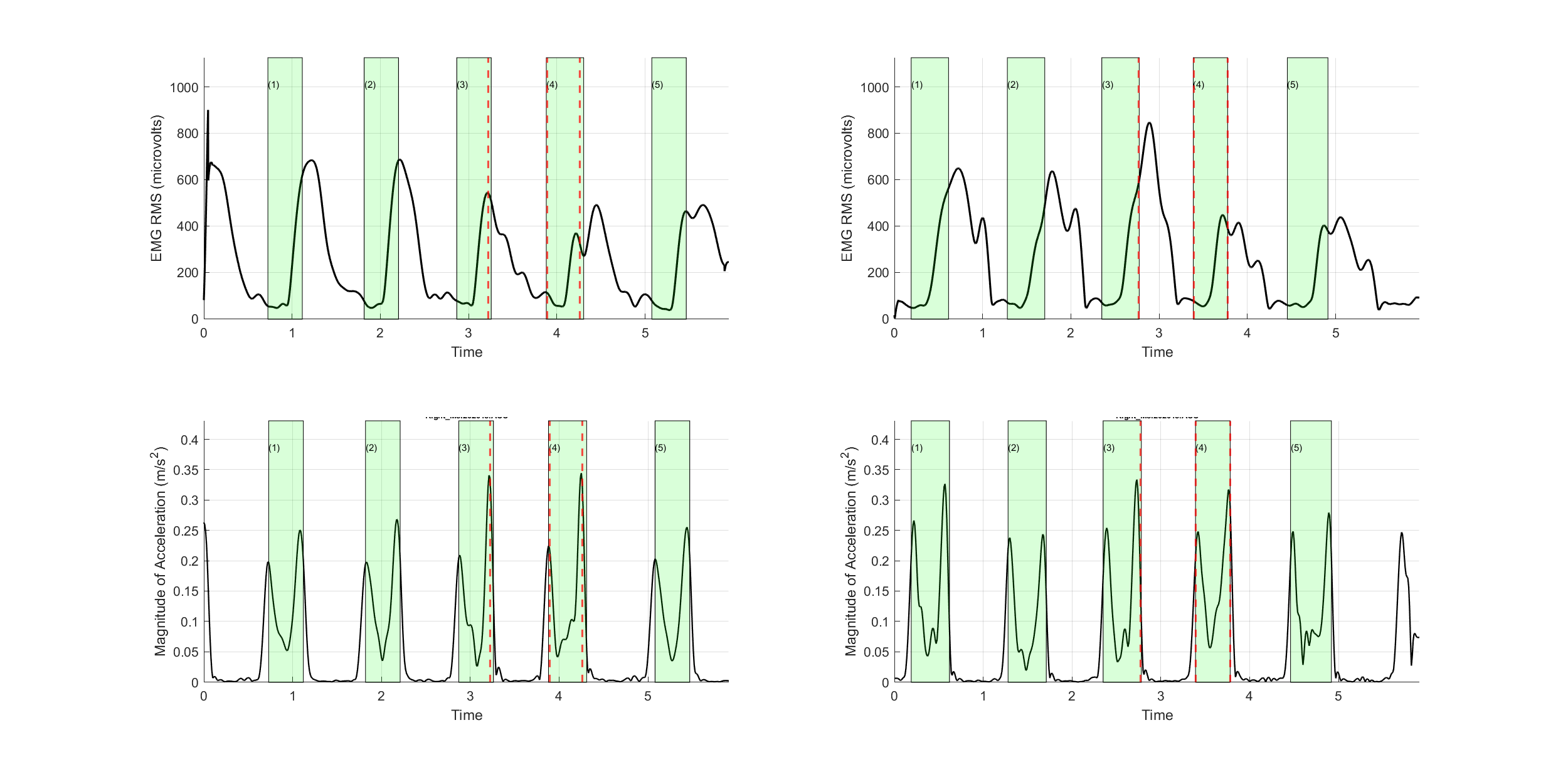

Supplement: Supplementary file 1 [file sensors-22-04957-s001.zip › Part 1 - 3D CGA historic patient data partitions/Figure_5202015.png]

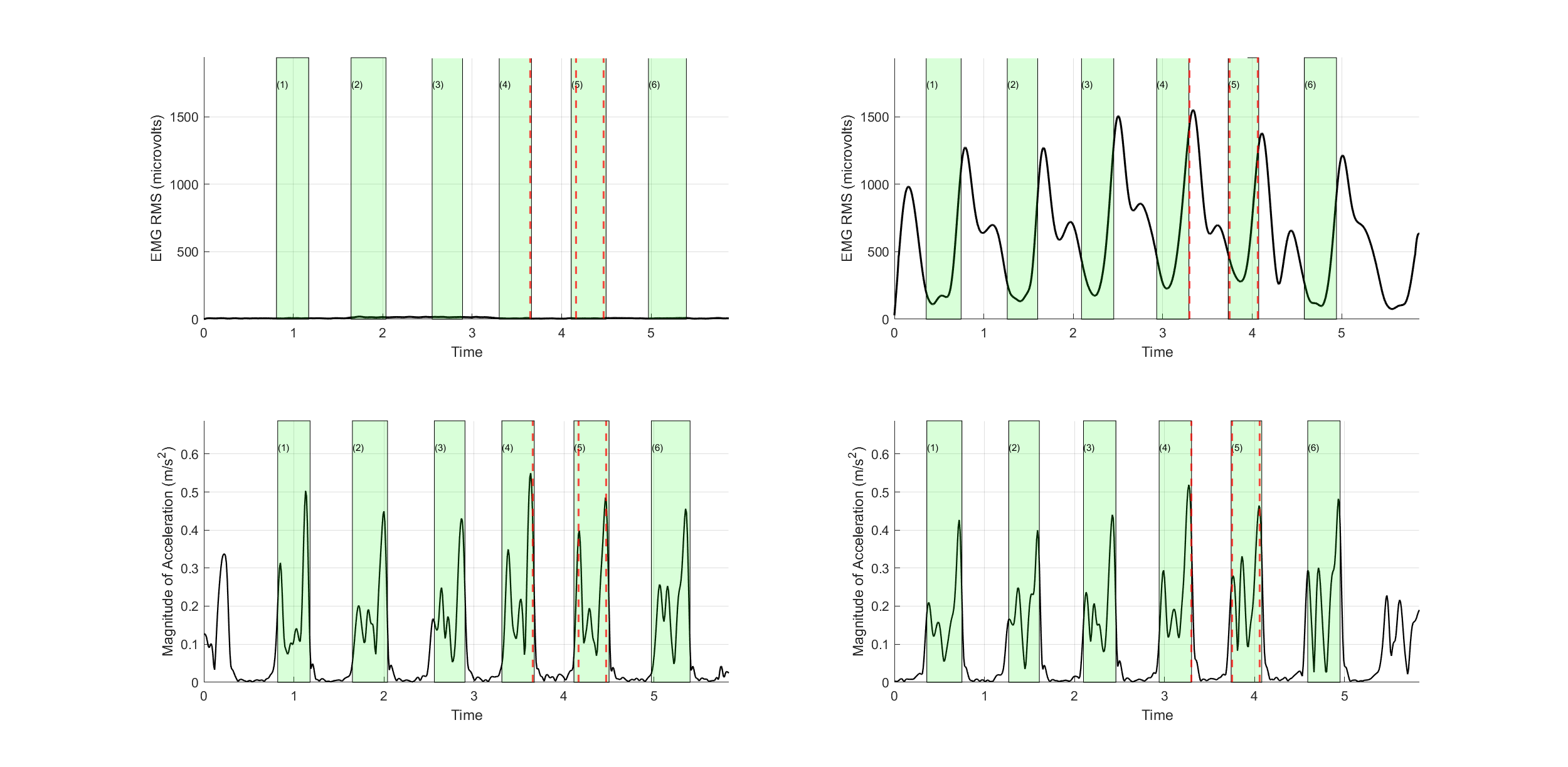

Supplement: Supplementary file 1 [file sensors-22-04957-s001.zip › Part 1 - 3D CGA historic patient data partitions/Figure_5202106.png]

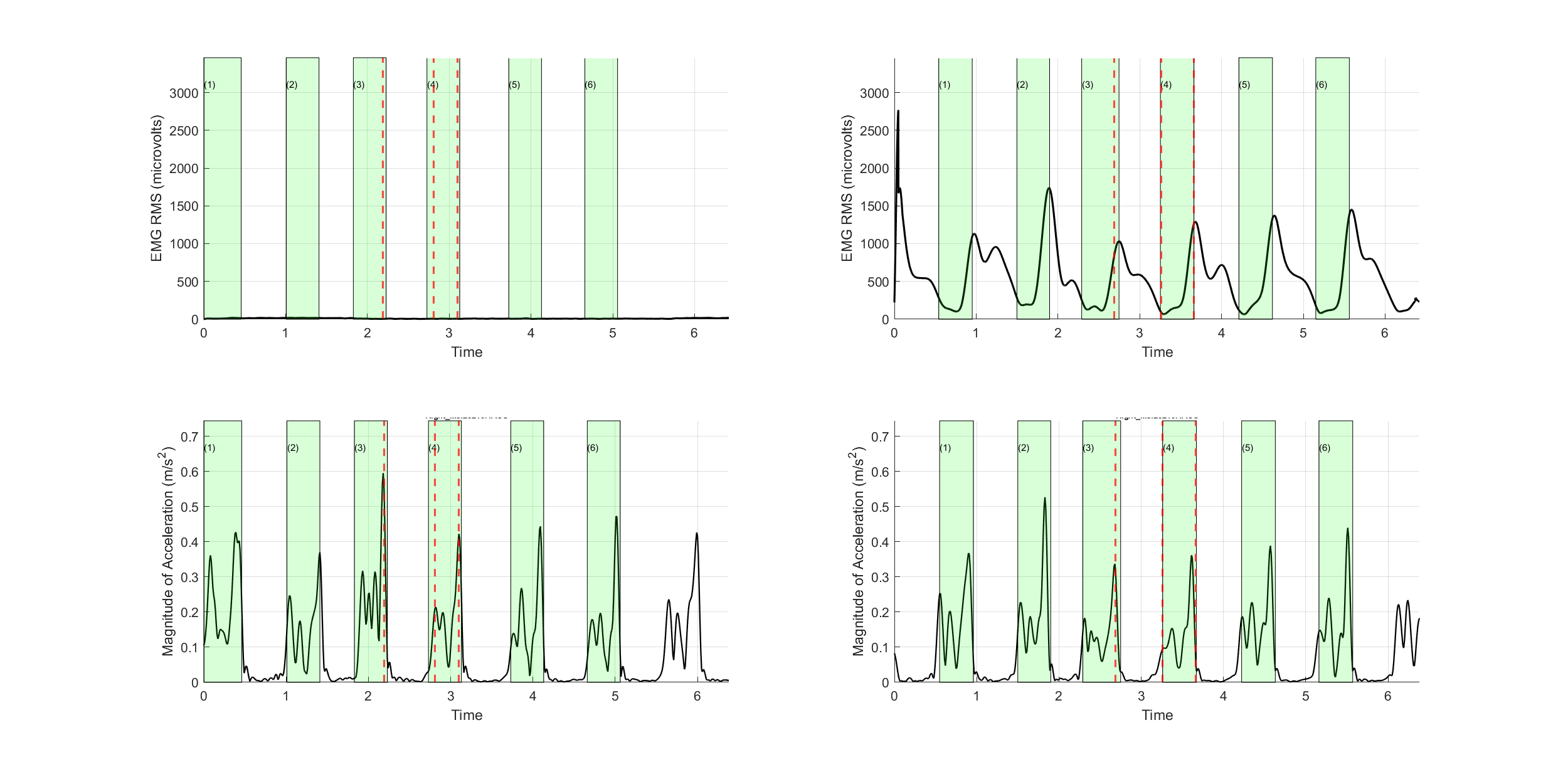

Supplement: Supplementary file 1 [file sensors-22-04957-s001.zip › Part 1 - 3D CGA historic patient data partitions/Figure_5202107.png]

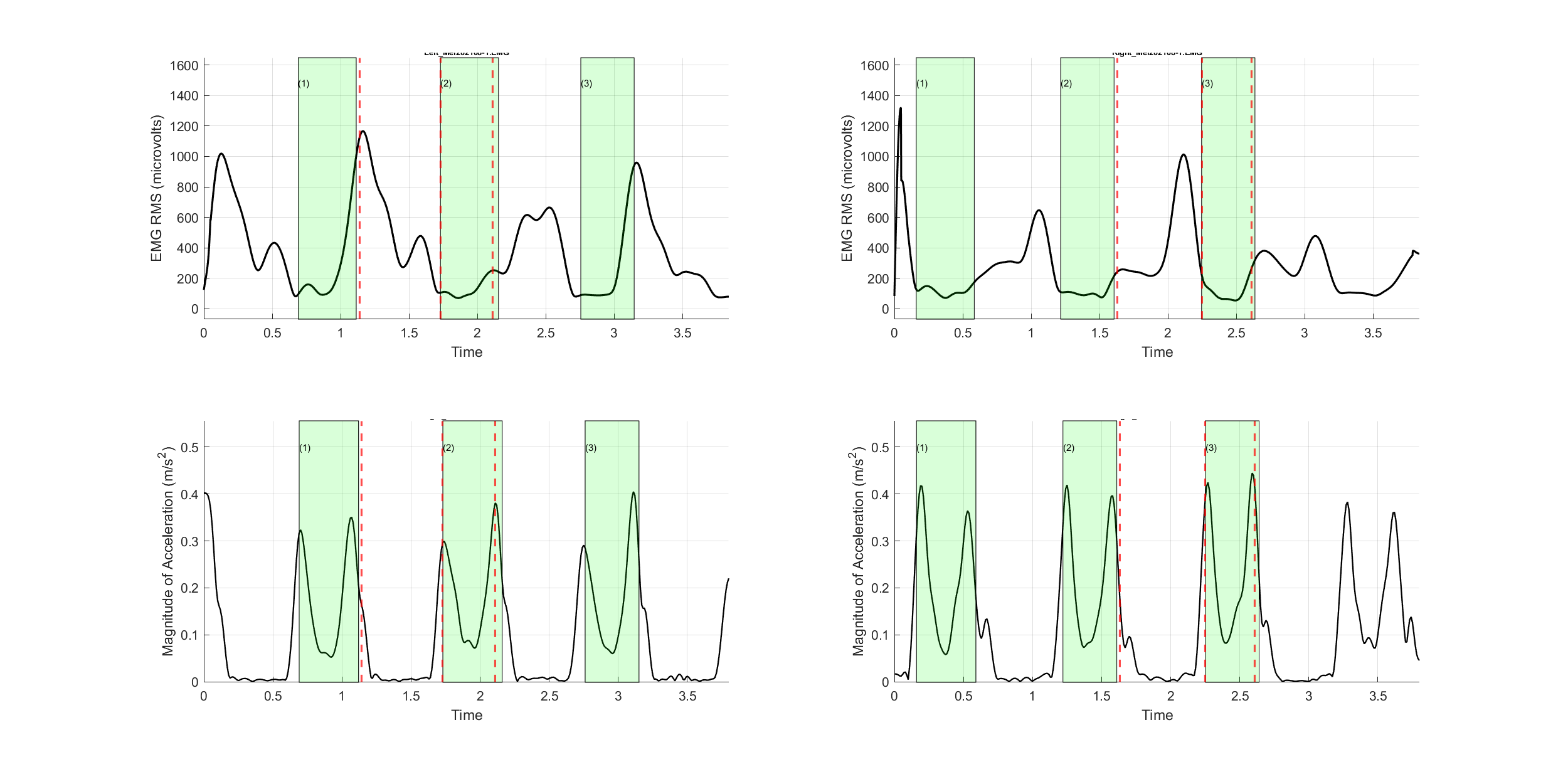

Supplement: Supplementary file 1 [file sensors-22-04957-s001.zip › Part 1 - 3D CGA historic patient data partitions/Figure_5202108-1.png]

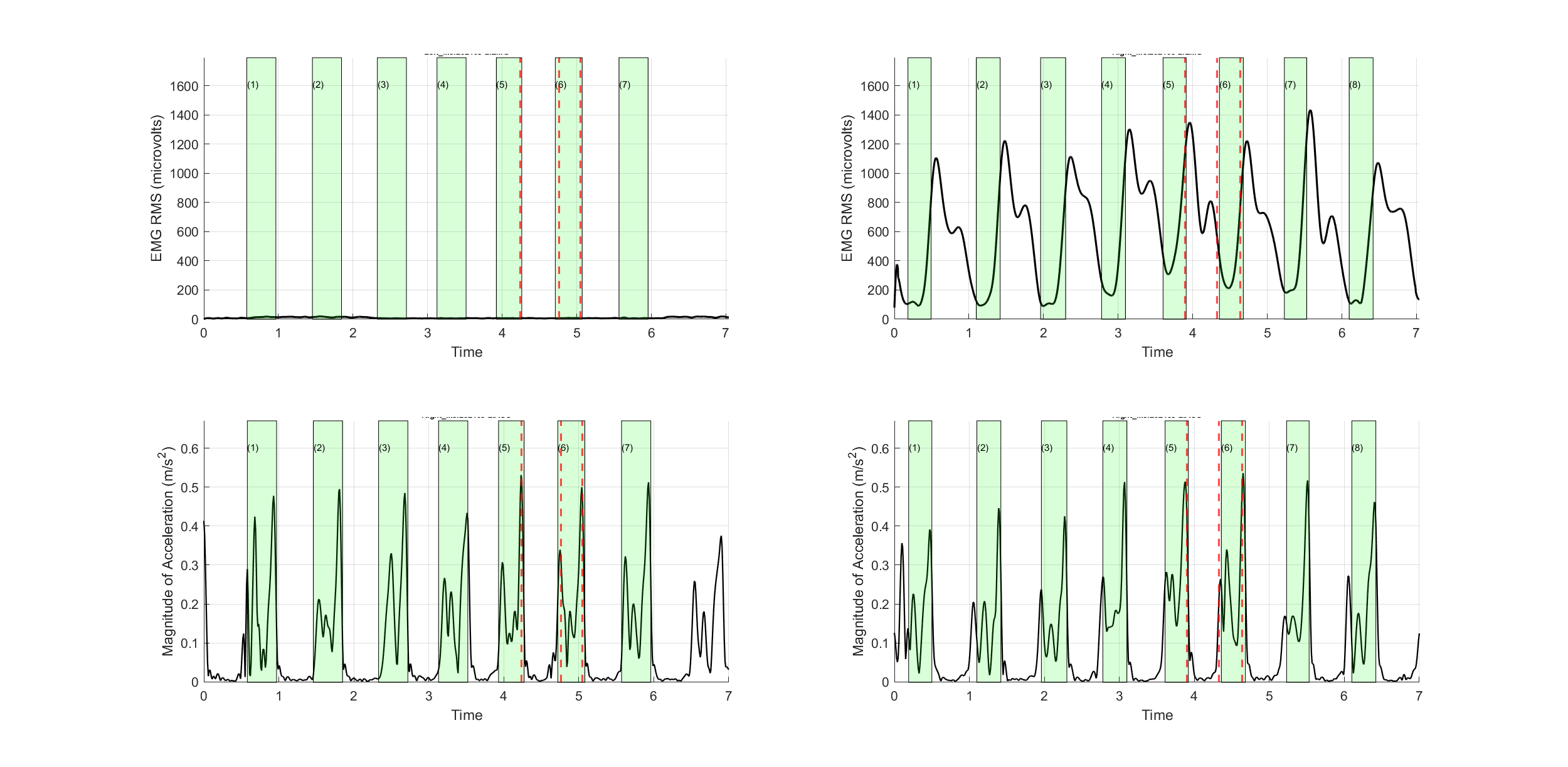

Supplement: Supplementary file 1 [file sensors-22-04957-s001.zip › Part 1 - 3D CGA historic patient data partitions/Figure_5202108-2.png]

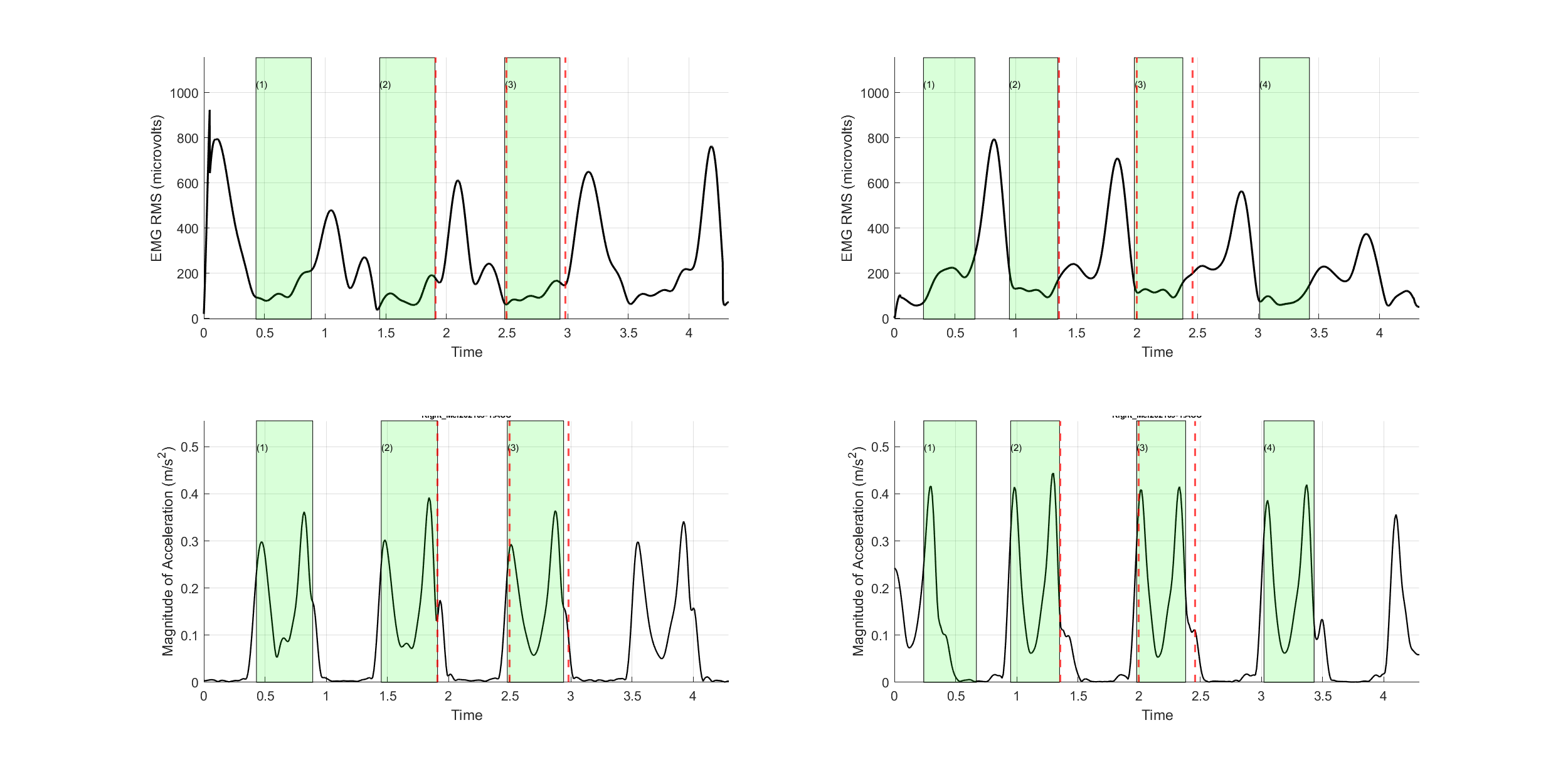

Supplement: Supplementary file 1 [file sensors-22-04957-s001.zip › Part 1 - 3D CGA historic patient data partitions/Figure_5202109-1.png]

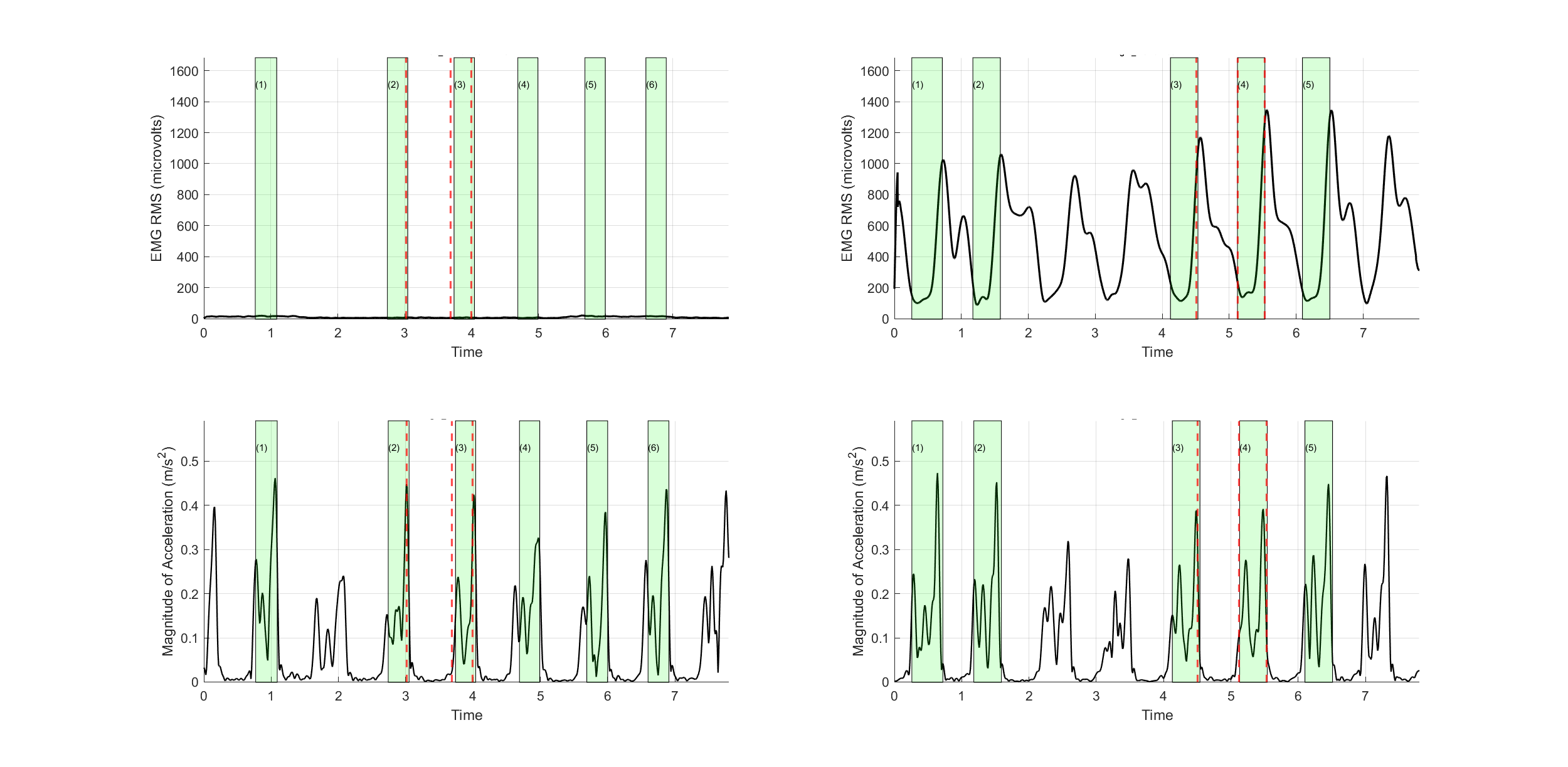

Supplement: Supplementary file 1 [file sensors-22-04957-s001.zip › Part 1 - 3D CGA historic patient data partitions/Figure_5202109-2.png]

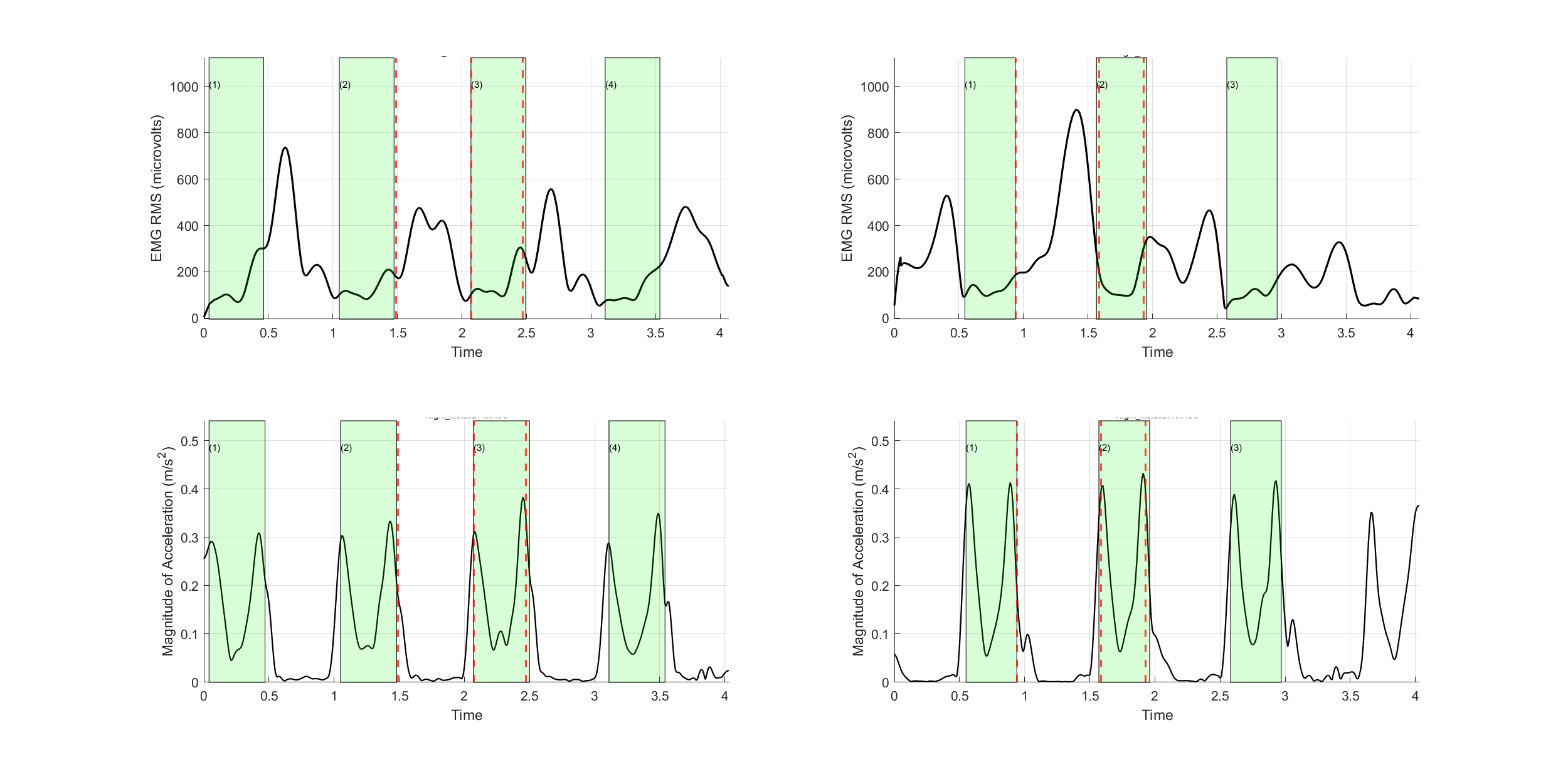

Supplement: Supplementary file 1 [file sensors-22-04957-s001.zip › Part 1 - 3D CGA historic patient data partitions/Figure_5202110.png]

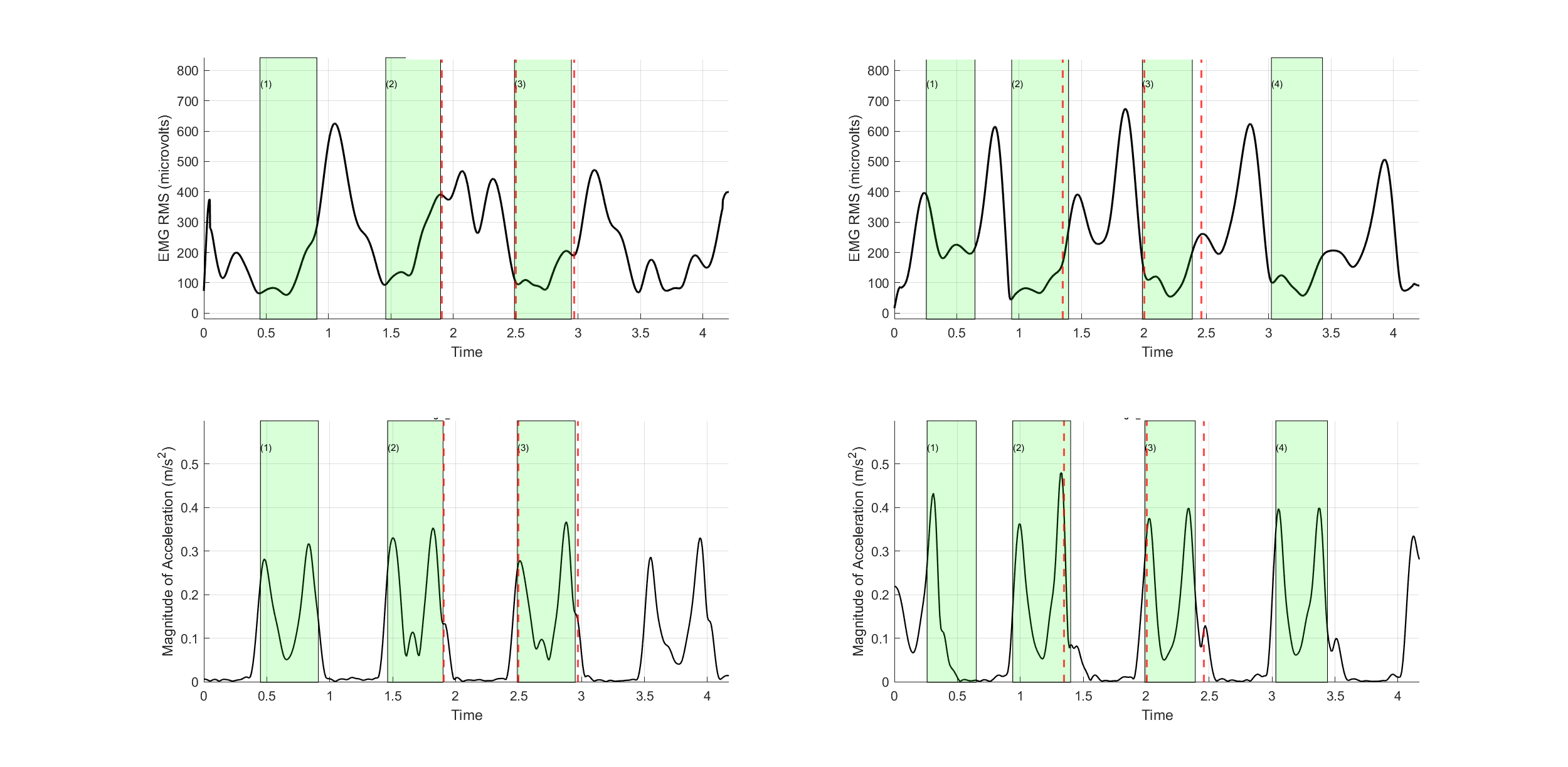

Supplement: Supplementary file 1 [file sensors-22-04957-s001.zip › Part 1 - 3D CGA historic patient data partitions/Figure_5202111.png]

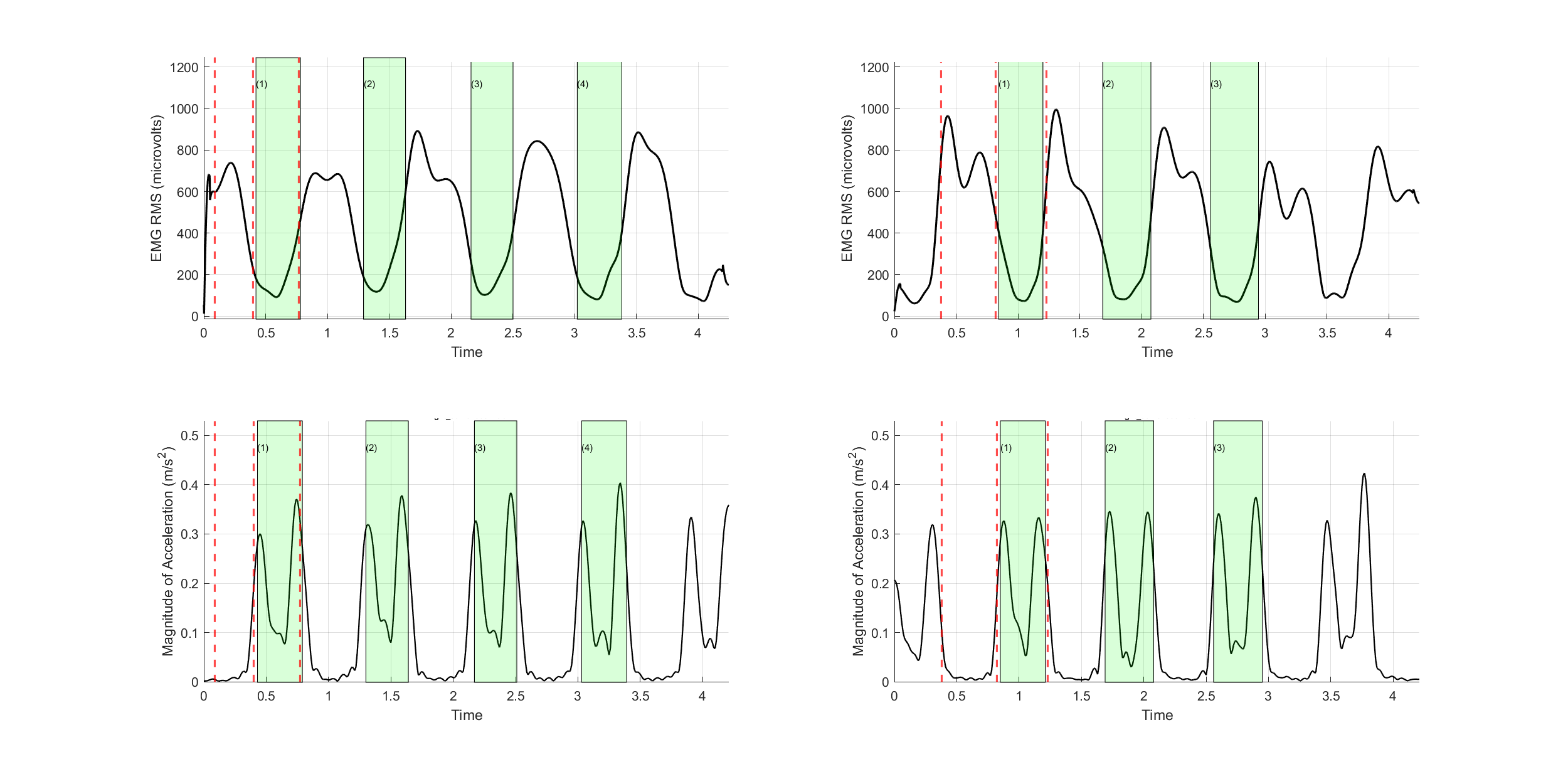

Supplement: Supplementary file 1 [file sensors-22-04957-s001.zip › Part 1 - 3D CGA historic patient data partitions/Figure_7202003.png]

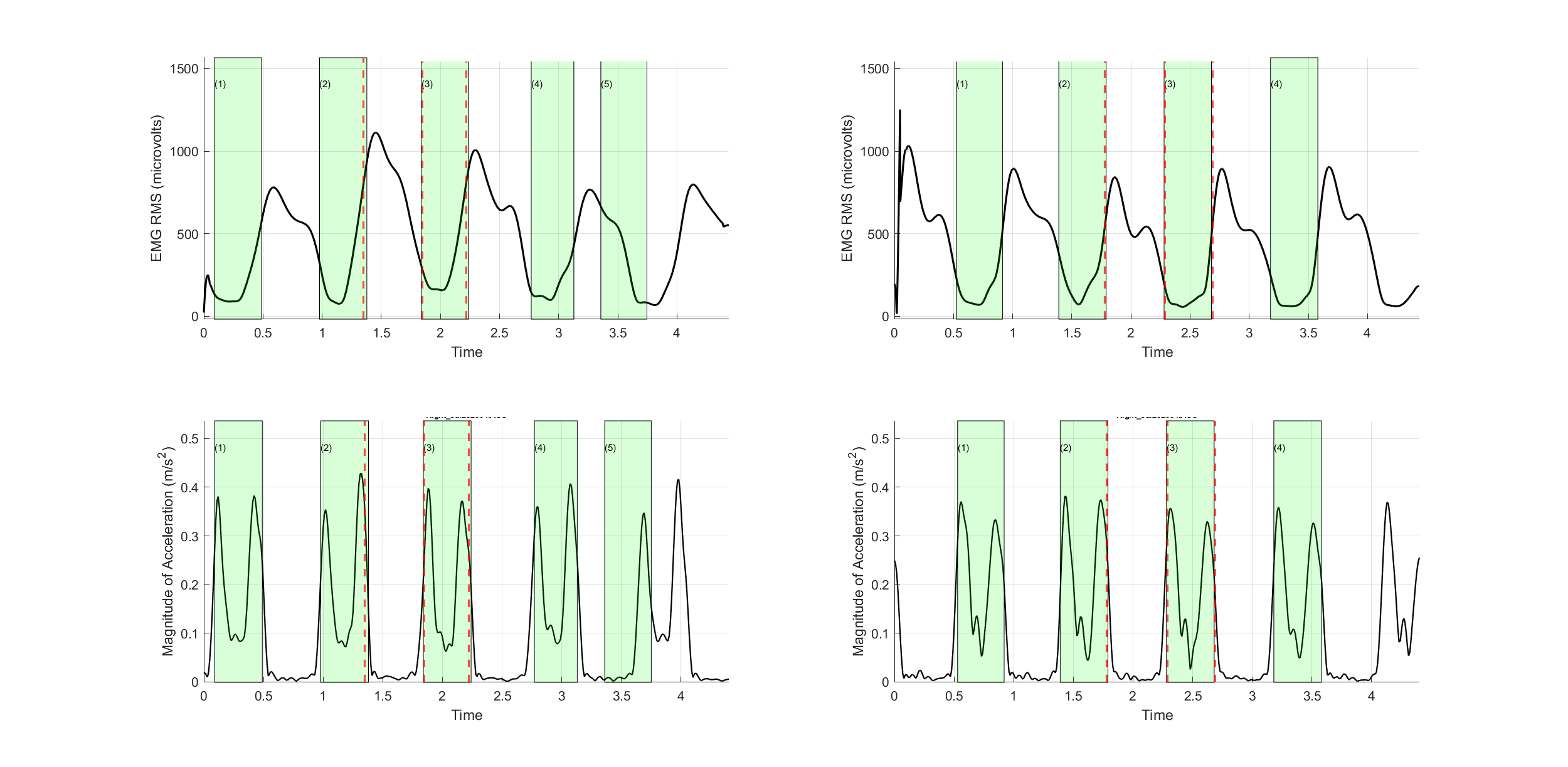

Supplement: Supplementary file 1 [file sensors-22-04957-s001.zip › Part 1 - 3D CGA historic patient data partitions/Figure_7202004.png]

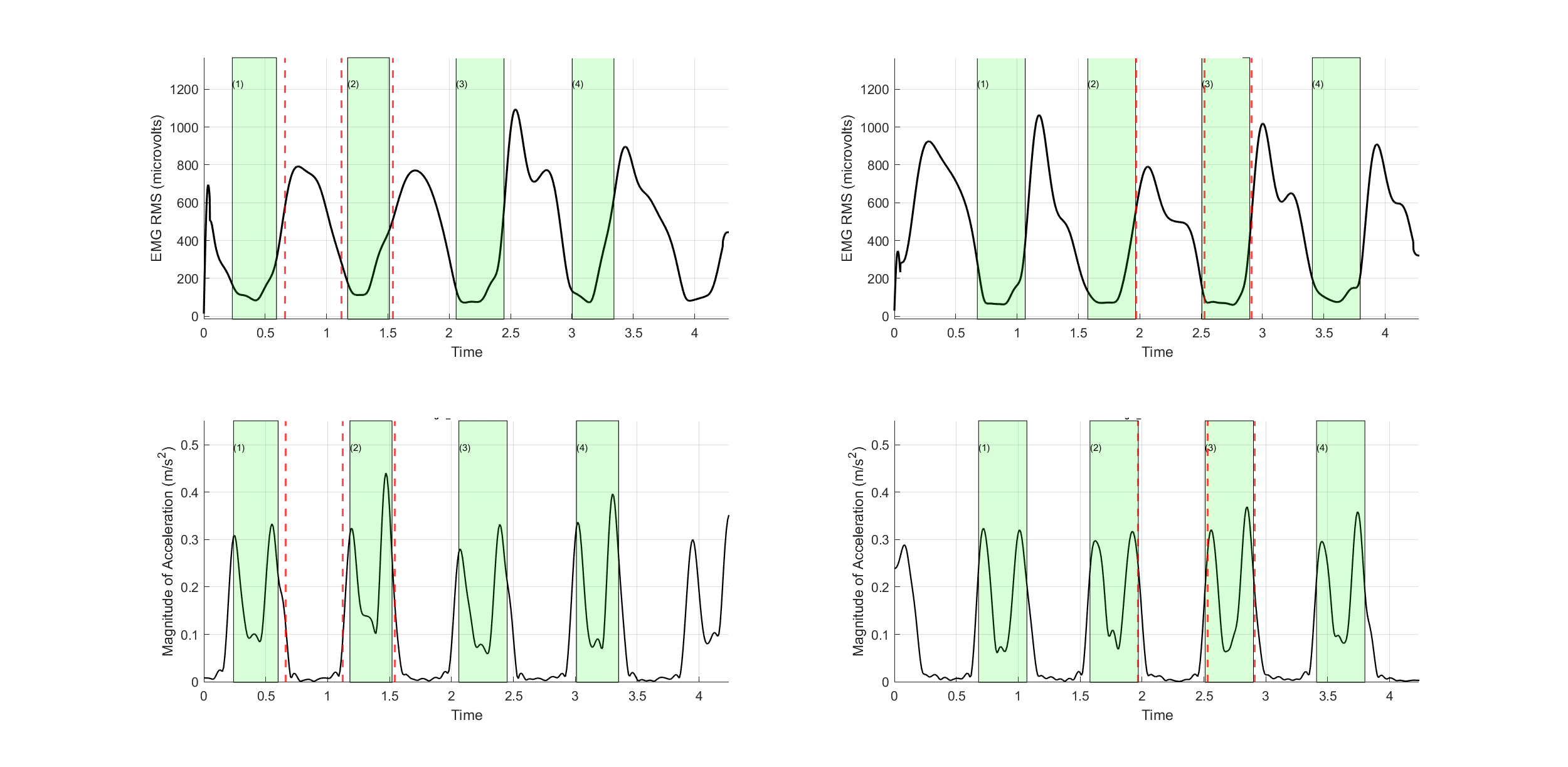

Supplement: Supplementary file 1 [file sensors-22-04957-s001.zip › Part 1 - 3D CGA historic patient data partitions/Figure_7202005.png]

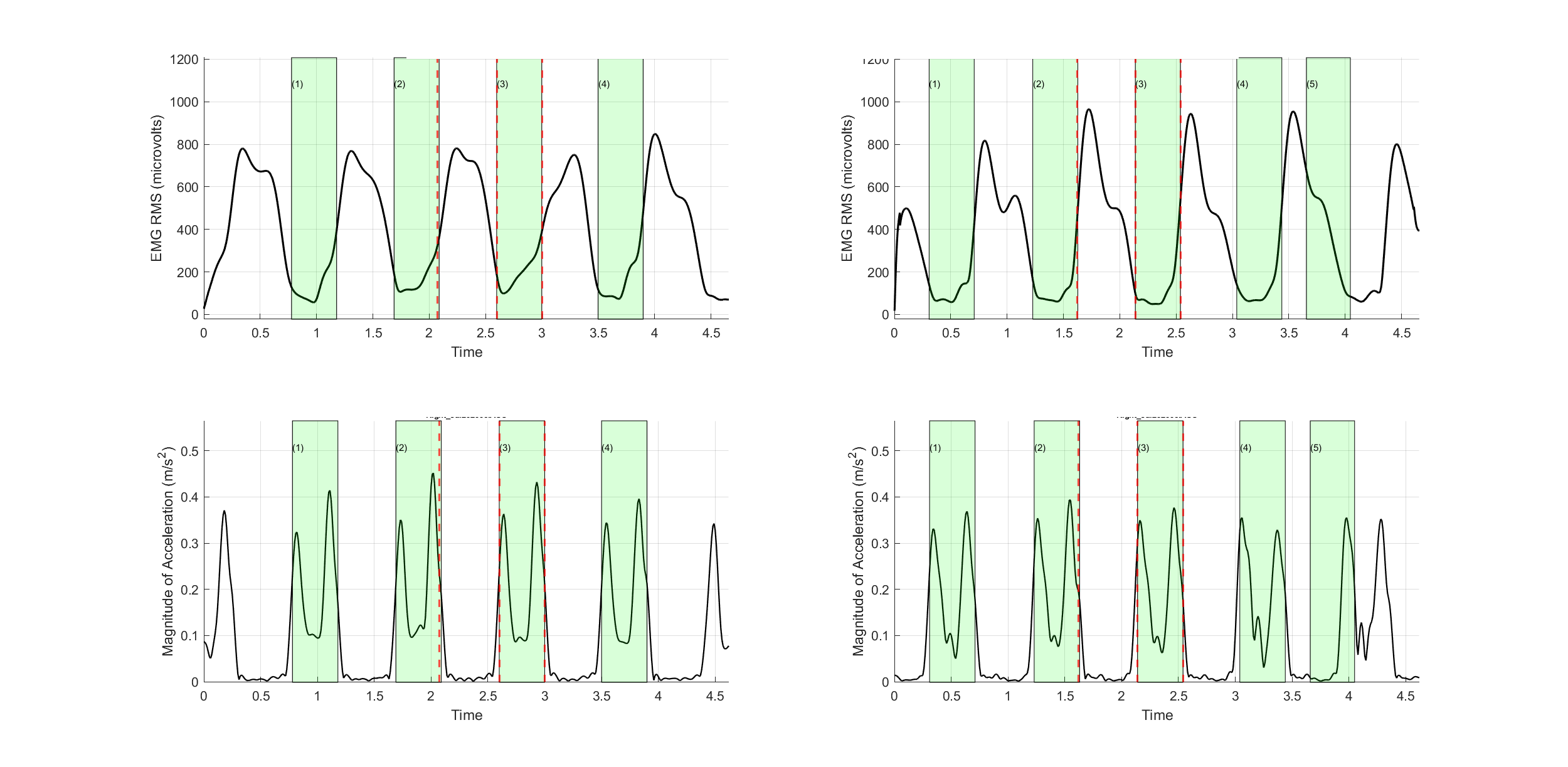

Supplement: Supplementary file 1 [file sensors-22-04957-s001.zip › Part 1 - 3D CGA historic patient data partitions/Figure_7202006.png]

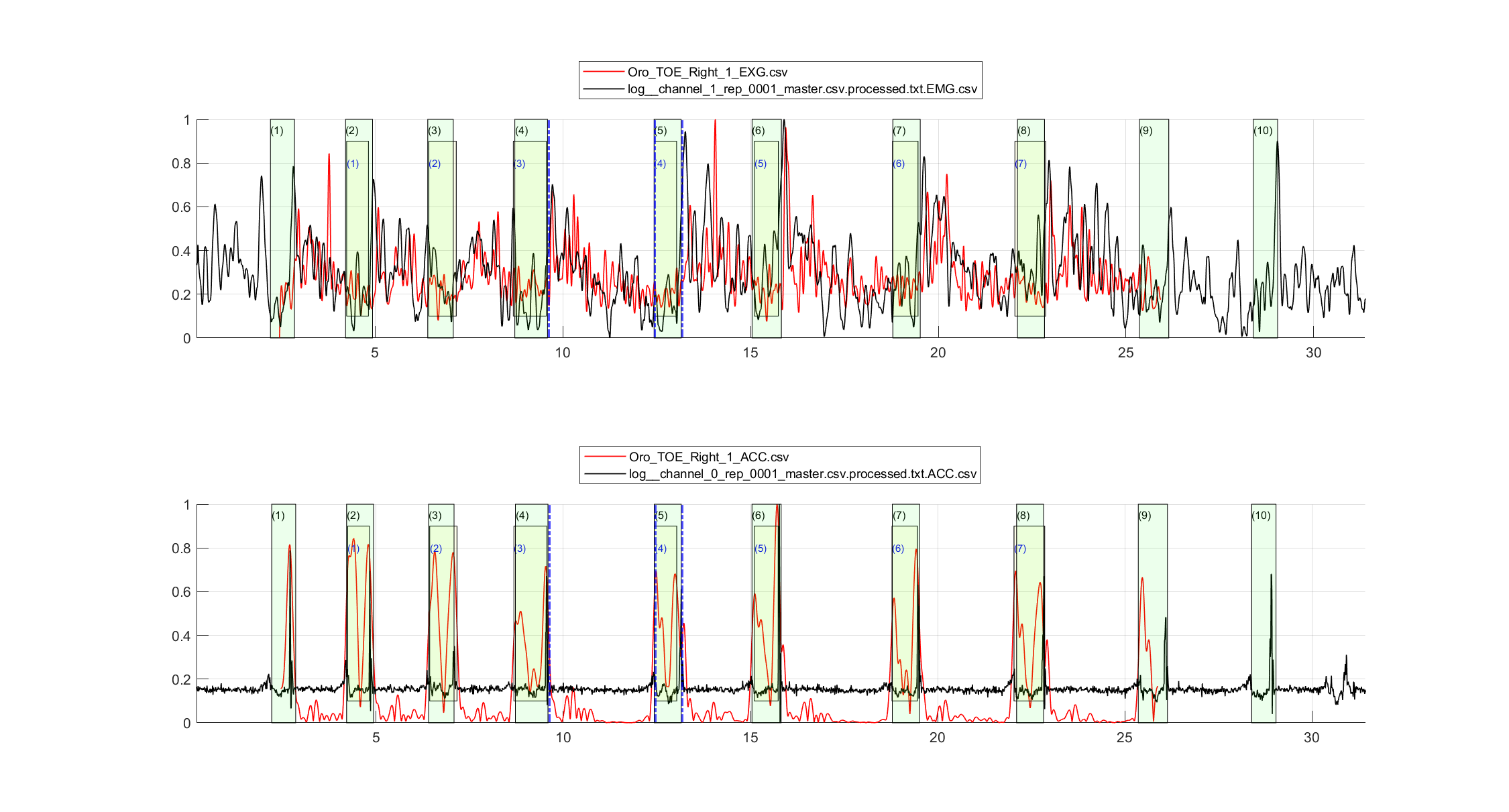

Supplement: Supplementary file 1 [file sensors-22-04957-s001.zip › Part 2 - 3D CGA vs oro sensor system data partitioning/Patient 1-1 barefoot/Figure_Oro_TOE_Right_1.png]

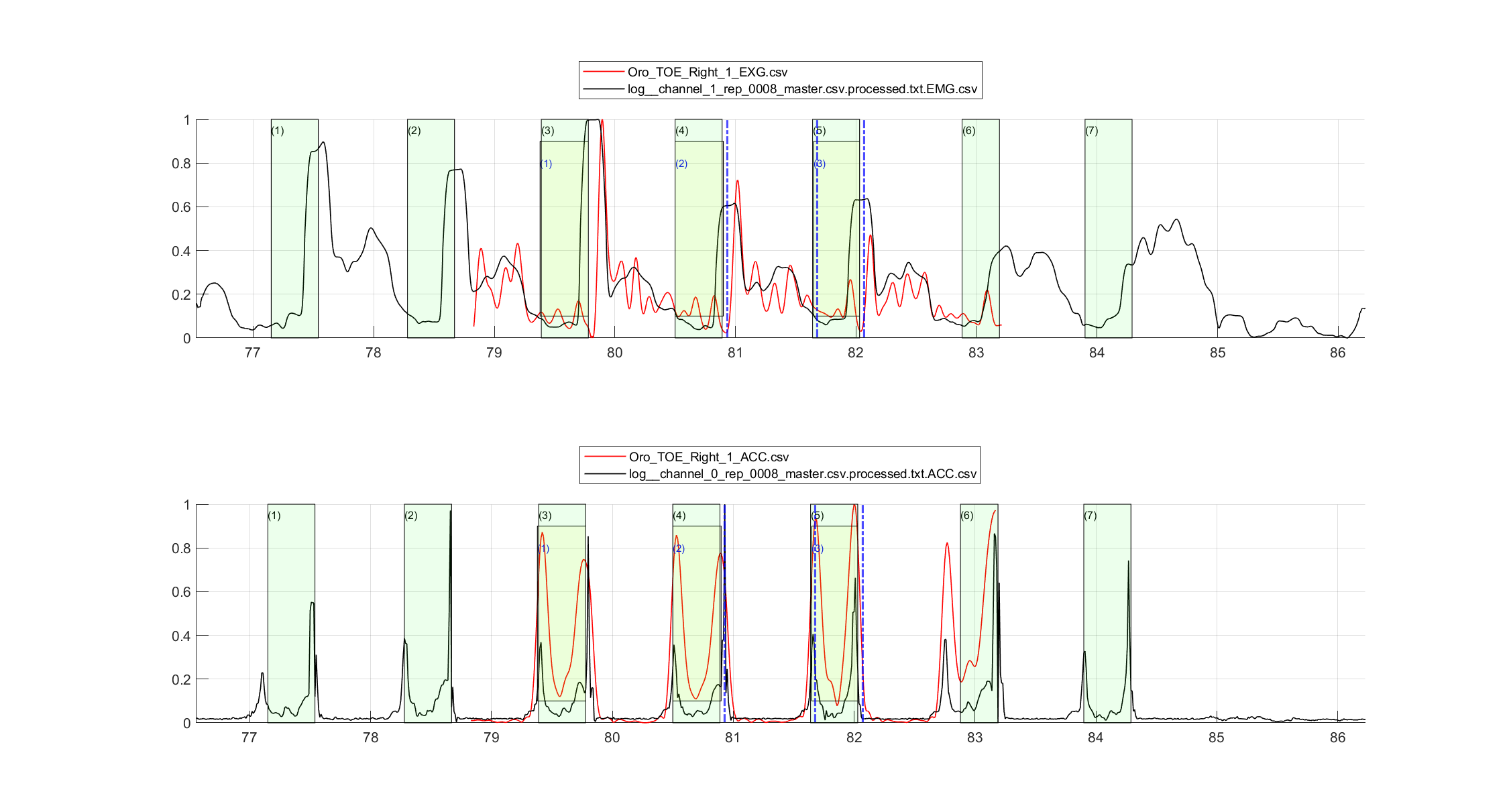

Supplement: Supplementary file 1 [file sensors-22-04957-s001.zip › Part 2 - 3D CGA vs oro sensor system data partitioning/Patient 2-1 barefoot/Figure_Oro_TOE_Right_1.png]

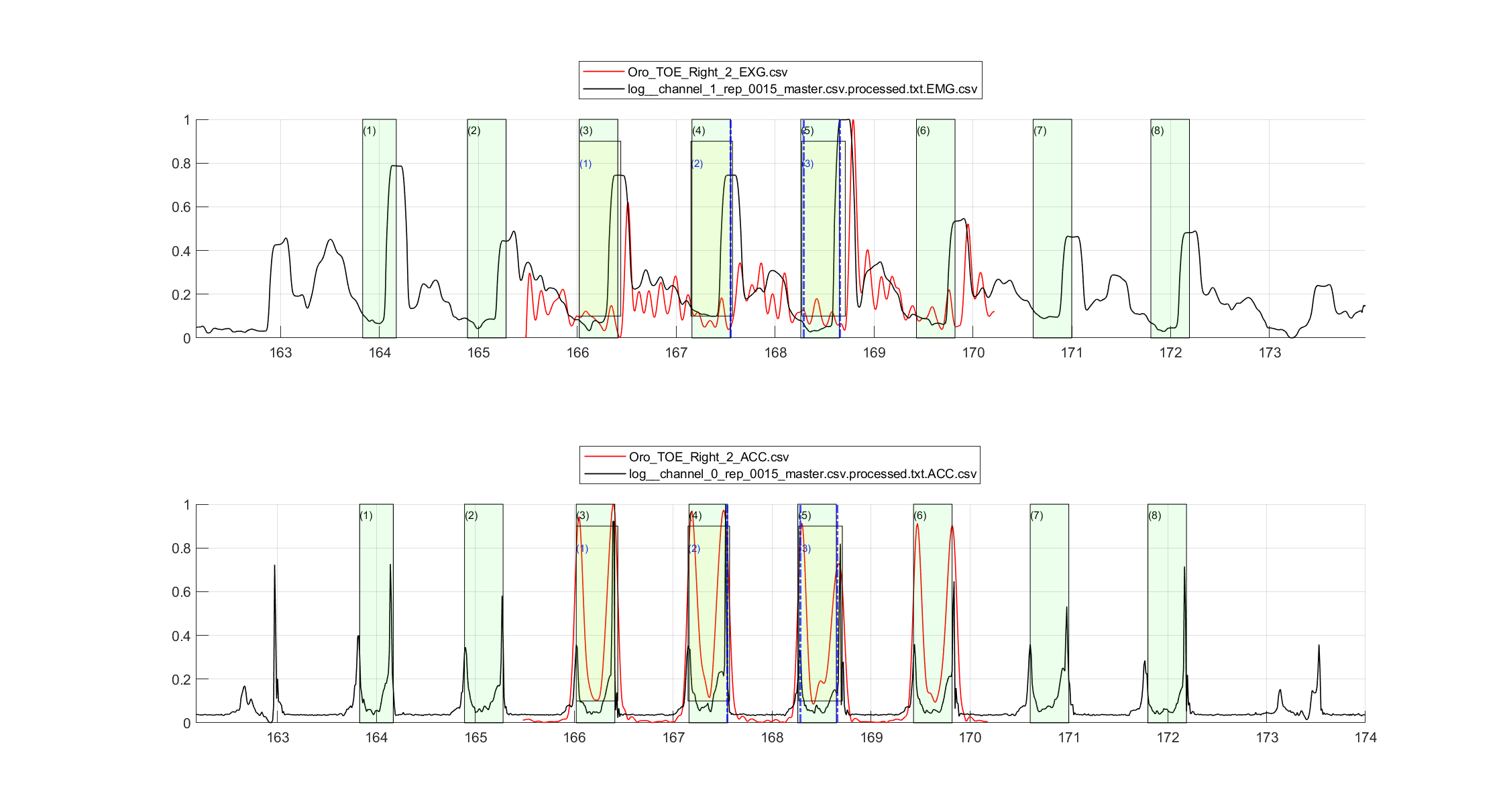

Supplement: Supplementary file 1 [file sensors-22-04957-s001.zip › Part 2 - 3D CGA vs oro sensor system data partitioning/Patient 2-1 barefoot/Figure_Oro_TOE_Right_2.png]

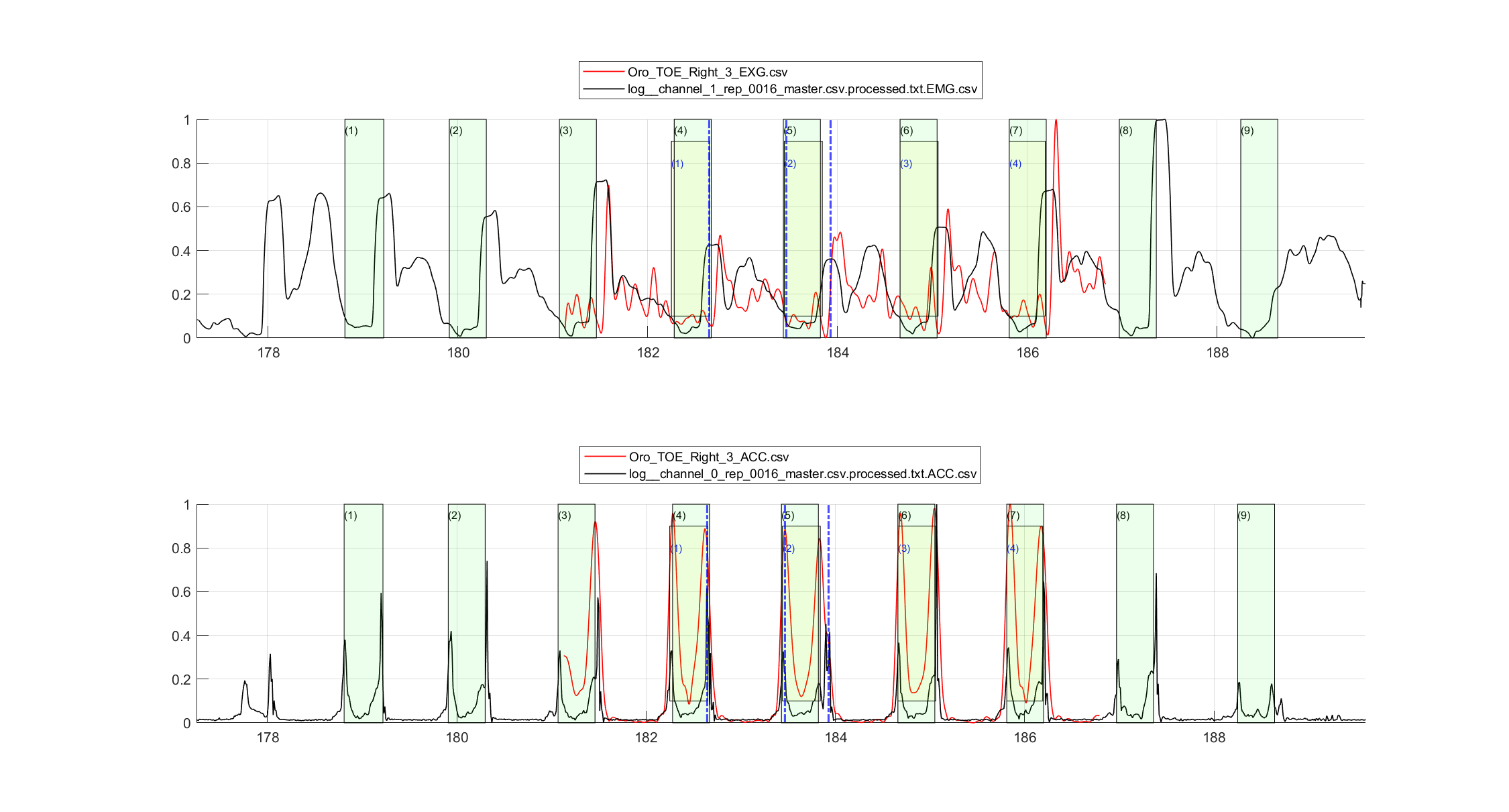

Supplement: Supplementary file 1 [file sensors-22-04957-s001.zip › Part 2 - 3D CGA vs oro sensor system data partitioning/Patient 2-1 barefoot/Figure_Oro_TOE_Right_3.png]

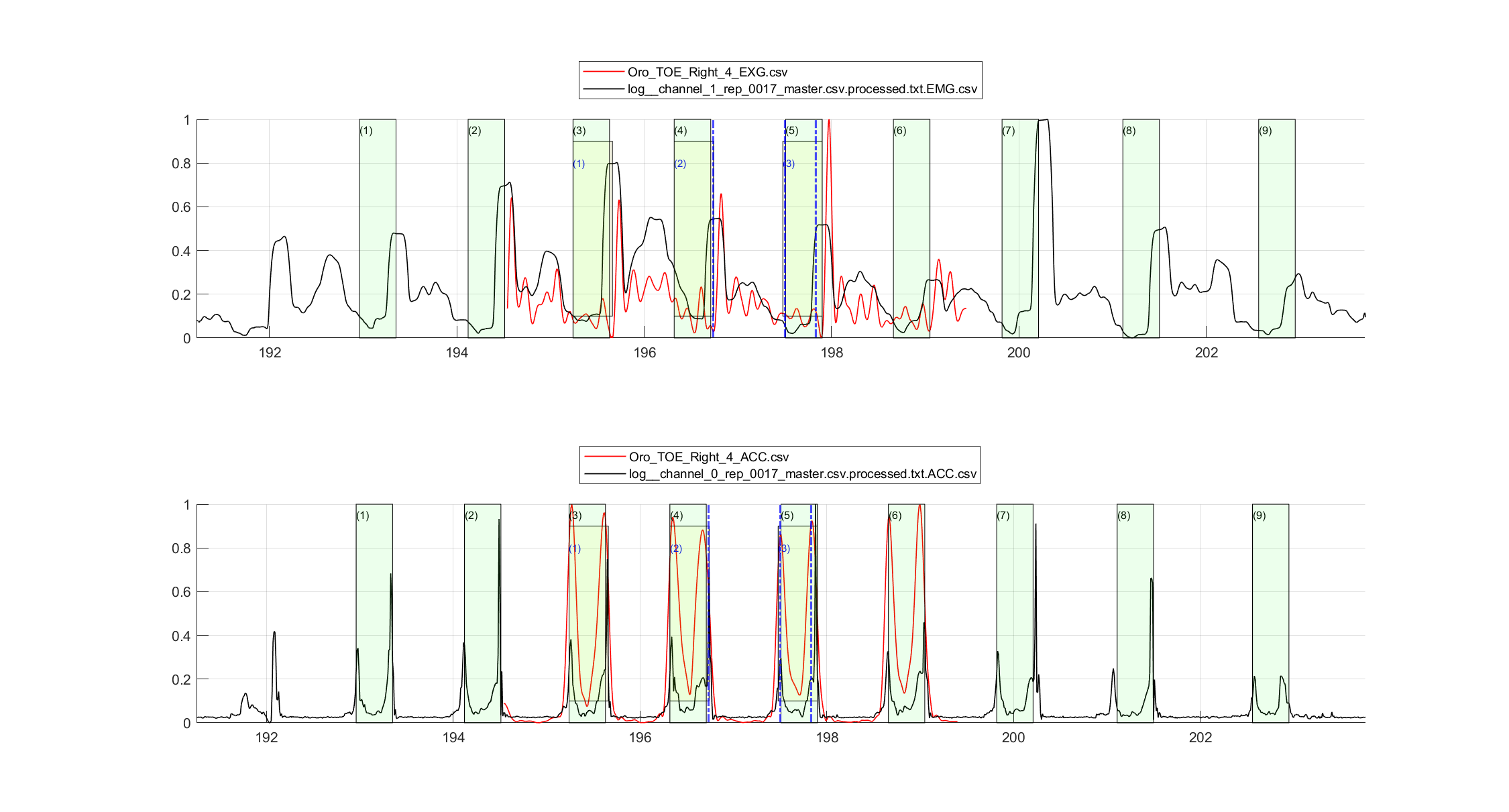

Supplement: Supplementary file 1 [file sensors-22-04957-s001.zip › Part 2 - 3D CGA vs oro sensor system data partitioning/Patient 2-1 barefoot/Figure_Oro_TOE_Right_4.png]

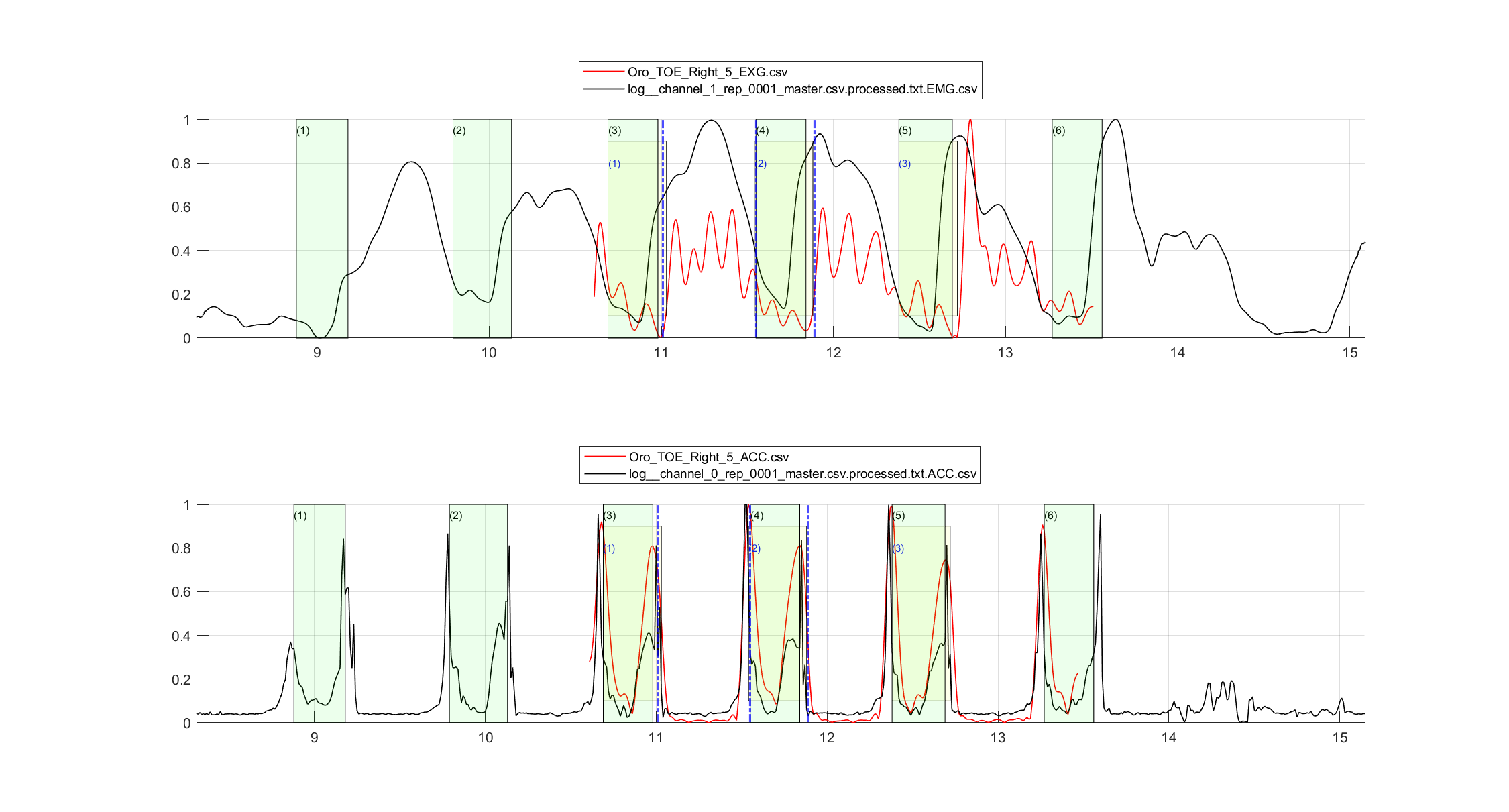

Supplement: Supplementary file 1 [file sensors-22-04957-s001.zip › Part 2 - 3D CGA vs oro sensor system data partitioning/Patient 2-2 barefoot fast/Figure_Oro_TOE_Right_5.png]

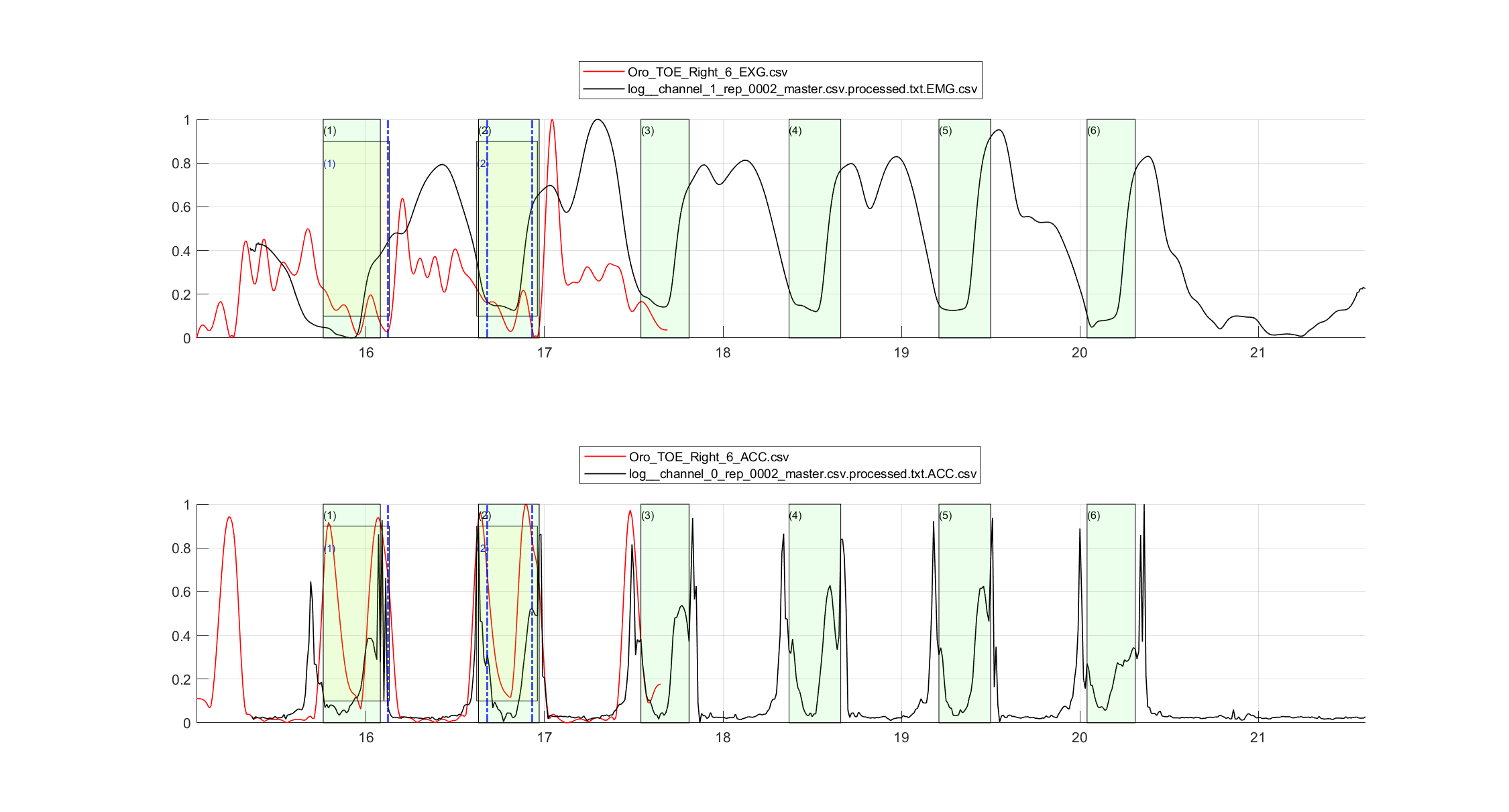

Supplement: Supplementary file 1 [file sensors-22-04957-s001.zip › Part 2 - 3D CGA vs oro sensor system data partitioning/Patient 2-2 barefoot fast/Figure_Oro_TOE_Right_6.png]

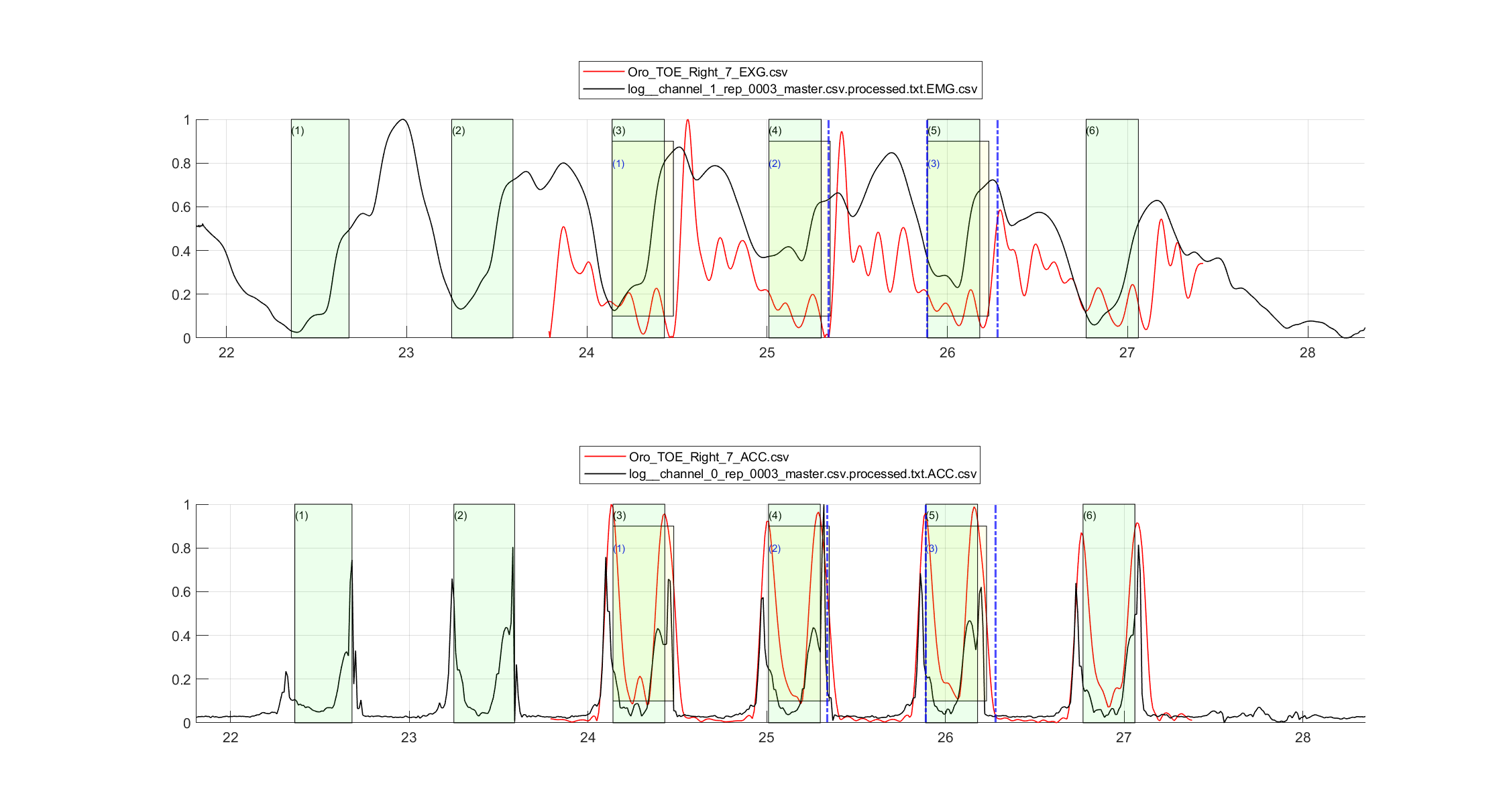

Supplement: Supplementary file 1 [file sensors-22-04957-s001.zip › Part 2 - 3D CGA vs oro sensor system data partitioning/Patient 2-2 barefoot fast/Figure_Oro_TOE_Right_7.png]

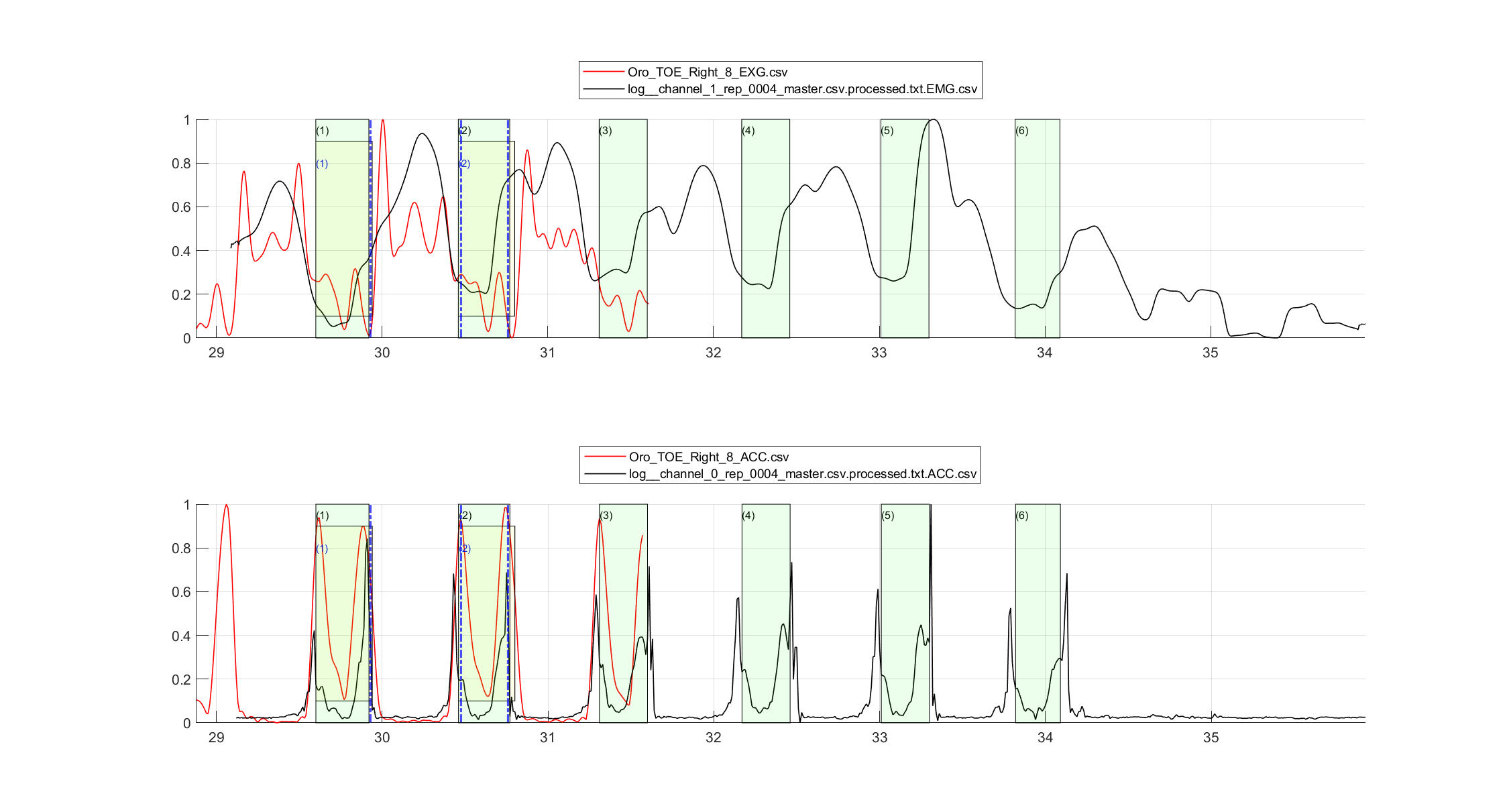

Supplement: Supplementary file 1 [file sensors-22-04957-s001.zip › Part 2 - 3D CGA vs oro sensor system data partitioning/Patient 2-2 barefoot fast/Figure_Oro_TOE_Right_8.png]

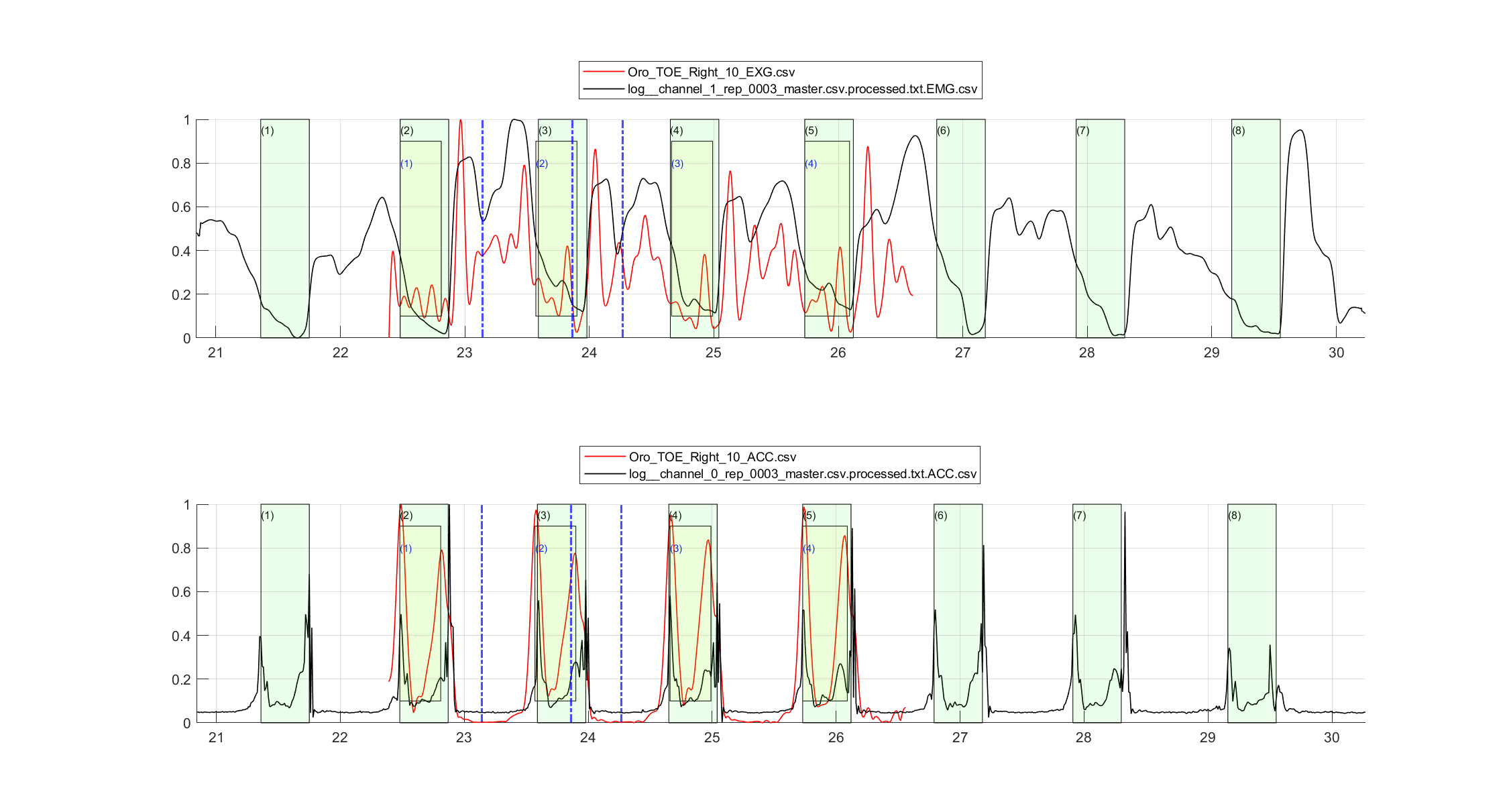

Supplement: Supplementary file 1 [file sensors-22-04957-s001.zip › Part 2 - 3D CGA vs oro sensor system data partitioning/Patient 2-3 shoes/Figure_Oro_TOE_Right_10.png]

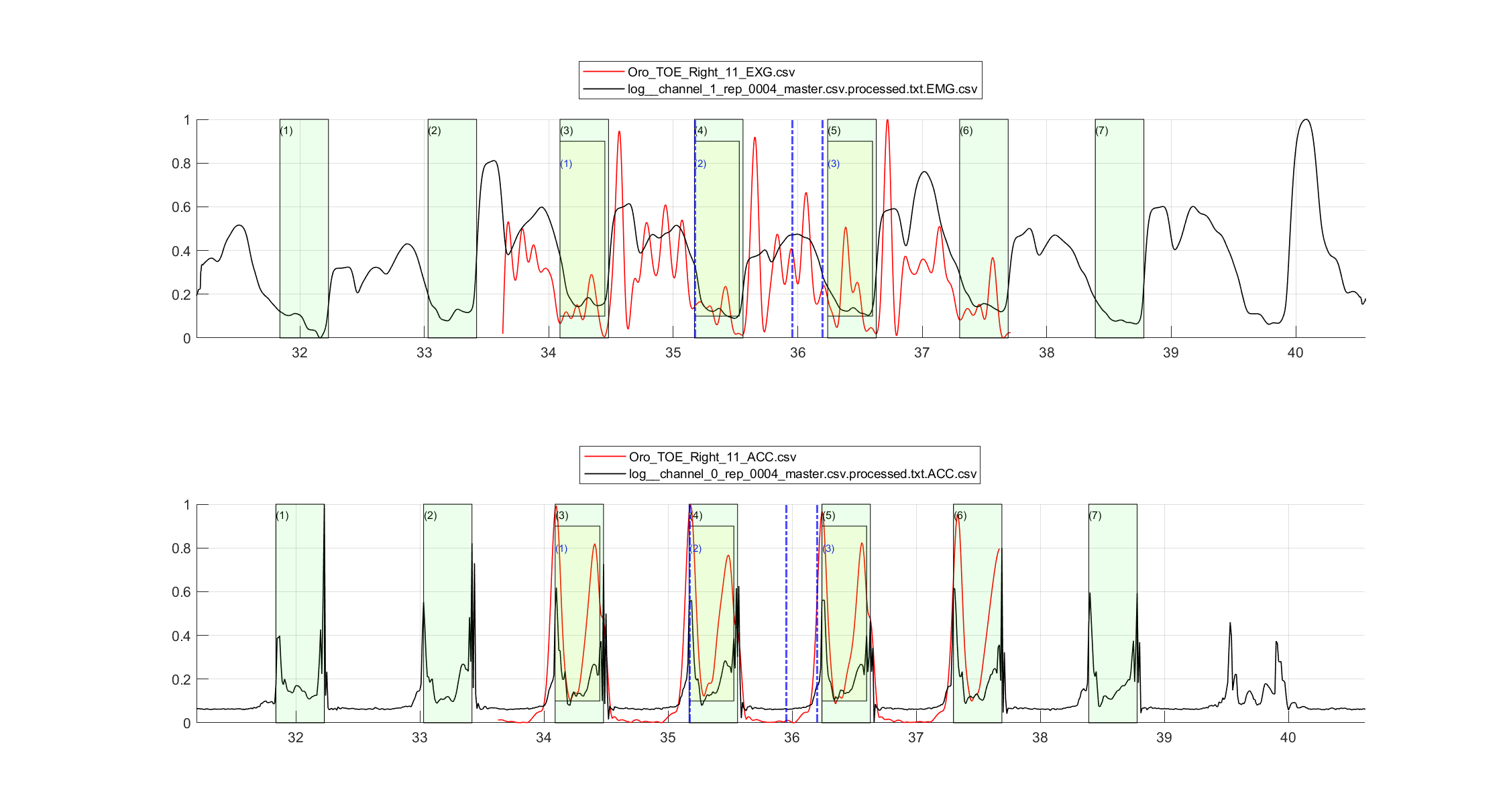

Supplement: Supplementary file 1 [file sensors-22-04957-s001.zip › Part 2 - 3D CGA vs oro sensor system data partitioning/Patient 2-3 shoes/Figure_Oro_TOE_Right_11.png]

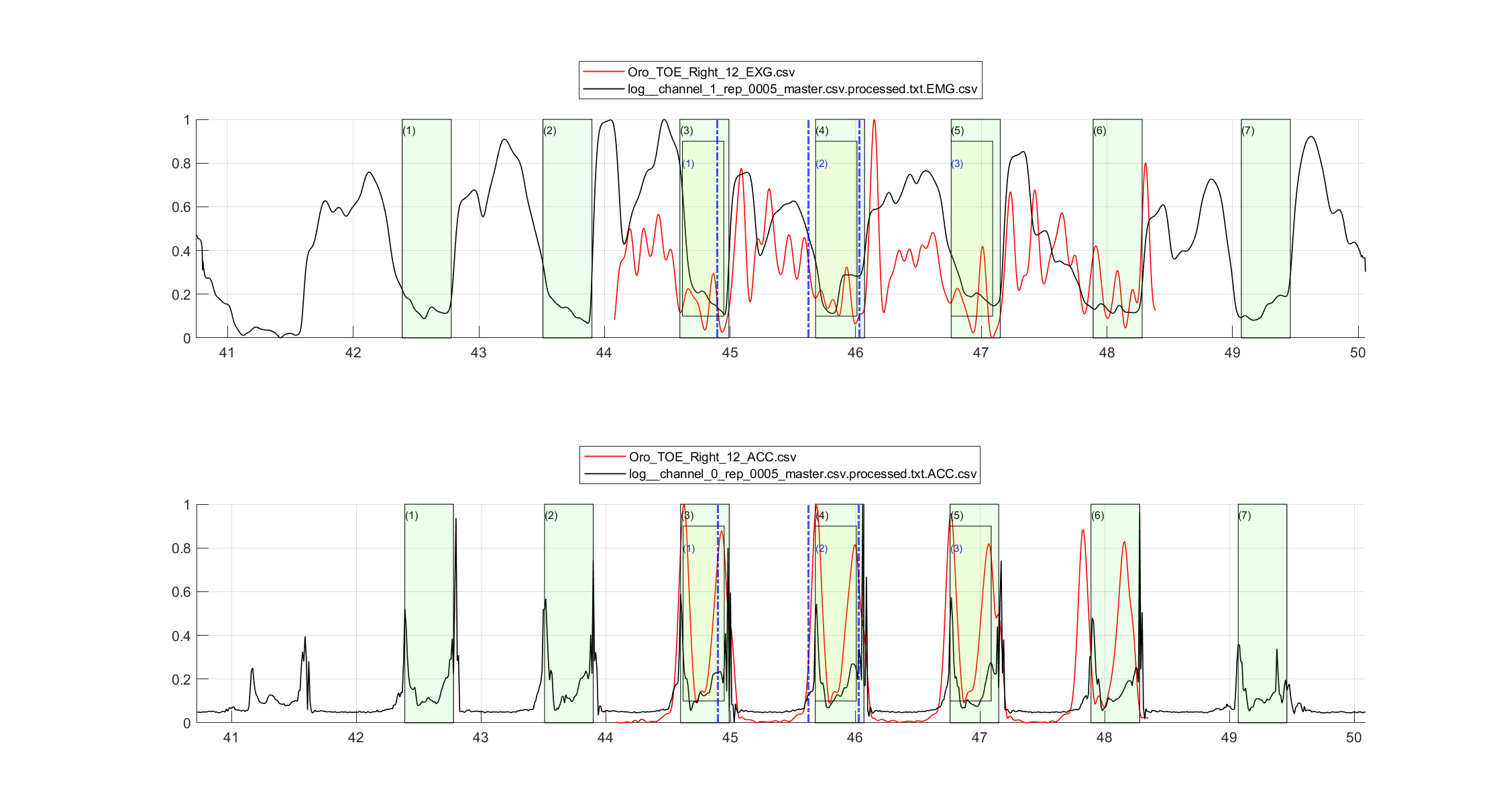

Supplement: Supplementary file 1 [file sensors-22-04957-s001.zip › Part 2 - 3D CGA vs oro sensor system data partitioning/Patient 2-3 shoes/Figure_Oro_TOE_Right_12.png]

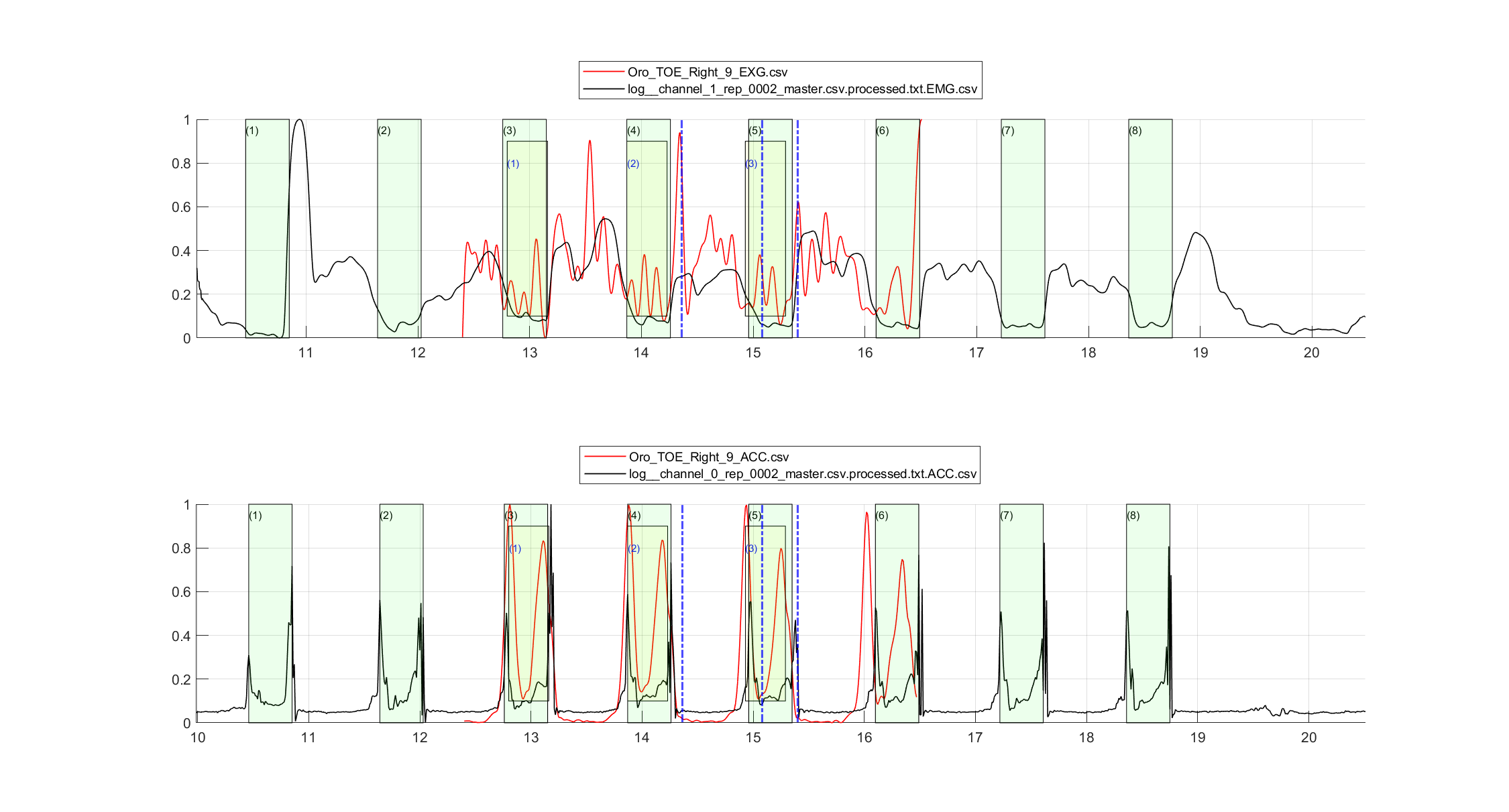

Supplement: Supplementary file 1 [file sensors-22-04957-s001.zip › Part 2 - 3D CGA vs oro sensor system data partitioning/Patient 2-3 shoes/Figure_Oro_TOE_Right_9.png]

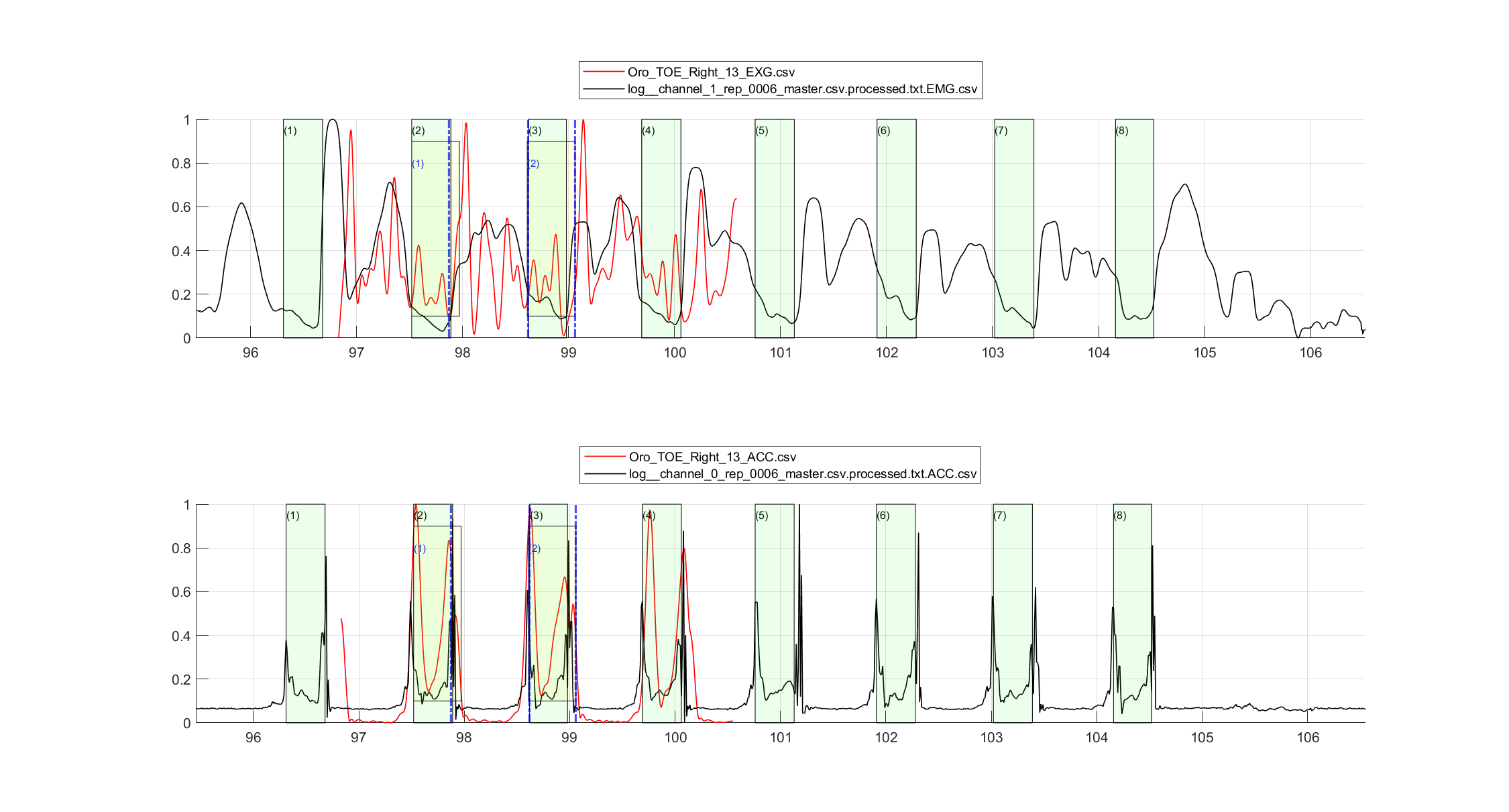

Supplement: Supplementary file 1 [file sensors-22-04957-s001.zip › Part 2 - 3D CGA vs oro sensor system data partitioning/Patient 2-4 shoes 1cm heel/Figure_Oro_TOE_Right_13.png]

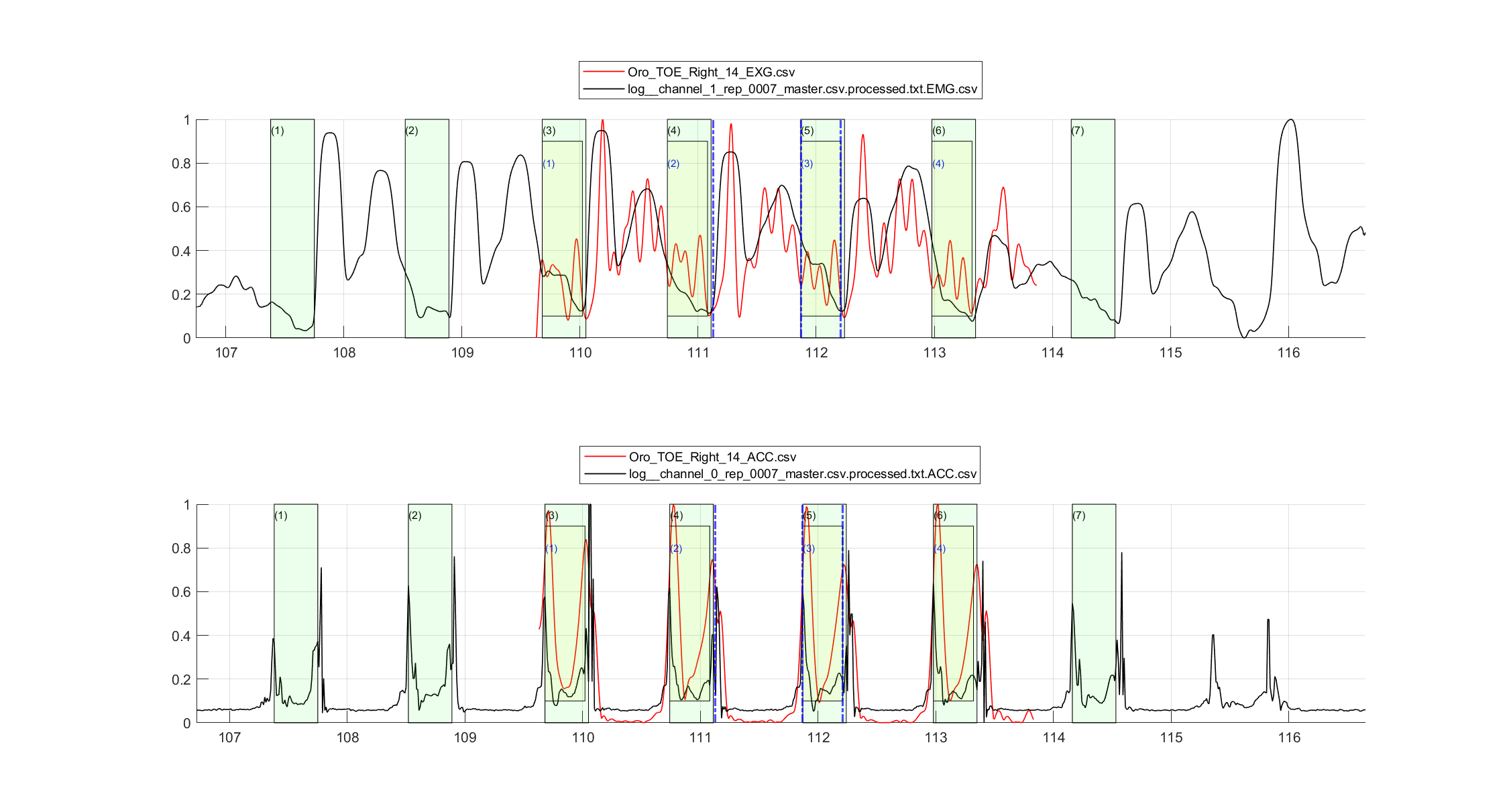

Supplement: Supplementary file 1 [file sensors-22-04957-s001.zip › Part 2 - 3D CGA vs oro sensor system data partitioning/Patient 2-4 shoes 1cm heel/Figure_Oro_TOE_Right_14.png]

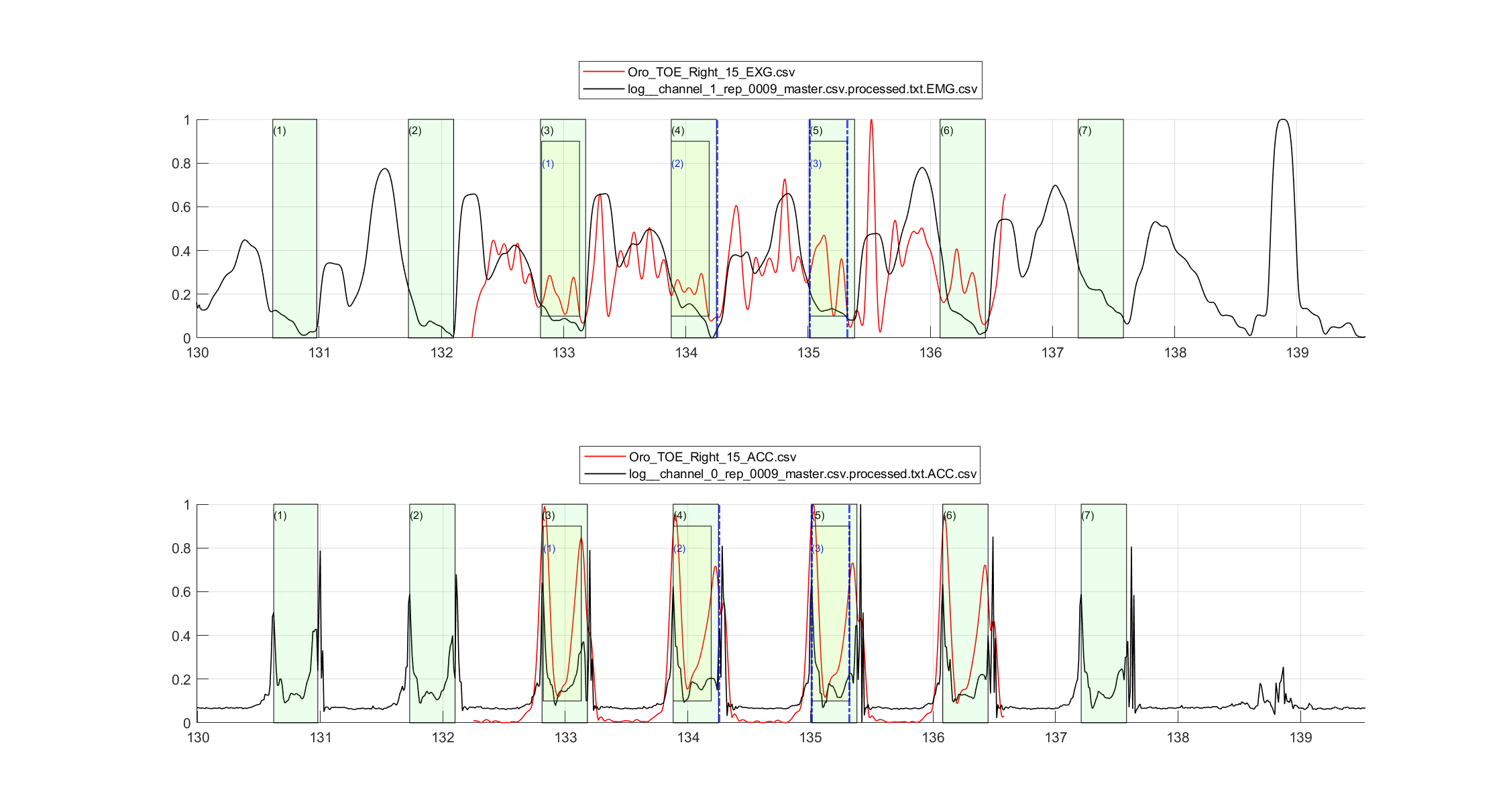

Supplement: Supplementary file 1 [file sensors-22-04957-s001.zip › Part 2 - 3D CGA vs oro sensor system data partitioning/Patient 2-4 shoes 1cm heel/Figure_Oro_TOE_Right_15.png]

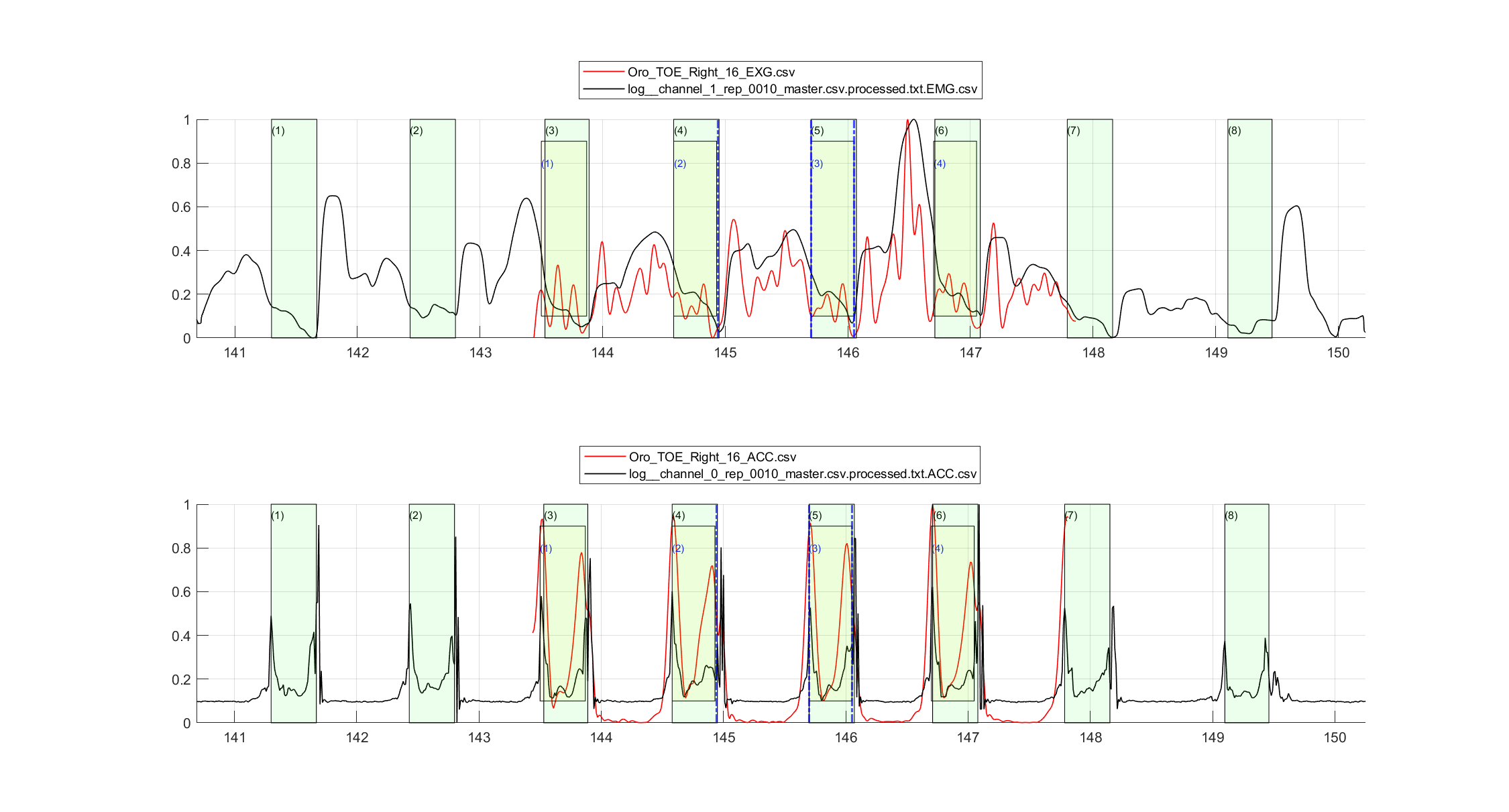

Supplement: Supplementary file 1 [file sensors-22-04957-s001.zip › Part 2 - 3D CGA vs oro sensor system data partitioning/Patient 2-4 shoes 1cm heel/Figure_Oro_TOE_Right_16.png]

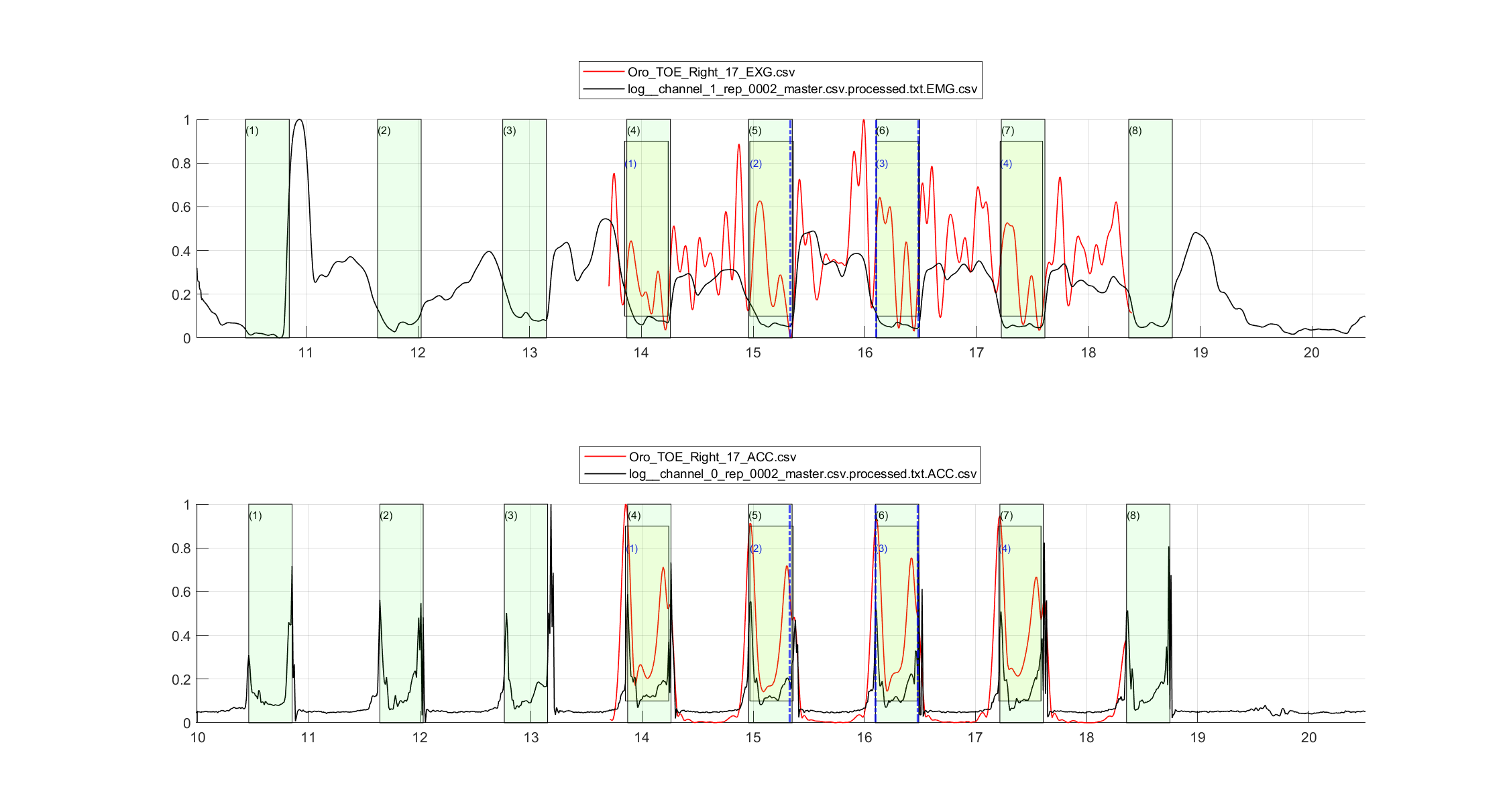

Supplement: Supplementary file 1 [file sensors-22-04957-s001.zip › Part 2 - 3D CGA vs oro sensor system data partitioning/Patient 2-5 orthotic shoes/Figure_Oro_TOE_Right_17.png]

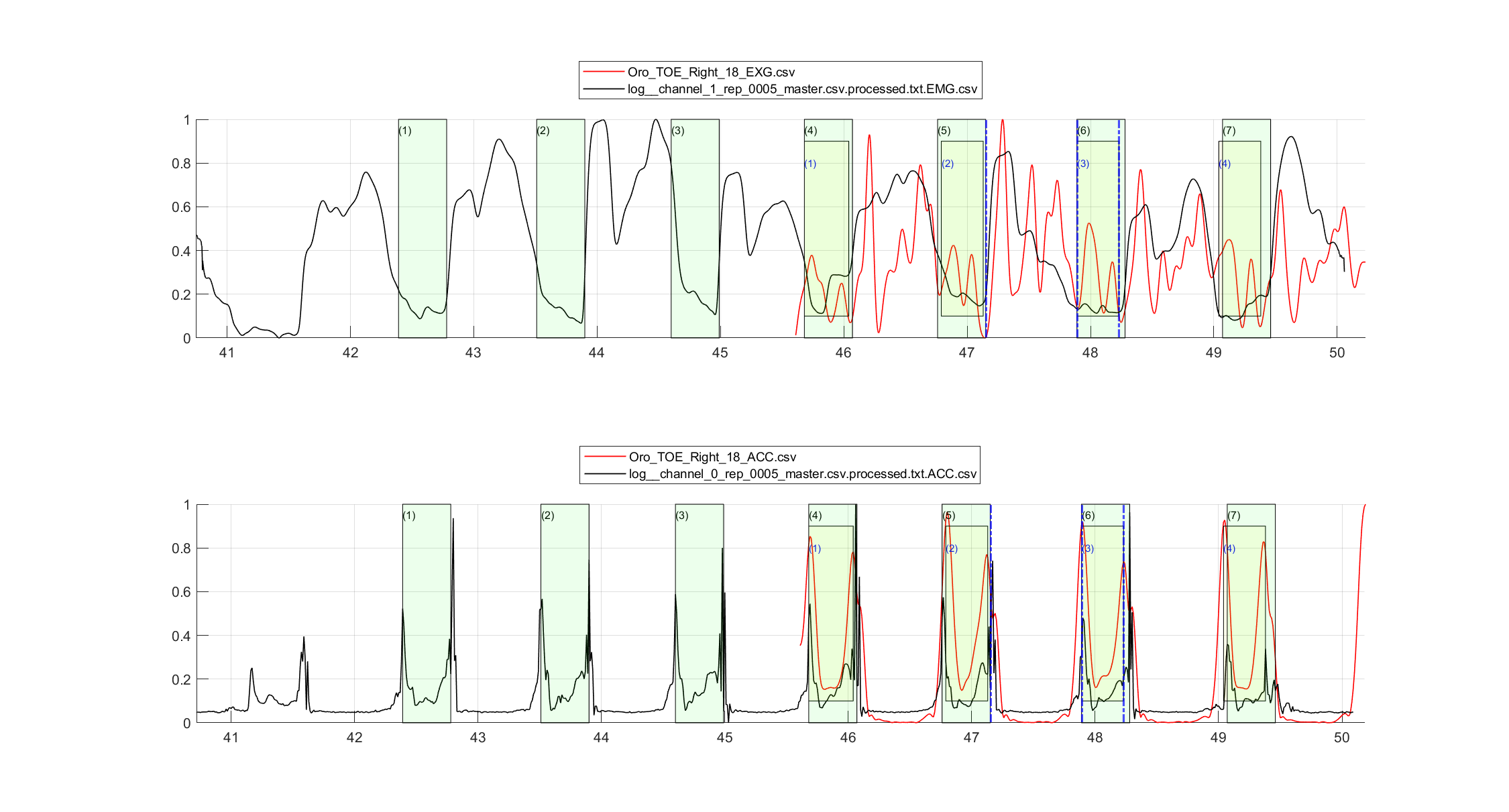

Supplement: Supplementary file 1 [file sensors-22-04957-s001.zip › Part 2 - 3D CGA vs oro sensor system data partitioning/Patient 2-5 orthotic shoes/Figure_Oro_TOE_Right_18.png]

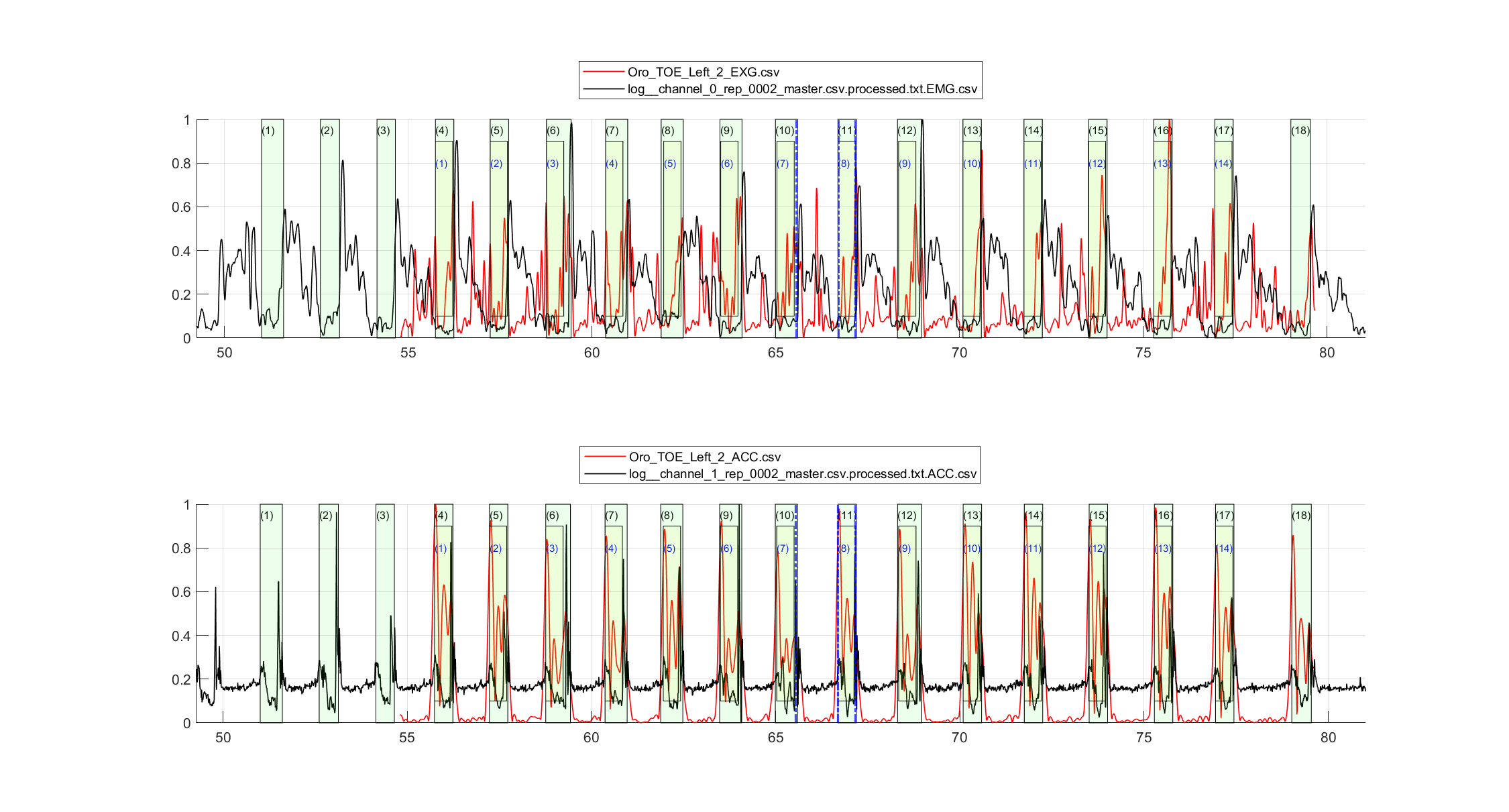

Supplement: Supplementary file 1 [file sensors-22-04957-s001.zip › Part 2 - 3D CGA vs oro sensor system data partitioning/Patient 3-1 barefoot/Figure_Oro_TOE_Left_2.png]

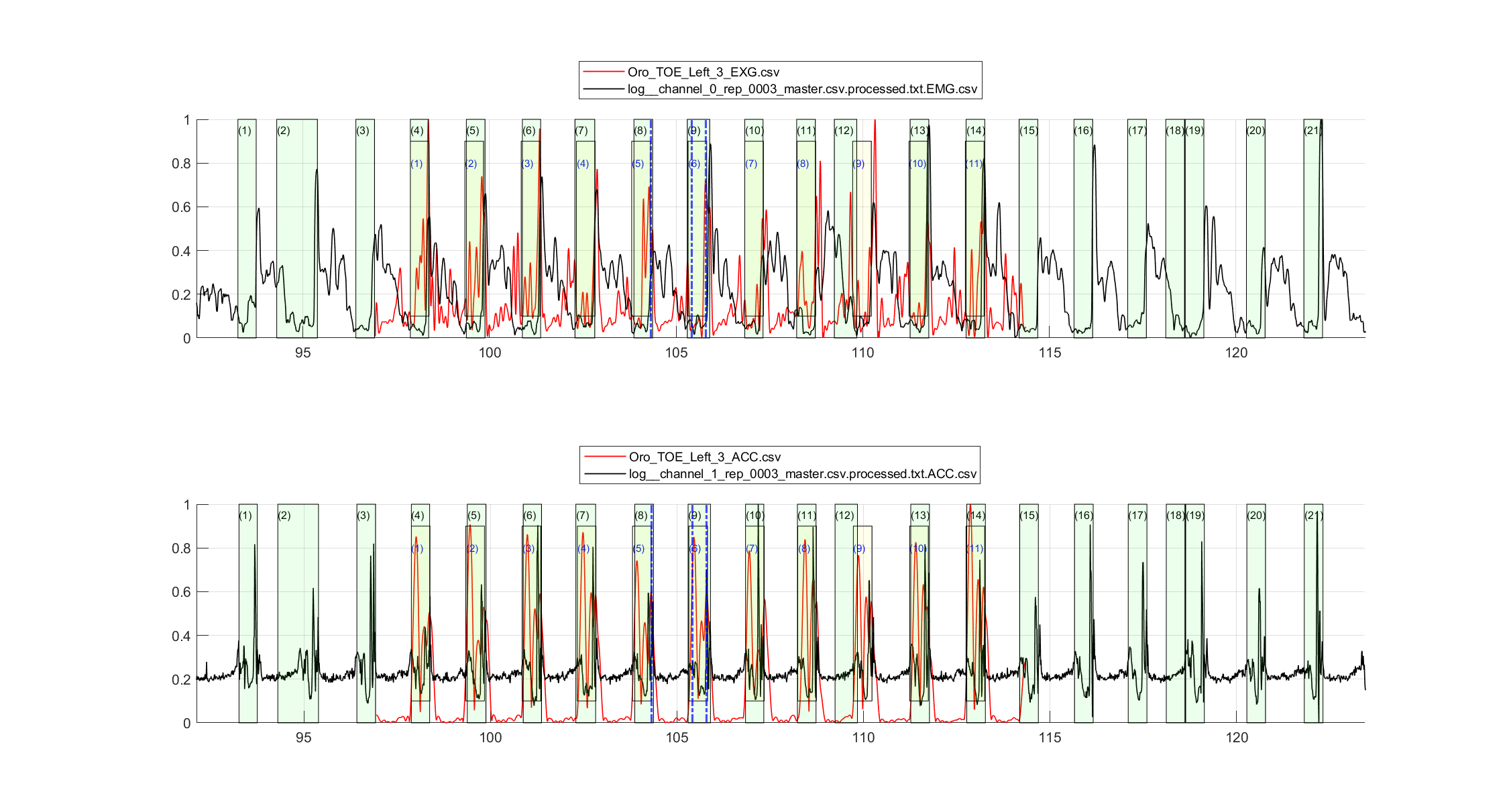

Supplement: Supplementary file 1 [file sensors-22-04957-s001.zip › Part 2 - 3D CGA vs oro sensor system data partitioning/Patient 3-1 barefoot/Figure_Oro_TOE_Left_3.png]

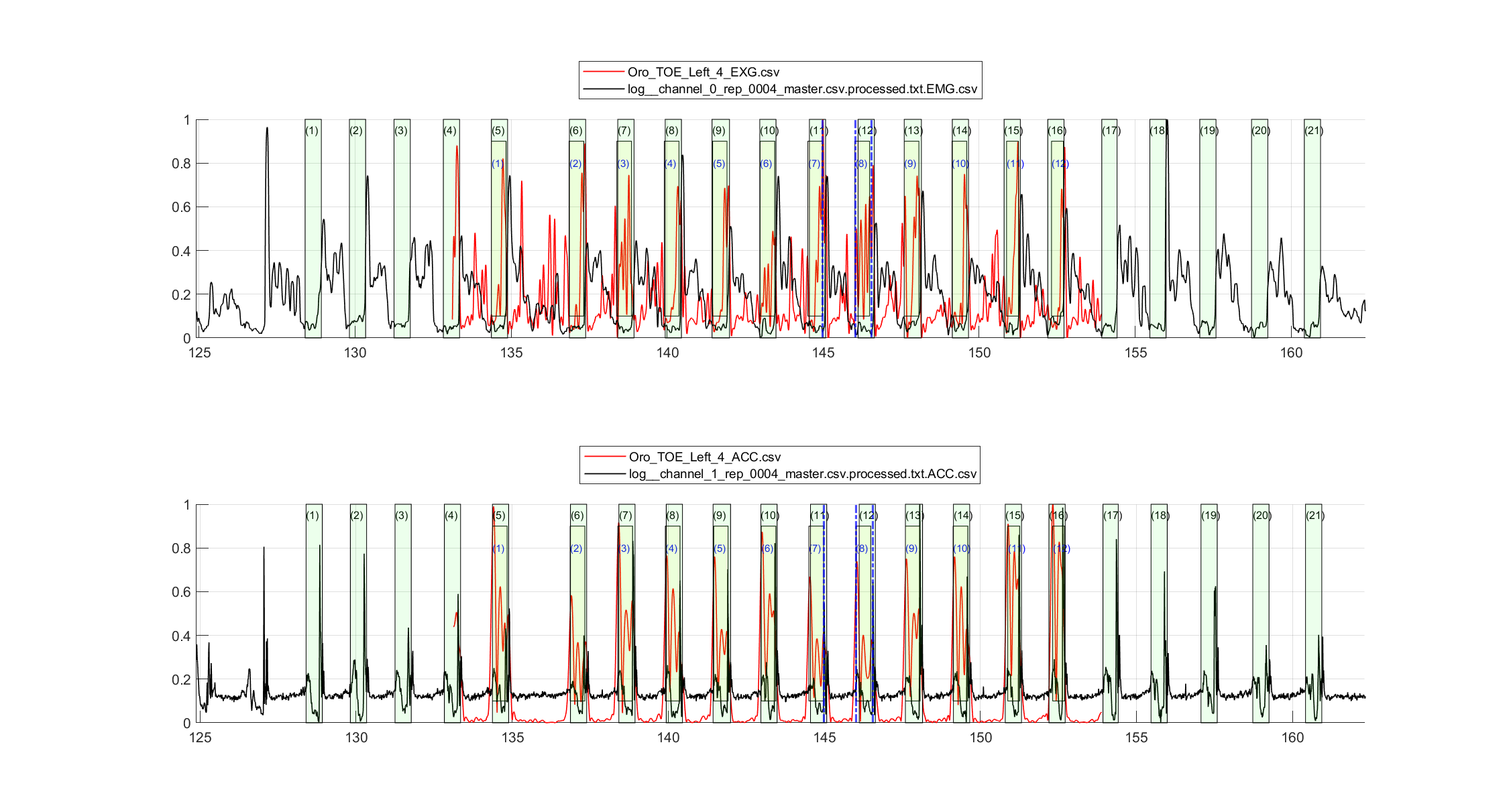

Supplement: Supplementary file 1 [file sensors-22-04957-s001.zip › Part 2 - 3D CGA vs oro sensor system data partitioning/Patient 3-1 barefoot/Figure_Oro_TOE_Left_4.png]

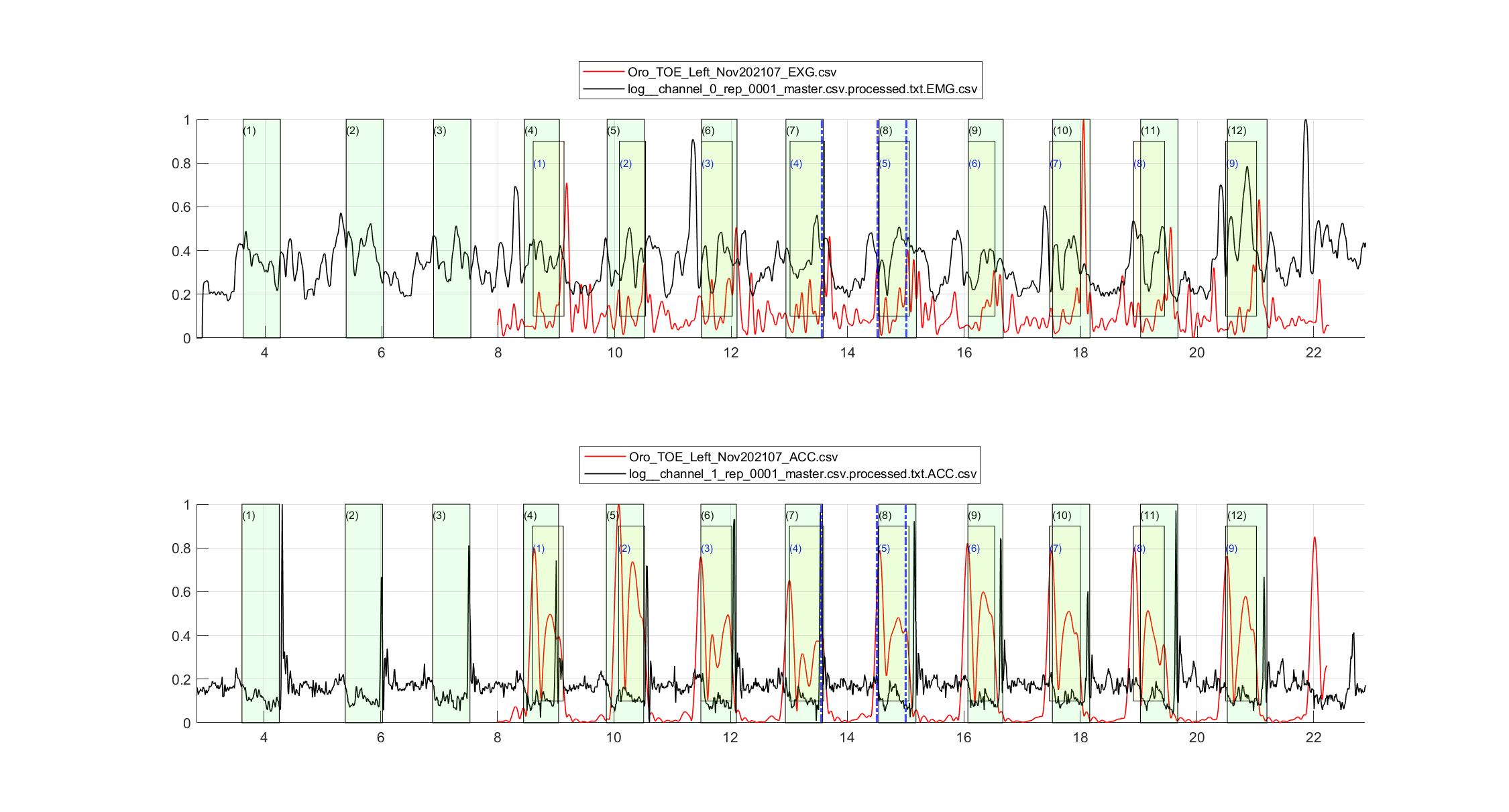

Supplement: Supplementary file 1 [file sensors-22-04957-s001.zip › Part 2 - 3D CGA vs oro sensor system data partitioning/Patient 3-2 shoes/Figure_Oro_TOE_Left_Nov202107.png]

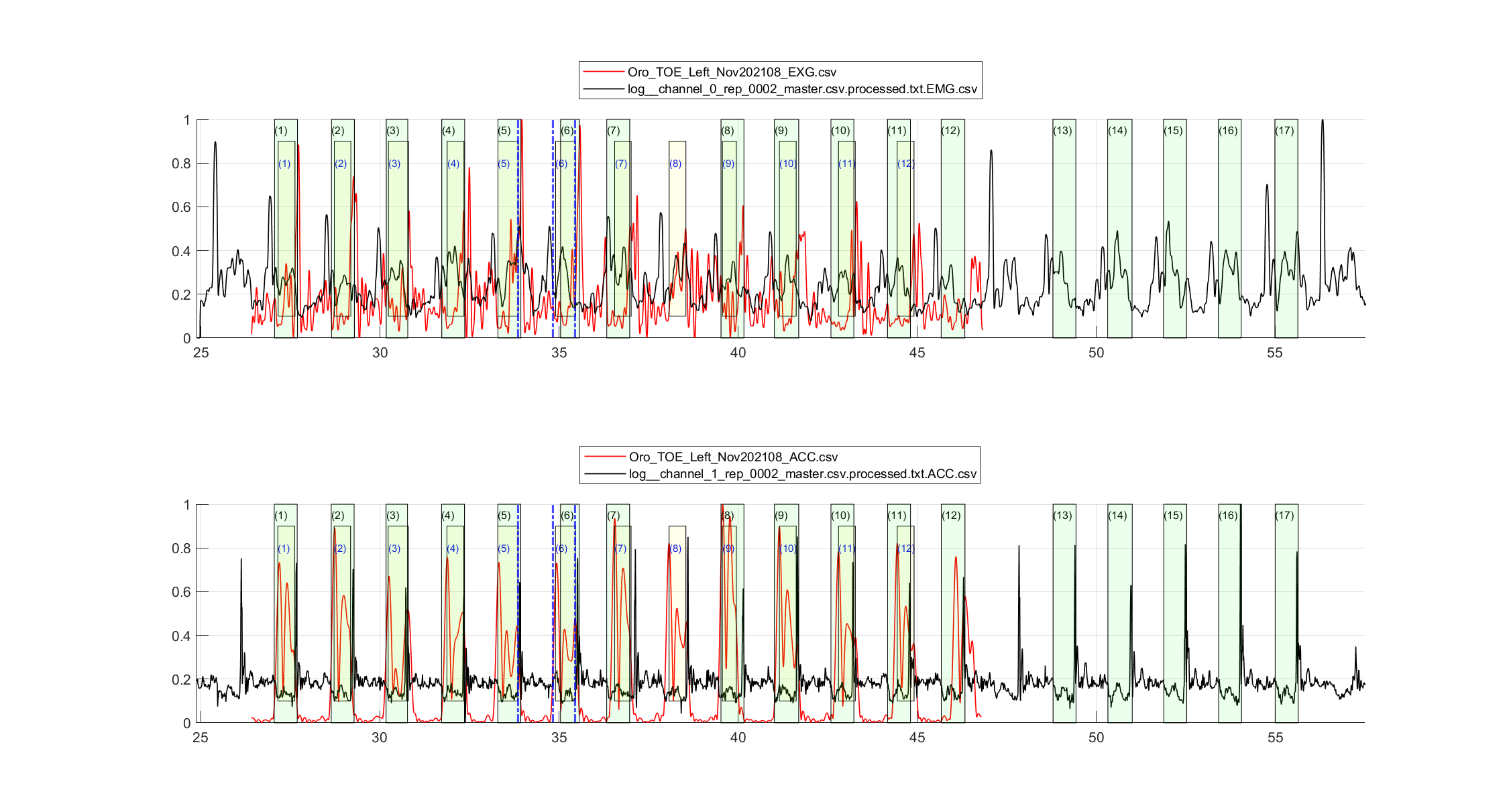

Supplement: Supplementary file 1 [file sensors-22-04957-s001.zip › Part 2 - 3D CGA vs oro sensor system data partitioning/Patient 3-2 shoes/Figure_Oro_TOE_Left_Nov202108.png]

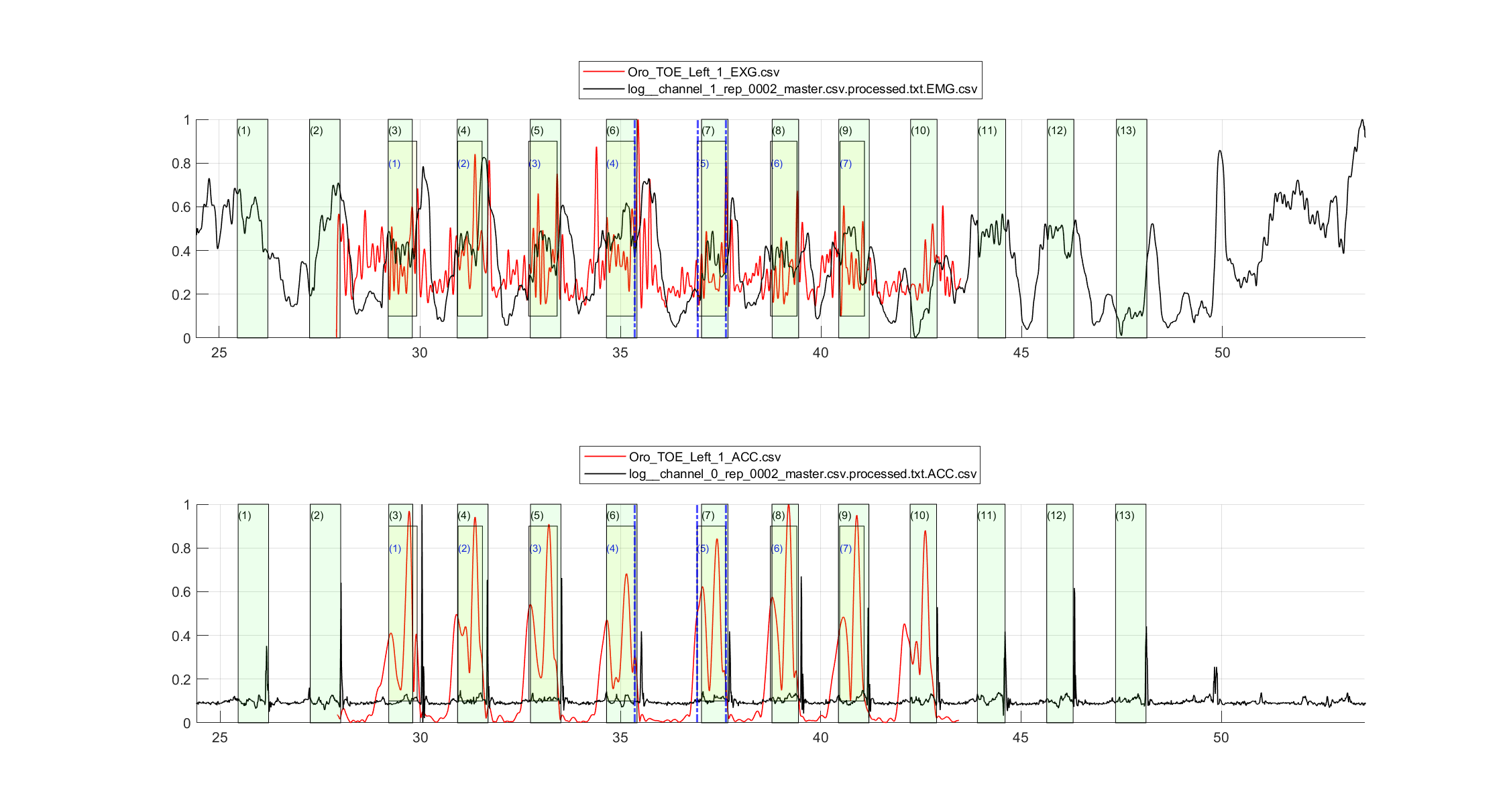

Supplement: Supplementary file 1 [file sensors-22-04957-s001.zip › Part 2 - 3D CGA vs oro sensor system data partitioning/Patient 4-1 barefoot/Figure_Oro_TOE_Left_1.png]

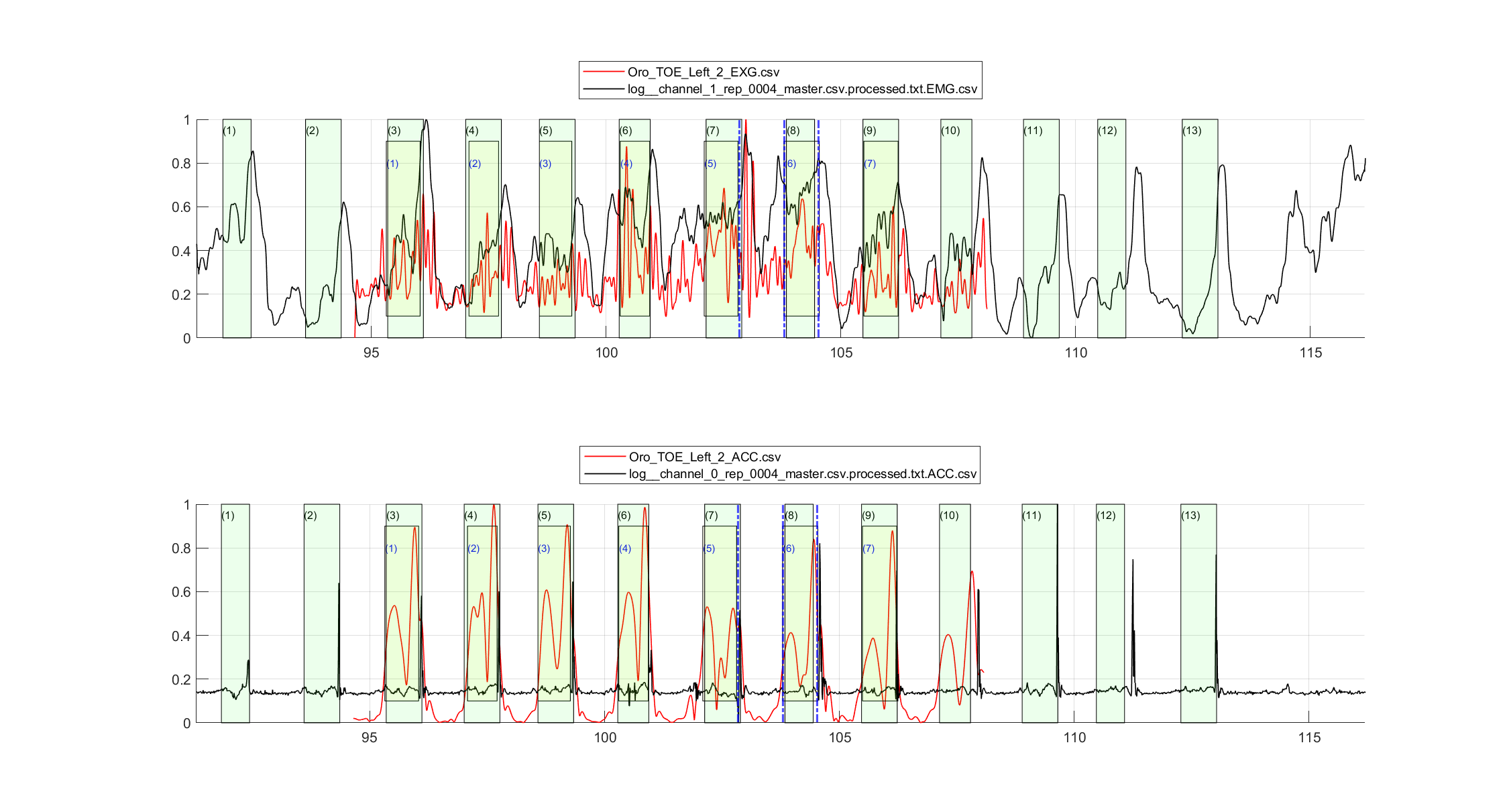

Supplement: Supplementary file 1 [file sensors-22-04957-s001.zip › Part 2 - 3D CGA vs oro sensor system data partitioning/Patient 4-1 barefoot/Figure_Oro_TOE_Left_2.png]

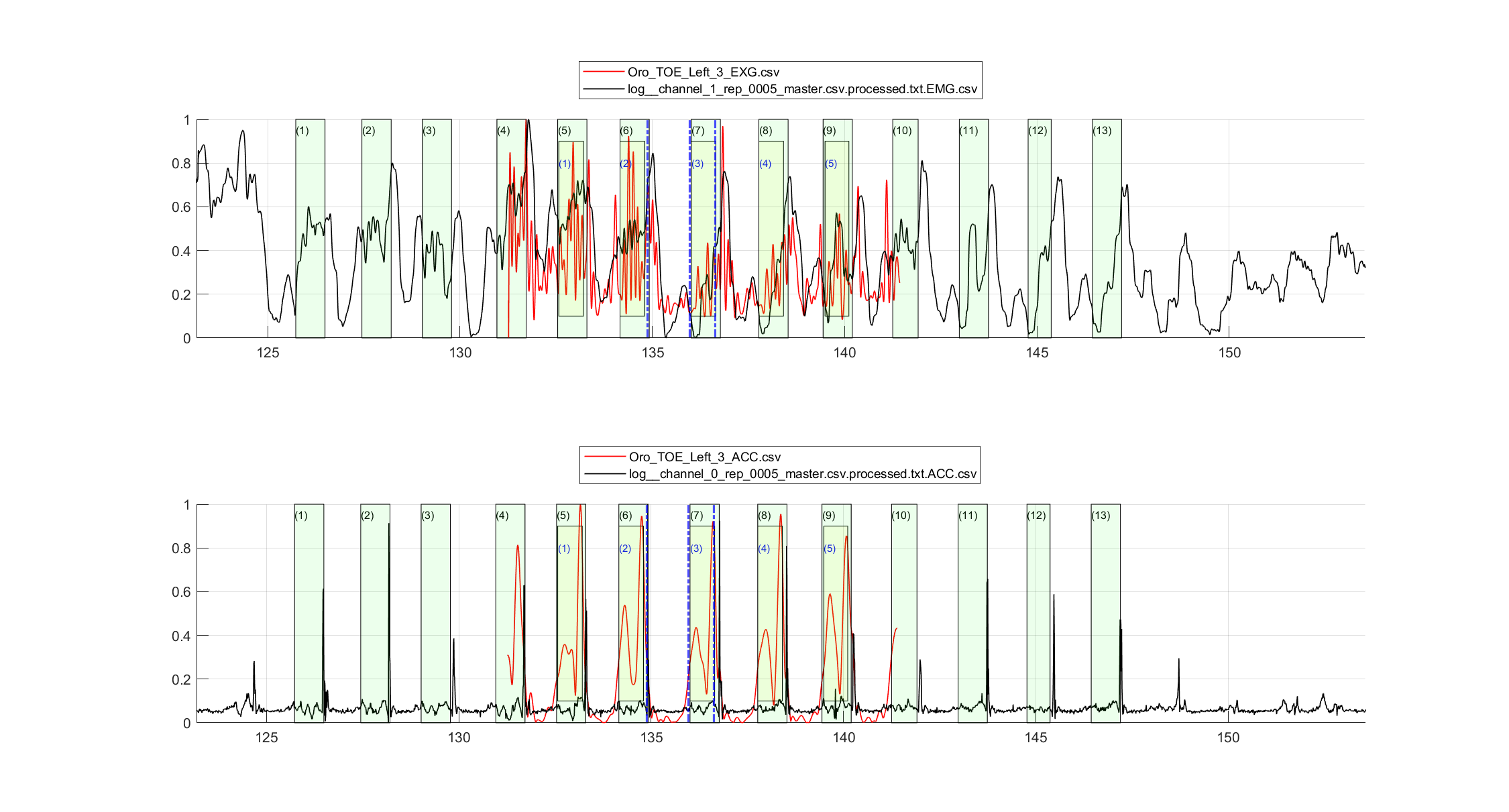

Supplement: Supplementary file 1 [file sensors-22-04957-s001.zip › Part 2 - 3D CGA vs oro sensor system data partitioning/Patient 4-1 barefoot/Figure_Oro_TOE_Left_3.png]

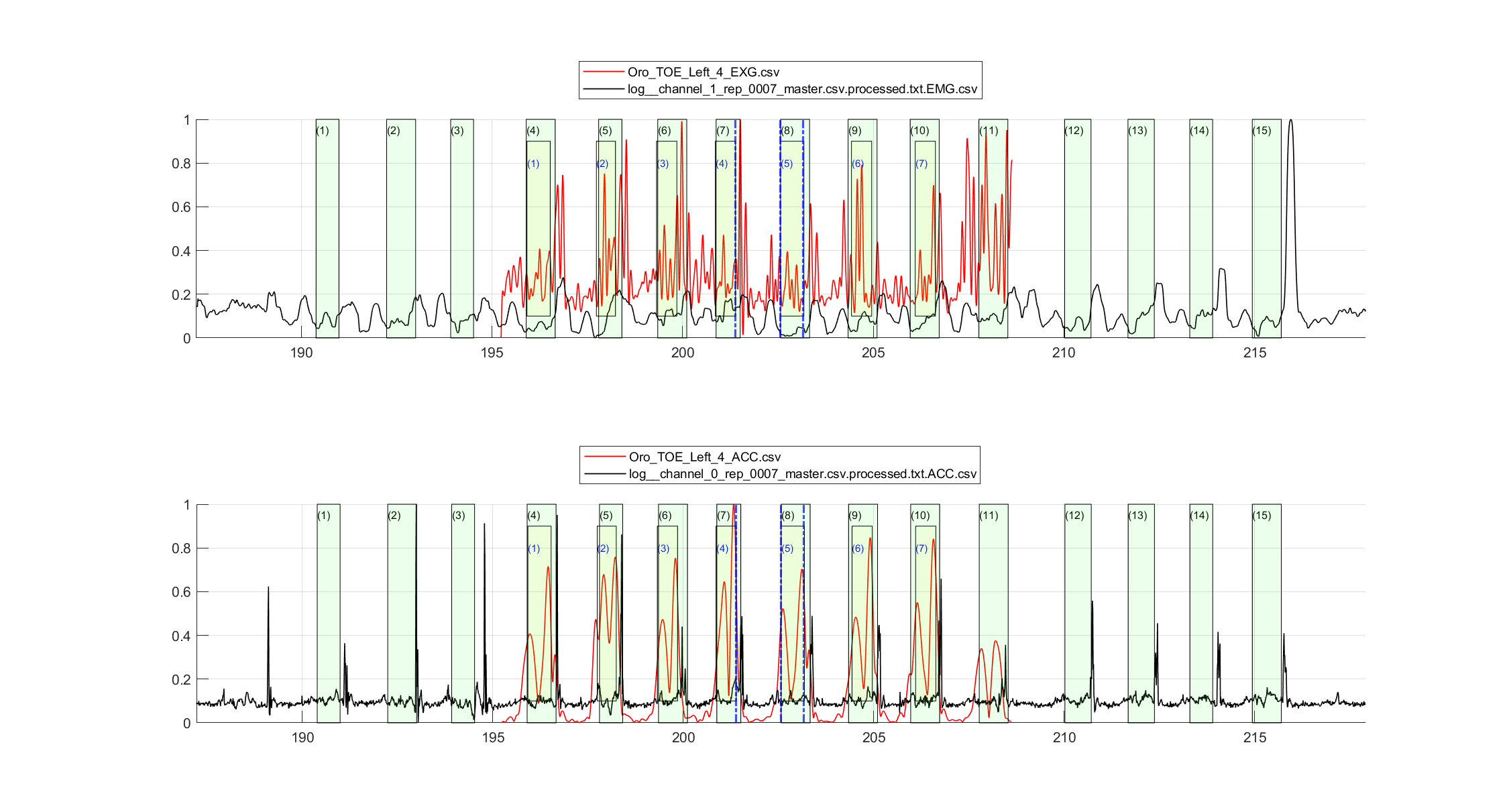

Supplement: Supplementary file 1 [file sensors-22-04957-s001.zip › Part 2 - 3D CGA vs oro sensor system data partitioning/Patient 4-1 barefoot/Figure_Oro_TOE_Left_4.png]

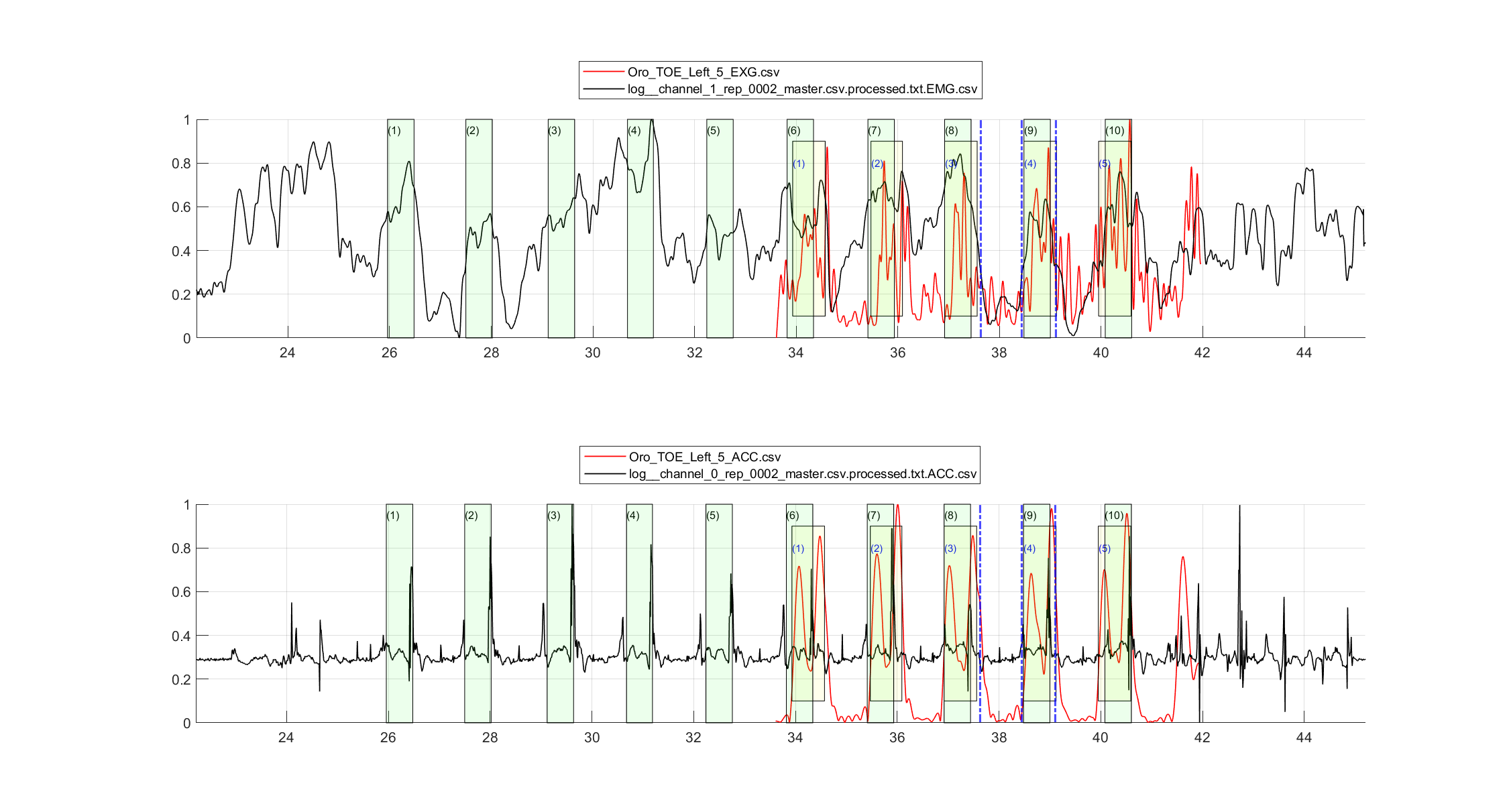

Supplement: Supplementary file 1 [file sensors-22-04957-s001.zip › Part 2 - 3D CGA vs oro sensor system data partitioning/Patient 4-2 shoes/Figure_Oro_TOE_Left_5.png]

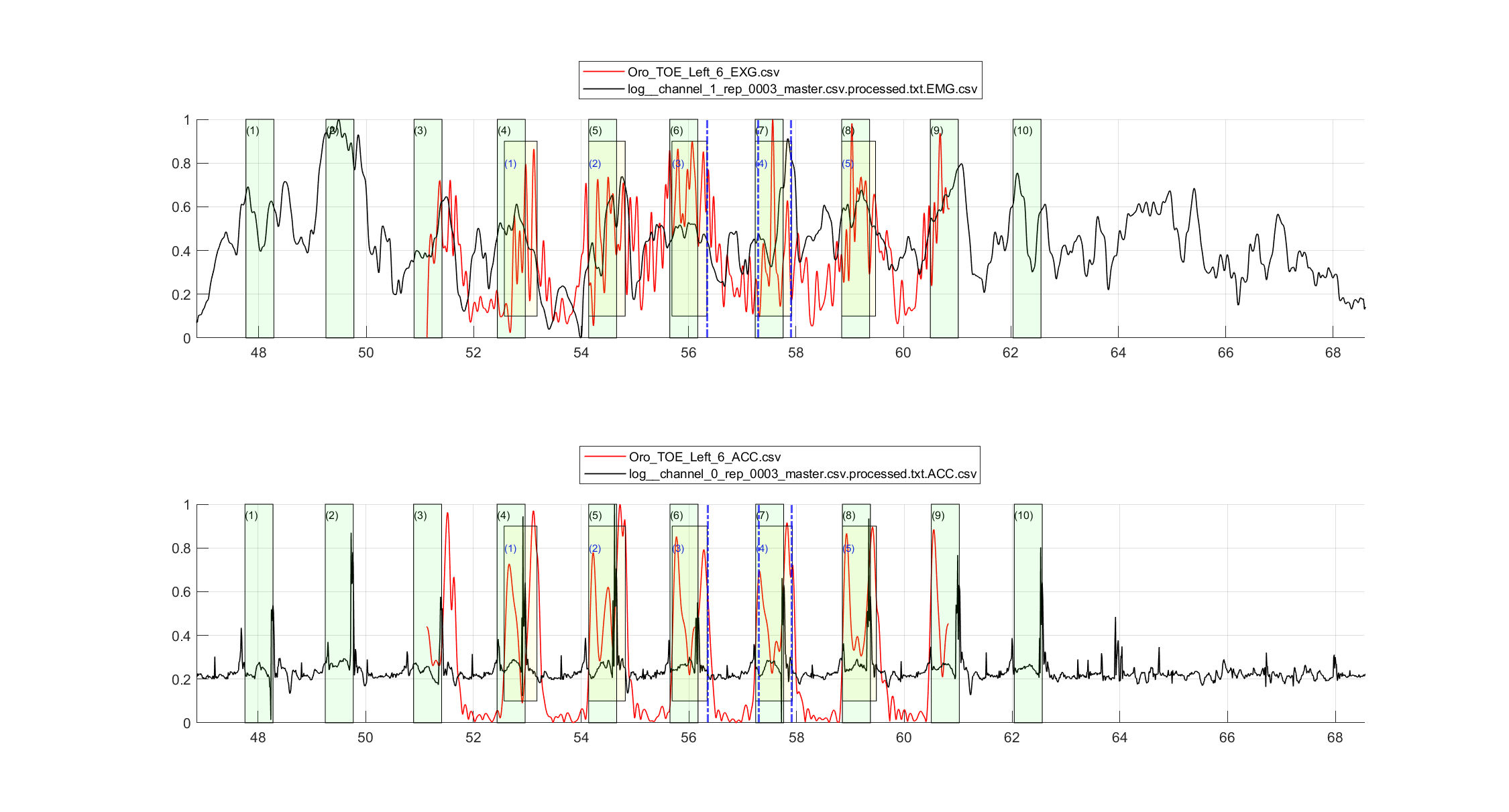

Supplement: Supplementary file 1 [file sensors-22-04957-s001.zip › Part 2 - 3D CGA vs oro sensor system data partitioning/Patient 4-2 shoes/Figure_Oro_TOE_Left_6.png]

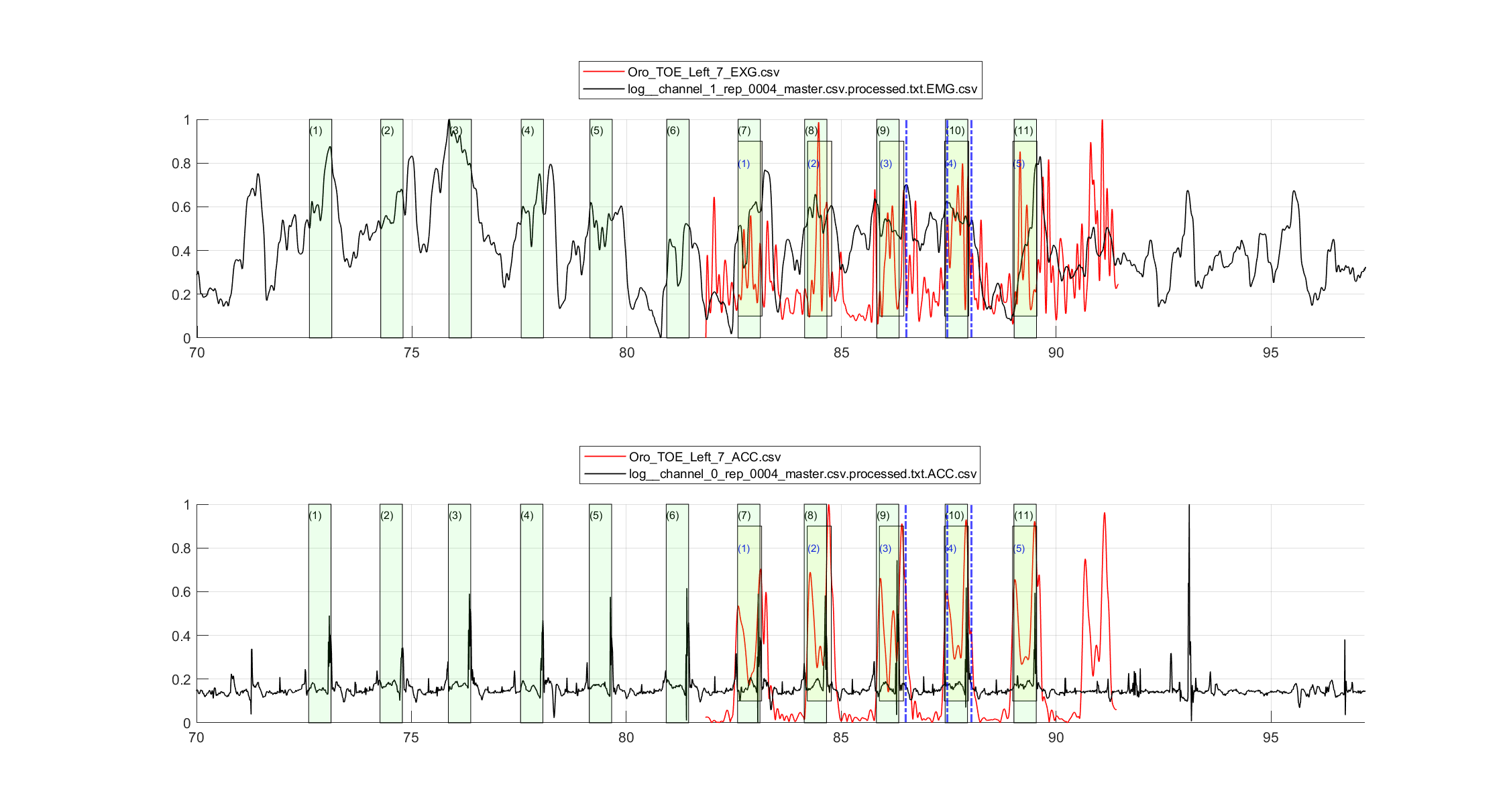

Supplement: Supplementary file 1 [file sensors-22-04957-s001.zip › Part 2 - 3D CGA vs oro sensor system data partitioning/Patient 4-2 shoes/Figure_Oro_TOE_Left_7.png]

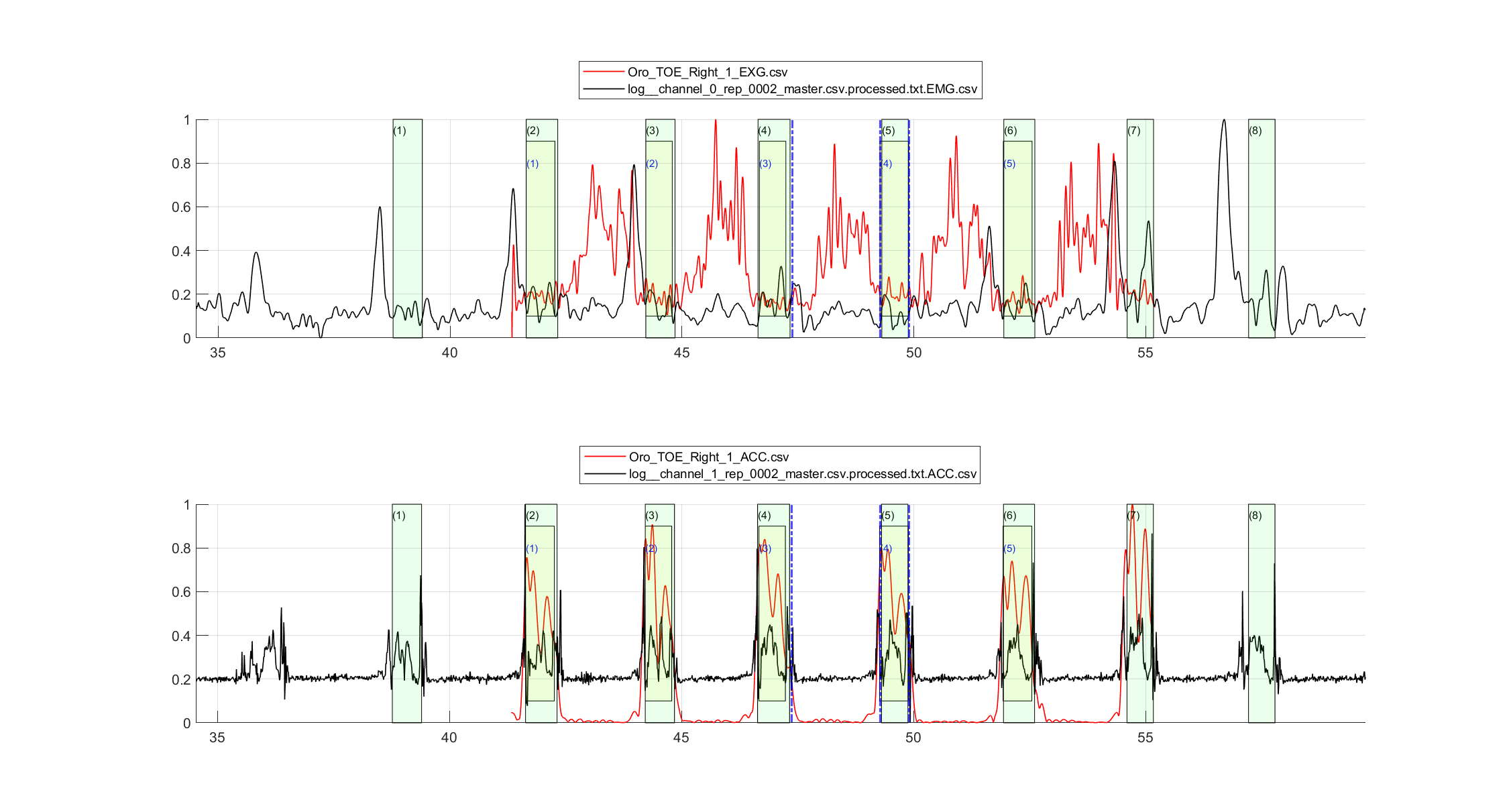

Supplement: Supplementary file 1 [file sensors-22-04957-s001.zip › Part 2 - 3D CGA vs oro sensor system data partitioning/Patient 5-1 barefoot/Figure_Oro_TOE_Right_1.png]

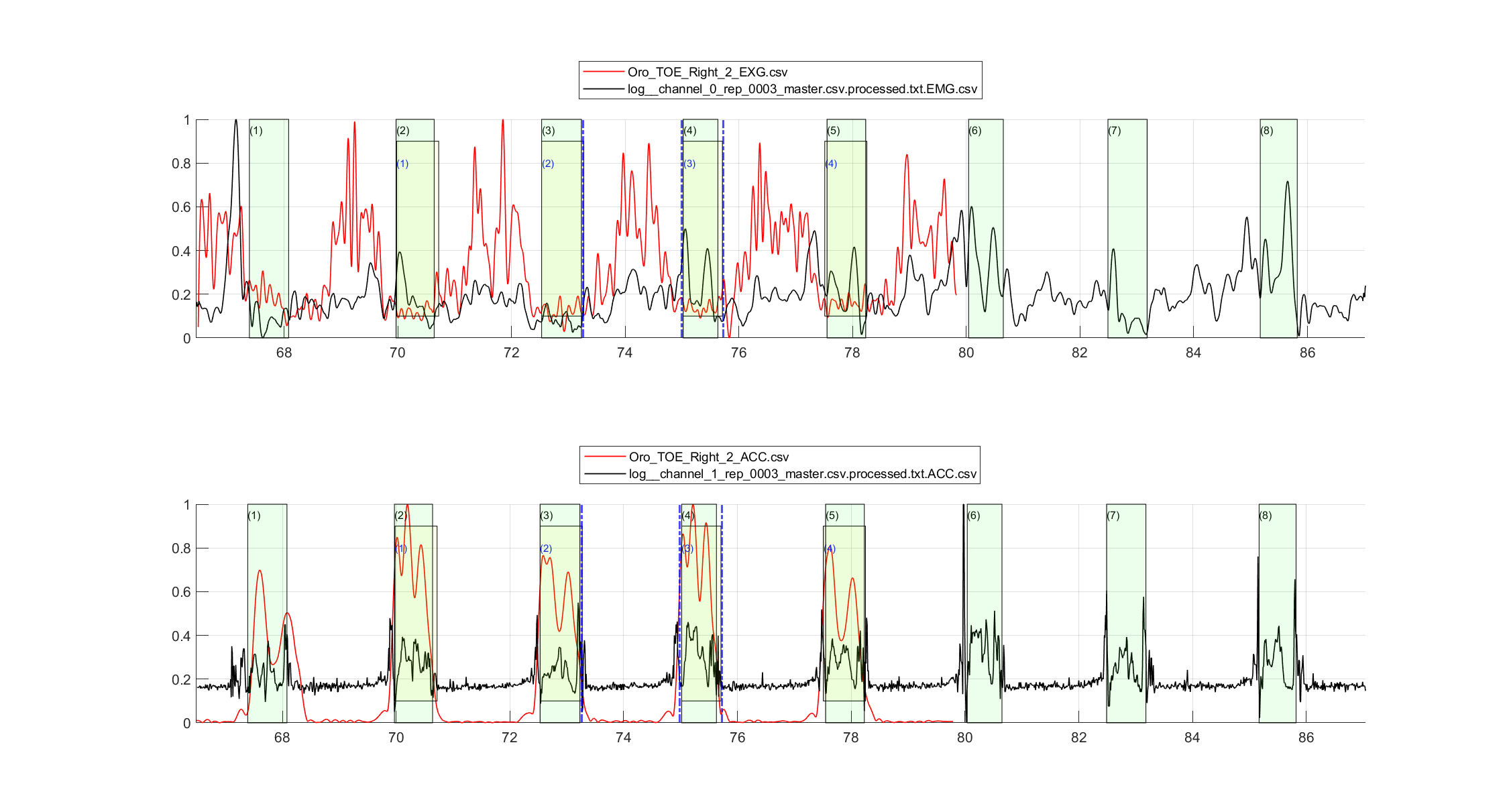

Supplement: Supplementary file 1 [file sensors-22-04957-s001.zip › Part 2 - 3D CGA vs oro sensor system data partitioning/Patient 5-1 barefoot/Figure_Oro_TOE_Right_2.png]

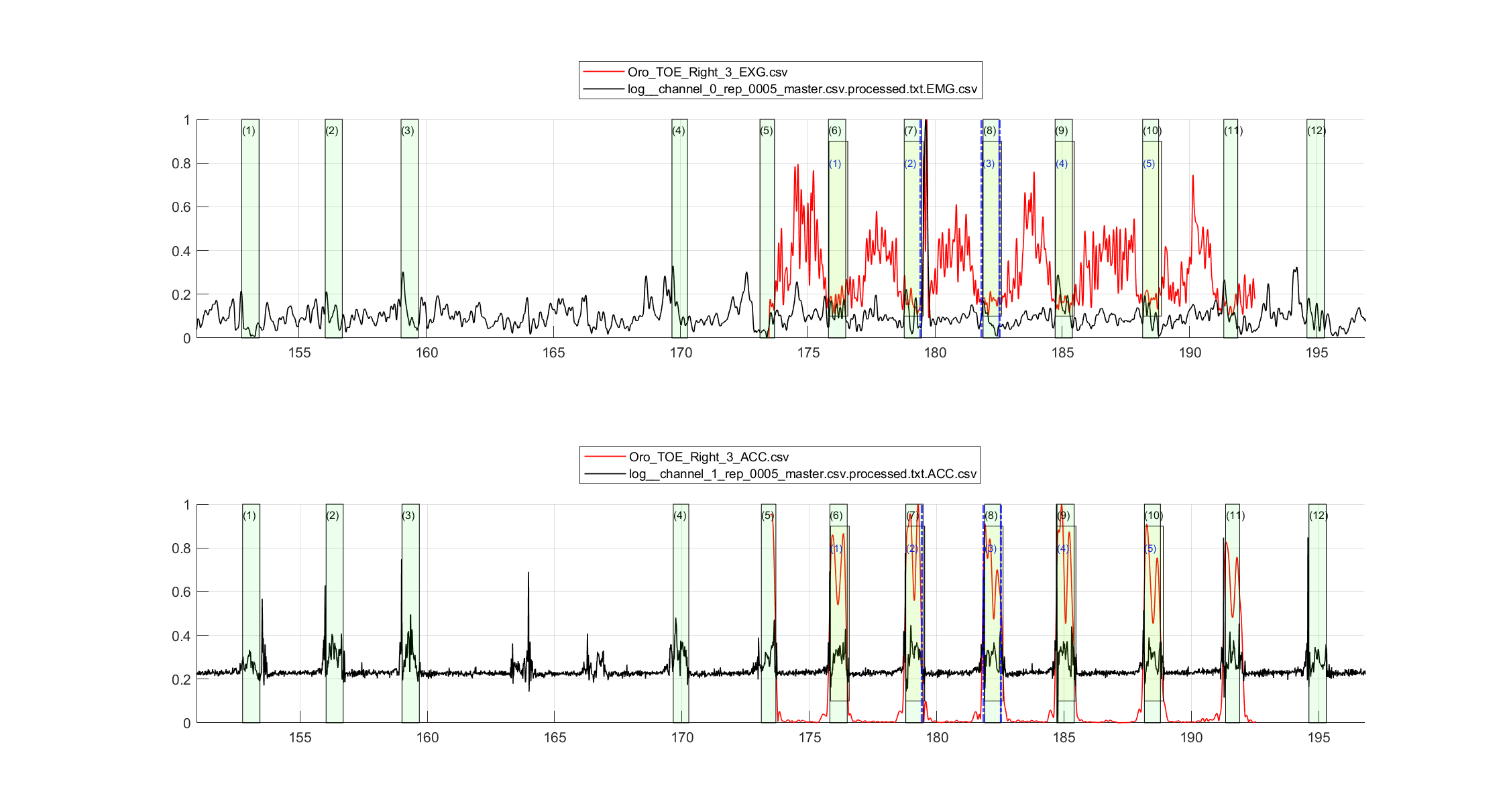

Supplement: Supplementary file 1 [file sensors-22-04957-s001.zip › Part 2 - 3D CGA vs oro sensor system data partitioning/Patient 5-1 barefoot/Figure_Oro_TOE_Right_3.png]

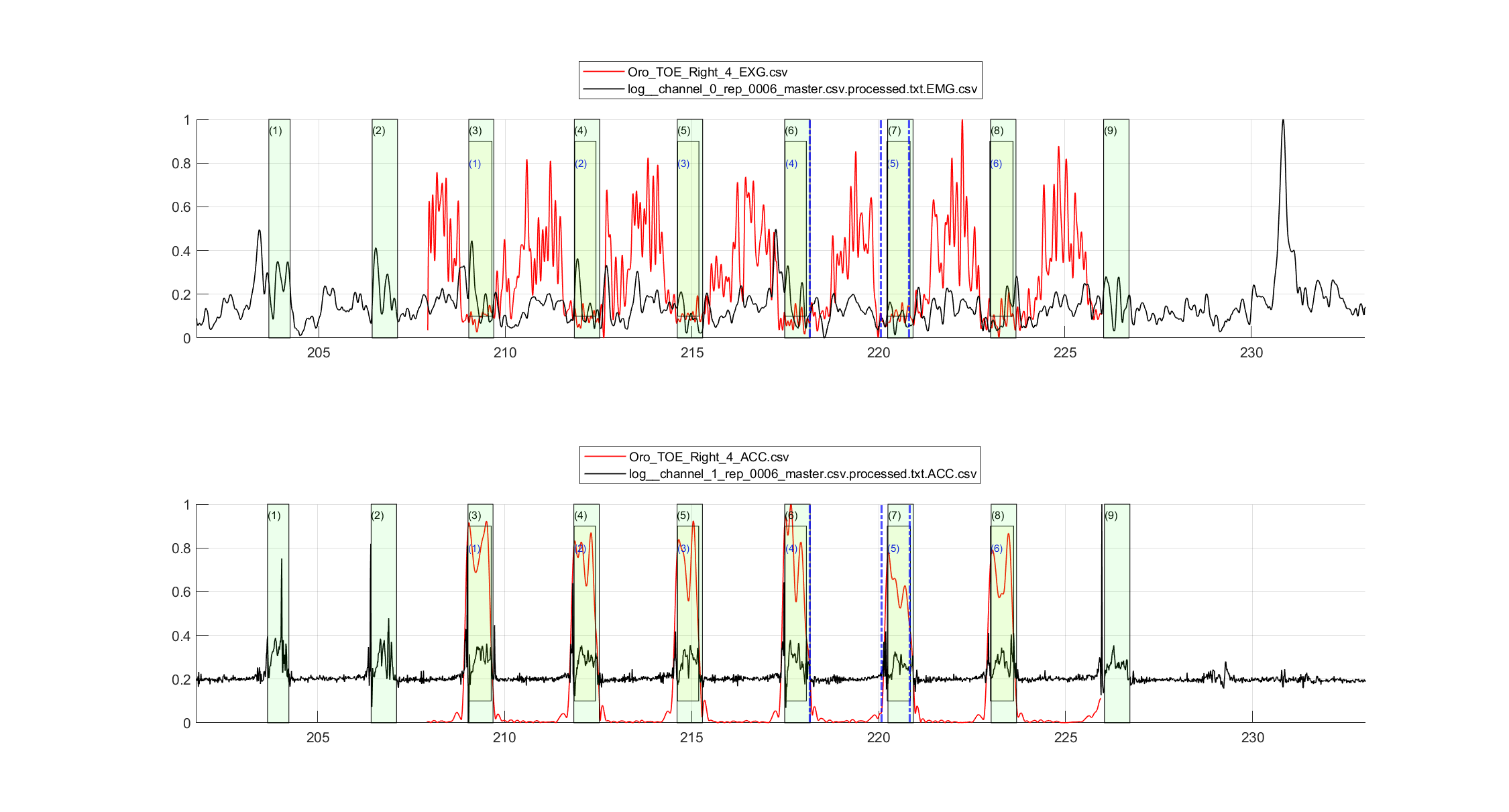

Supplement: Supplementary file 1 [file sensors-22-04957-s001.zip › Part 2 - 3D CGA vs oro sensor system data partitioning/Patient 5-1 barefoot/Figure_Oro_TOE_Right_4.png]

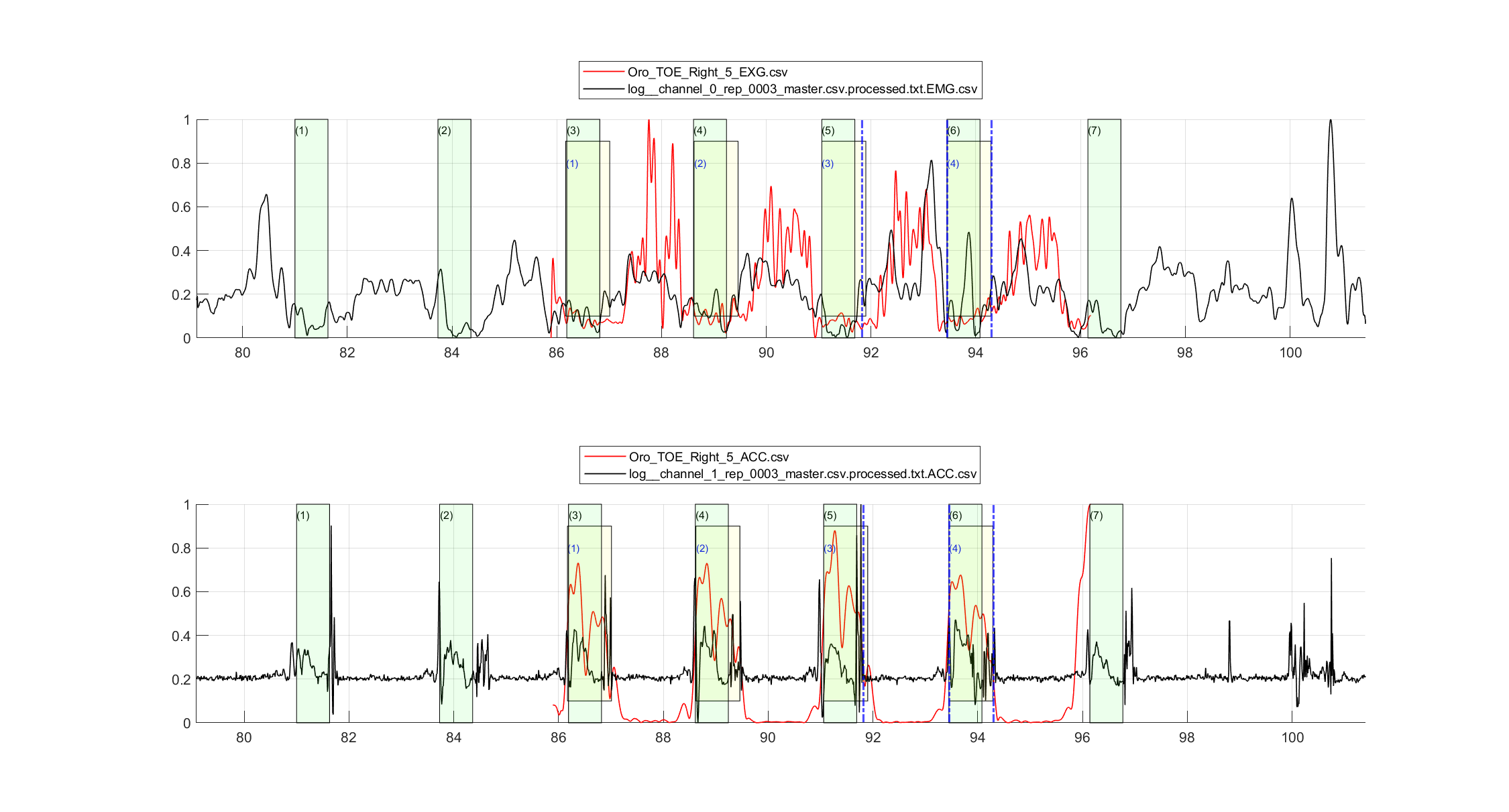

Supplement: Supplementary file 1 [file sensors-22-04957-s001.zip › Part 2 - 3D CGA vs oro sensor system data partitioning/Patient 5-2 shoes/Figure_Oro_TOE_Right_5.png]

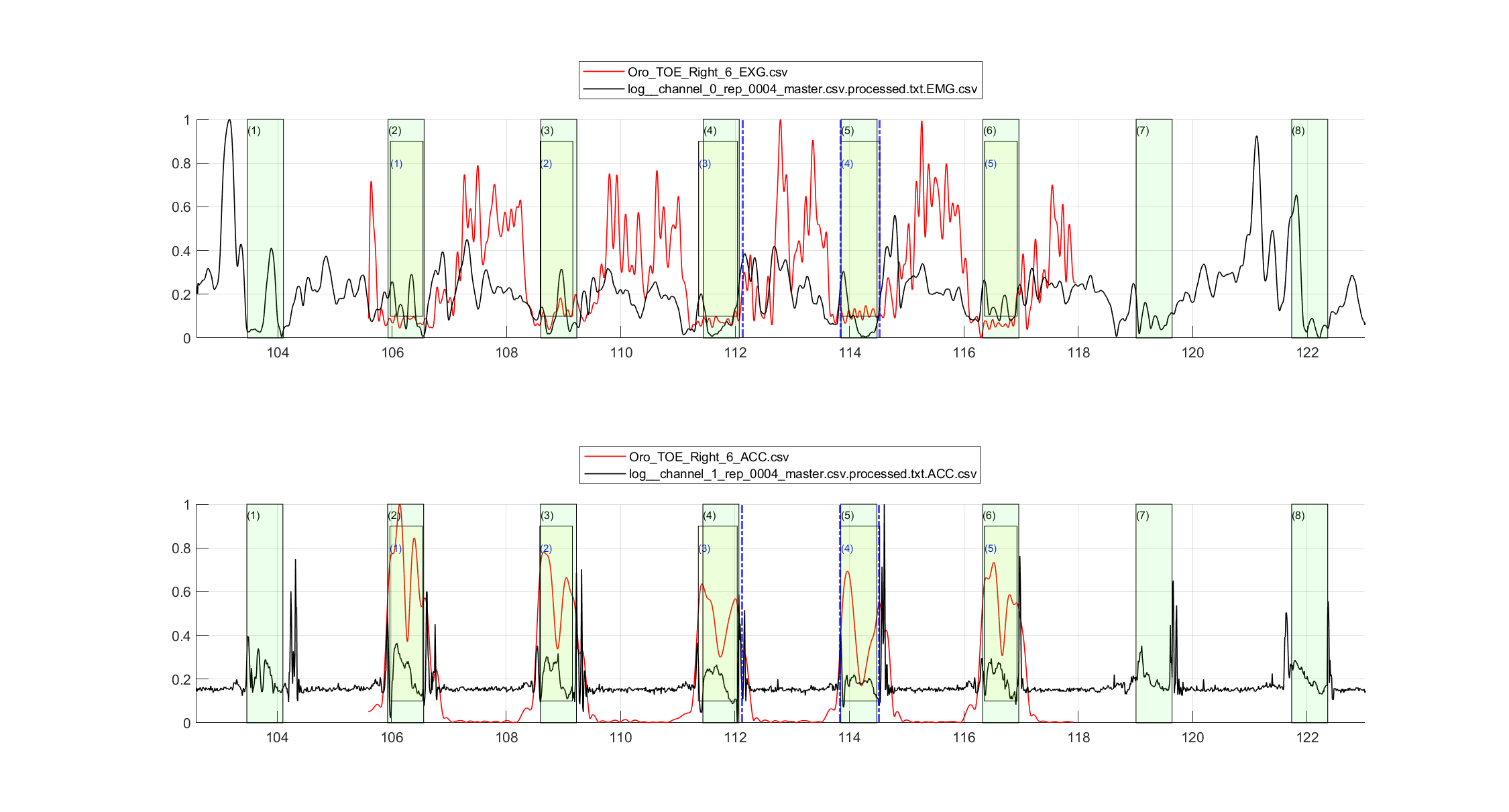

Supplement: Supplementary file 1 [file sensors-22-04957-s001.zip › Part 2 - 3D CGA vs oro sensor system data partitioning/Patient 5-2 shoes/Figure_Oro_TOE_Right_6.png]

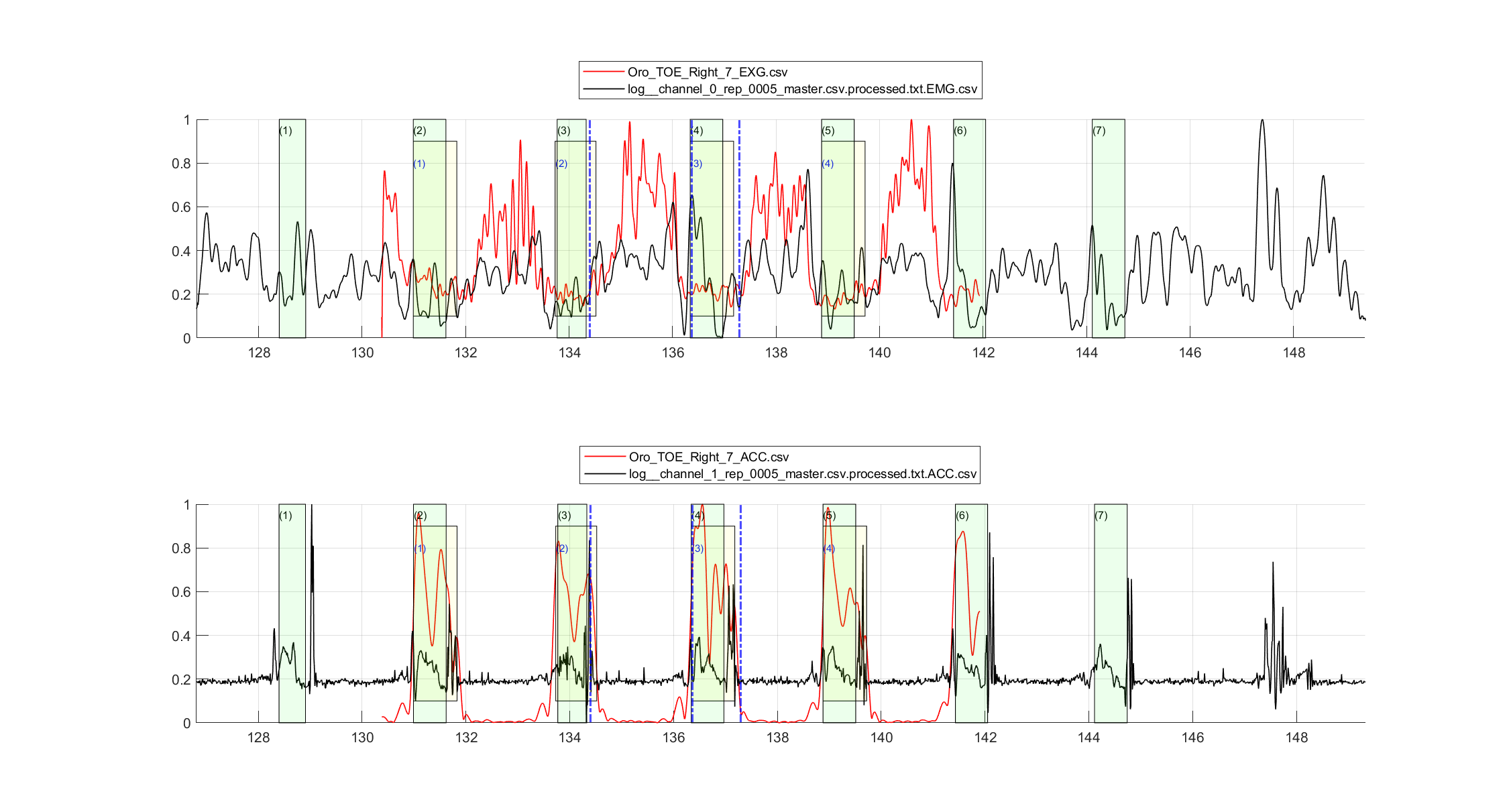

Supplement: Supplementary file 1 [file sensors-22-04957-s001.zip › Part 2 - 3D CGA vs oro sensor system data partitioning/Patient 5-2 shoes/Figure_Oro_TOE_Right_7.png]

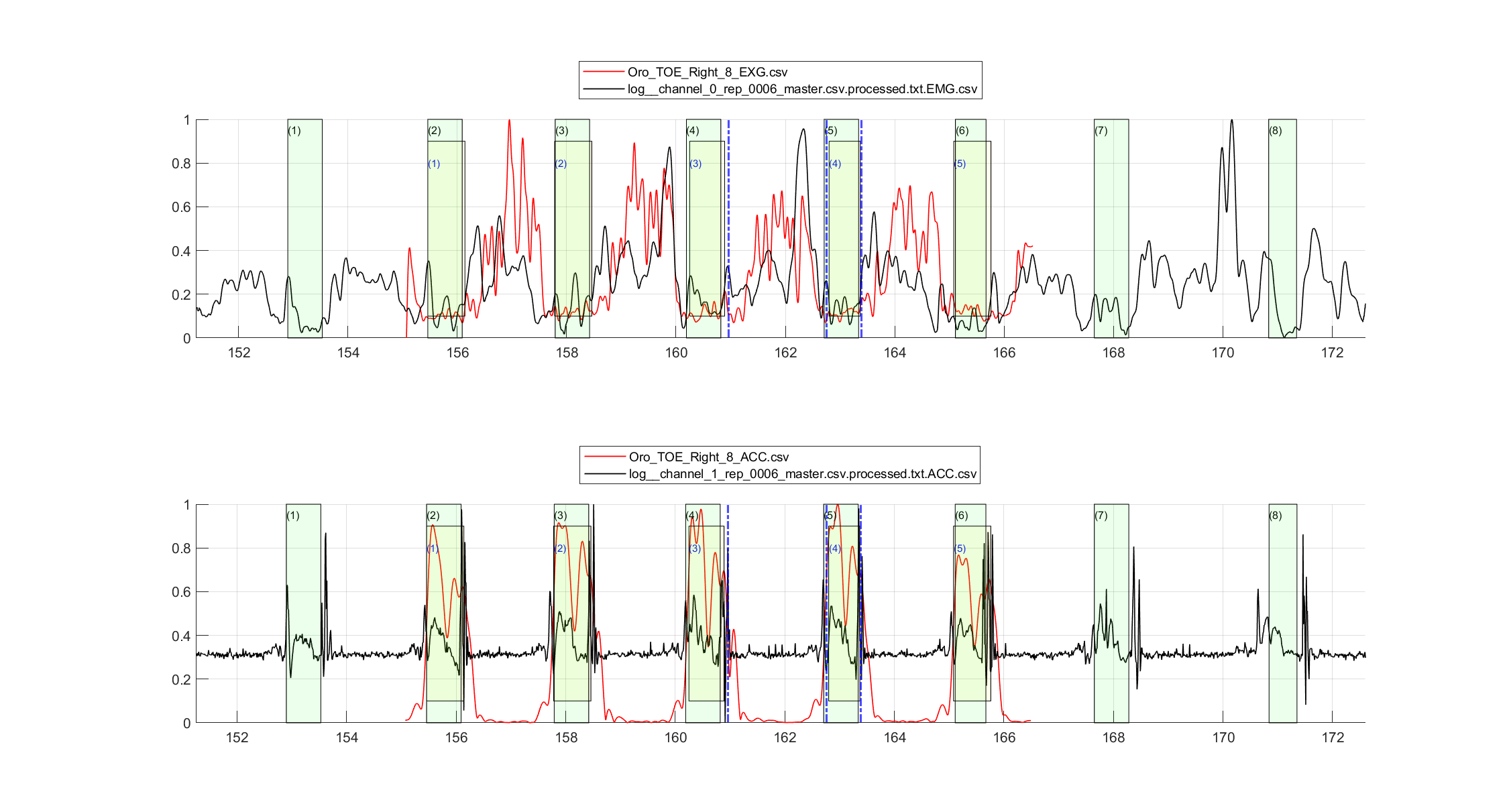

Supplement: Supplementary file 1 [file sensors-22-04957-s001.zip › Part 2 - 3D CGA vs oro sensor system data partitioning/Patient 5-2 shoes/Figure_Oro_TOE_Right_8.png]
